# Comparative genomic analysis of alloherpesviruses: Exploring an available genus/species demarcation proposal and method

**DOI:** 10.1016/j.virusres.2023.199163

**Published:** 2023-07-26

**Authors:** Wenjie Zhang, Ran Wang, Xiaoxia Zou, Congwei Gu, Qian Yang, Manli He, Wudian Xiao, Lvqin He, Mingde Zhao, Zehui Yu

**Affiliations:** a Laboratory Animal Center, Southwest Medical University, Luzhou Sichuan, PR China; b Model Animal and Human Disease Research of Luzhou Key Laboratory, PR China; c Suining First People's Hospital, Suining, PR China; d Scholl of Basic Medical Sciences, Zhejiang University, Hangzhou, PR China

**Keywords:** Alloherpesviruses, Viral classification, Phylogenetic analysis, ANI/AAI, Dot-plot analysis, Core-pan analysis

## Abstract

The family *Alloherpesviridae* contains herpesviruses of fish and amphibians. Due to the significant economic losses to aquaculture that herpesviruses can cause, the primary areas of research interest are concerning their pathogenesis and prevention. Despite alloherpesvirus genomic sequences becoming more widely accessible, methods regarding their genus/species classification are still relatively unexplored. In the present study, the phylogenetic relationships between 40 completely sequenced alloherpesviruses were illustrated by the viral proteomic tree (ViPTree), which was divided into three monophyletic groups, namely *Cyprinivirus, Ictalurivirus* and *Batrachovirus*. Additionally, average nucleotide identity (ANI) and average amino acid identity (AAI) analyses were performed across all available sequences and clearly displayed species boundaries with the threshold value of ANI/AAI set at 90%. Subsequently, core-pan analysis uncovered 809 orthogroups and 11 core genes shared by all 40 alloherpesvirus genome sequences. For the former, a 15 percent identity depicts a clear genus boundary; for the latter, 8 of them may be qualified for phylogenetic analysis based on amino acid or nucleic acid sequences after being verified using maximum likelihood (ML) or neighbor-joining (NJ) phylogenetic trees. Finally, although the dot plot analysis was valid for the members within *Ictalurivirus*, it was unsuccessful for *Cyprinivirus* and *Batrachovirus*. Taken together, the comparison of individual methodologies provides a wide range of alternatives for alloherpesviruses classification under various circumstances.

## Highlights

• Alloherpesviruses were divided into Cyprinivirus, ictalurivirus and batrachovirus based on genome.
• ANI and AAI analyses clearly displayed species boundaries.
• The four genes which are suitable for phylogenetic analysis tree were selected.

## 1 Introduction

According to latest International Committee on Taxonomy of Viruses (ICTV) master Species list 2022.v1, the order Herpesvirales now consists of 3 families (*Orthoherpesviridae, Alloherpesviridae*, and *Malacoherpesviridae*), with each family containing viruses that infect a wide range of hosts (Boutier et al., 2015). The family *Alloherpesviridae* contains 4 genera (*Salmonivirus, Ictalurivirus, Cyprinivirus* and *Batrachovirus*) and 13 species. While the genus *Batrachovirus* encompasses viruses that infect amphibians, viruses in the other three genera are fatal and contagious for fish (Waltzek et al., 2009). In addition to endangering wild amphibians, they also cause serious economic losses to the aquaculture industry (Boutier et al., 2015; Origgi et al., 2017b).

*Salmonid herpesvirus 1, 2* and *3* (SalHV-1, −2 and −3) are three official species recognized by the ICTV as belonging to the genus *Salmonivirus*, which infect salmonid fishes (Waltzek et al., 2009). SalHV-4 and SalHV-5 were two new isolates proposed as members of the genus *Salmonivirus* through single-gene molecular biology identification (Glenney et al., 2016; Doszpoly et al., 2013). Unfortunately, no complete genome sequences for the genus *Salmonivirus* are available in public databases. The genus *Ictalurivirus* contains three ICTV official species: *Acipenserid herpesvirus 2* (AciHV-2), *Ictalurid herpesvirus 1* and *2* (IcHV-1 and IcHV-2). AciHV-2 infects sturgeons while IcHV-1 and IcHV-2 infect s channel catfish (Waltzek et al., 2009). To date, the sequences of IcHV-1 and IcHV-2 have been sequenced completely. The genus *Cyprinivirus* contains four ICTV official species: *Cyprinid herpesvirus 1, 2* and *3* (CyHV-1, −2 and −3) and *Anguillid herpesvirus 1* (AngHV-1). CyHV-1 and CyHV-3, CyHV-2 and AngHV-1 infect *Cyprinus carpio* species, *Carassius* species and Japanese/European eels, respectively (Waltzek et al., 2009; Donohoe et al., 2021). All four species in the genus *Ictalurivirus* have entire genome sequences that are currently accessible in NCBI databases. Unlike previous three genera, the genus *Batrachovirus* includes viruses that infect frogs (Waltzek et al., 2009; Origgi et al., 2017b). Although the NCBI database has the published complete gene sequences of the three species members of genus *Batrachovirus*, comparative genomic studies on viruses of this genus have rarely been reported.

The development of high-throughput sequencing technology coupled with reduced sequencing costs have led to an explosive increase in viral genome data. Genome-based virus taxonomy possesses increasingly clear advantages (Bin Jang et al., 2019). In the meantime, many bioinformatics tools, such as Viral proteomic tree (ViPTree) (Nishimura et al., 2017), Composition vector tree (CVTree) (Zuo, 2021) and PYANI (Pritchard et al., 2016), have also been developed, which greatly promote the development of studies looking into virus taxonomy and evolution. However, genome-based taxonomic approaches for the family *Alloherpesviridae* have rarely been investigated. In addition, the Genus/Species demarcation criteria for the family *Alloherpesviridae* have not been released in the “ICTV report on virus classification” (https://talk.ictvonline.org/ictv-reports/ictv_online_report/). To this end, this study will explore a practical and well-defined taxonomic approach through comparative genomic analysis of alloherpesviruses.

## 2 Materials and methods

### 2.1 Genomic sequences

As of April 9, 2022, the complete genome sequences of 40 alloherpesviruses have been published in NCBI virus database (https://www.ncbi.nlm.nih.gov/labs/virus/vssi/#/). Detailed information of the sequences used for the analysis in this study are provided in Supplementary Table 1. The genomic sequences were downloaded from NCBI using customized scripts based on Entrez Programming Utilities (Supplementary Table 1). The whole genome sequences were predicted and annotated using Prokka v1.12 with default parameters (Seemann, 2014). The genome length and GC content were calculated by SeqKit Toolkit (Shen et al., 2016).

### 2.2 Phylogenetic tree based on genome

Two phylogenetic trees (Viral proteomic tree and Composition vector tree) based on whole-genome sequences were constructed using the ViPTree v1.1.2 and CVTree v4.0, respectively (Nishimura et al., 2017; Zuo, 2021). The detailed parameters are shown in Supplementary Table 2. Newick files were transferred to iTOL online tool for visualization and annotation (Letunic and Bork, 2021).

### 2.3 ANI and AAI analysis

The average nucleotide identity (ANI) values were calculated using the BLAST algorithm and MUMmer algorithm (Richter and Rosselló-Móra, 2009). The calculation process was implemented by Python module PYANI v0.2.7 (http://widdowquinn.github.io/pyani/) (Pritchard et al., 2016), and ‘‘FRAGSIZE’’ parameter was set to 200 with other default parameters. The ANI values calculated using the BLAST algorithm were called “ANIb”, and those calculated with MUMmer algorithm were ‘‘ANIm’’. The average amino acid identities (AAI) were calculated using CompareM v0.1.2 (https://github.com/dparks1134/CompareM). The pairwise ANI or ANI values between the 40 alloherpesviruses were visualized using the pheatmap package in R.

### 2.4 Dot-plot analyses

The steps of Dot-plot analyses: (1) Amino acid sequences were predicted by Prokka into “all.fa” file using Linux cat command. (2) All-versus-all BLASTP was done using BLAST v2.5.0+ (Camacho et al., 2009) (code: “blastp -query all.fa -db index/all -out all.blast -evalue 1e-5 -outfmt 6 && blastp -query all.fa -db index/all -out all.blast -evalue 1e-5 -outfmt 6″). (3) The “all.blast” file and the annotation file (gff file generated by Prokka) were used as input files to generate dot-plot images using MCScanX software (Wang et al., 2012). For the details of step (3), please refer to MCScanx instructions (https://github.com/wyp1125/MCScanX) and our previous published paper (Yu et al., 2021).

### 2.5 Core-pan analysis

The all-versus-all proteins BLASTP network diagram was generated using Cytoscape v3.9.1 (“all.blast” file is input file) (Shannon et al., 2003). The Venn diagrams of protein clusters were drawn by using igraph v1.2.10 and Venn package in R (Csardi and Nepusz, 2006; Swinton, 2013).

### 2.6 Shared orthogroups visualization

The percent identity of shared orthogroups (*p_orthogroups*) between two genomes was computed by following formula. The *p_orthogroups* values were visualized using the pheatmap package in R.

$$p{\_}_{orthogroups}=2\times \frac{\text{nu}m_{shared\_orthogroups}}{\text{nu}m_{cds\_A}+\text{nu}m_{cds\_B}}\times 100\%$$

Num_cds_A_ and num_cds_B_ indicate the number of coding sequences in the genomes of A and B, respectively. Num_shared_orthogroups_ indicates the number of shared orthogroups between A and B.

### 2.7 Phylogenetic tree based on single core-genes

Multiple alignments of core-genes as defined in Section 2.5 were performed using MAFFT v7.490 with default parameters (Katoh and Standley, 2013). Construction of NJ-trees (neighbor-joining method phylogenetic trees) and ML-tree (Maximum likelihood method phylogenetic trees) were done using MEGA v11.0.11 and IQ-TREE v2.2.0-beta (Tamura et al., 2021; Nguyen et al., 2015), respectively. The detailed parameters are shown in Supplementary Table 2. Newick files were transferred to ggtree package in R for visualization and annotation (Yu, 2020).

## 3 Results

### 3.1 Phylogenetic tree based on genome

**Fig. 1:**
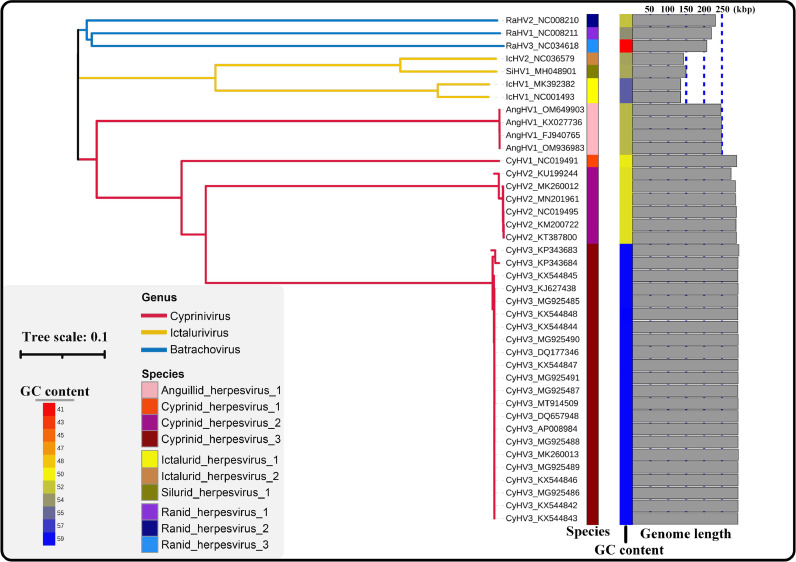
Phylogenetic tree based on genome using ViPTree. Branch length indicates evolutionary distance and branch color indicates genus-level classification. GC content and genome length are annotated on the right side of the tree.

The ViPTree is an analysis tool for constructing phylogenetic tree based on genome-wide similarities. Currently, it is commonly used for prokaryotic viruses (phages) (Buckley et al., 2021; Biosca et al., 2021). In order to explore whether ViPTree is suitable for classification for the members of family *Alloherpesviridae*, the phylogenetic tree of 40 alloherpesviruses was constructed using ViPTree with default parameters. The tree showed that members of the same genus or species can form a monophyletic group (Fig. 1), which indicates that the ViPTree is applicable for phylogenetic analysis of alloherpesviruses. In addition, we also tried another alignment-free method, CVTree (Figure S1). The result showed that the members of *Batrachovirus* genus cannot form a monophyletic group.

### 3.2 ANI and AAI analysis

**Fig. 2:**
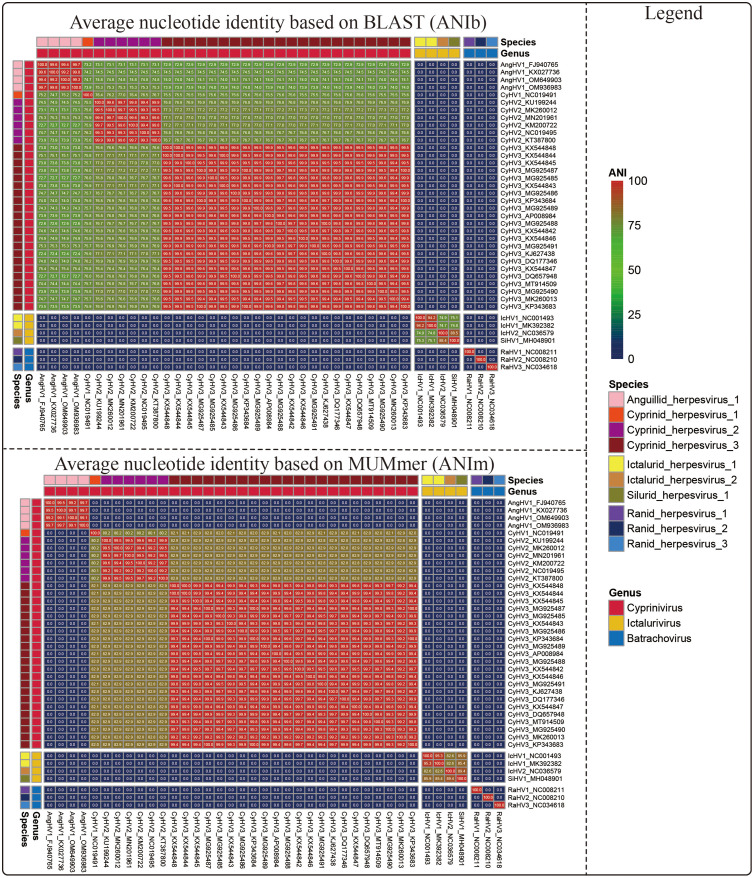
The heatmap showing pairwise ANI values between 40 alloherpesviruses.

The average nucleotide identity (ANI) between two genomes can reflect the degree of evolutionary distance between the compared genomes and offers a quantifiable and relatively stable species boundary (Richter and Rosselló-Móra, 2009). In our study, we used two algorithms to calculate ANI values between 40 alloherpesviruses (Flowchart detailing the calculation of ANIb is shown Figure S2). The heatmap of ANI showed that a threshold of 90% ANI was suitable as the putative species-level boundary for alloherpesviruse (ANI values between all members of same species were more than 90% and members of a given species share less than 90% ANI with members of other species; Fig. 2). In addition, the heatmap also showed average amino acid identity (AAI) values between 40 alloherpesviruses (Fig. S3). Similarly, 90% AAI can also be used as the species-level boundary (Figure S3).

### 3.3 Dot-plot analyses

**Fig. 3:**
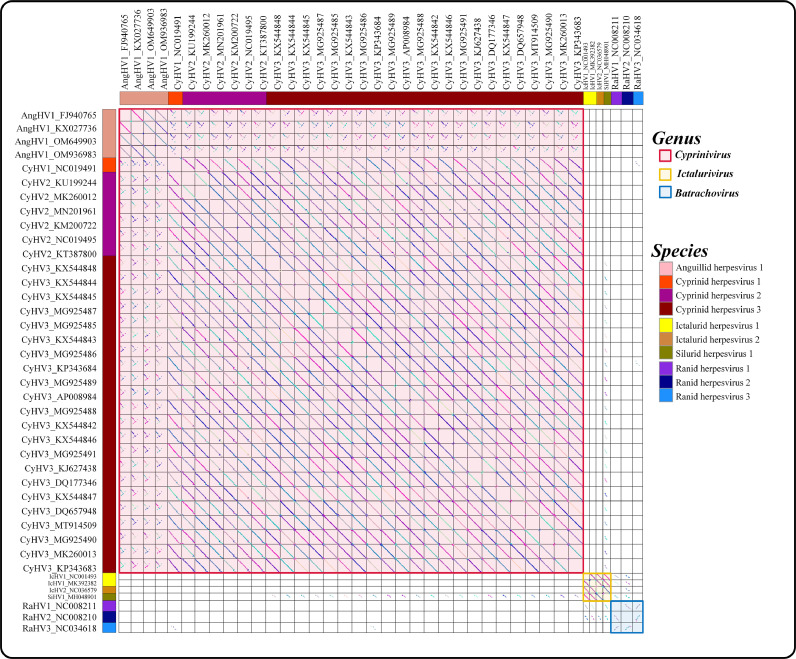
The dot-plot showing the collinearity of 40 alloherpesviruses. Each corresponding box represents the collinear comparison of two viruses, and line segments in the boxes represent regions of colinearity. The diagonal line in the box indicates that the two genomes are completely collinear. The blank boxes indicate that the two corresponding genomes do not share collinear/similarity fragments (evalue 1e-5). The colours of lines in a box are to distinguish between different collinear regions. The colours between blocks are irrelevant.

Dot-plot analyses offer a general overview of genomic organization. The results of the dot-plot showed that gene order was conserved among the members of *Ictalurivirus* (Fig. 3 yellow box), but gene order was not conserved among the members of *Cyprinivirus* (Fig. 3 red box) and *Batrachovirus* (Fig. 3 blue box). Within the genus *Cyprinivirus*, the gene orders between CyHV1–3 are highly conserved, while the gene orders between AngHV1 and CyHV1–3 are conserved at a lower level (Fig. 3 and Figure S4).

In addition, dot-plot analyses can also provide a general overview of gene similarity between genomes by filtering percent of identical matches values (p-ident), which may shed light on the taxonomy of alloherpesviruses. By comparing dot-plot graphs with different thresholds of identical matches, we found that the threshold of 30% and 95% p-ident can be used as putative genus-level and species-level boundaries for alloherpesviruse (Figure S5), respectively. The members of a given genus share less than 30% collinear/similarity fragments with members of other genera, and the members of a given species share less than 95% collinear/similarity fragments with members of other species.

### 3.4 Core-pan analysis

**Fig. 4:**
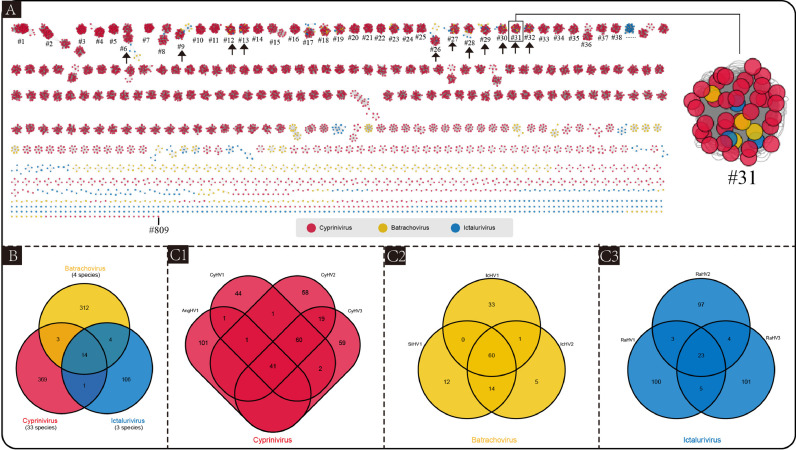
Core-pan analysis of 40 alloherpesviruses. A: Protein-sharing networks. protein sequences (nodes) are color-coded according to genus level classification. The edge indicates protein sequence homology at both ends (E-value < 10^−5^). The clusters formed by nodes and edges are orthologous gene groups (orthogroups). Black arrows represent core-genes clusters. The #31 is an enlarged core-genes clusters, and it contain all alloherpesviruses (40) major capsid protein sequences. B and C: Venn diagram for orthogroups of protein sequences. The numbers indicate the number of orthogroups.

To define the core set of alloherpesvirus genes, the core-pan analysis of alloherpesvirus genomes was performed. Firstly, all protein sequences (7125 sequences) within the 40 alloherpesvirus genomes were subjected to all-versus-all BLASTp, generating a network diagram based on BLASTp result. The network diagram showed that the 7125 protein sequences were grouped in 809 orthogroups (Fig. 4), and the core-gene (the genes present in all 40 alloherpesvirus genomes) orthogroups were indicated by black arrows. The cluster of core-genes includes Allo37 (#6), Terminase (#9), DNA polymerase (#12), Allo56 (#13), helicase-primase subunit (#26 and #32), Allo60 (#27), Allo64 (#28), capsid maturation protease (#29), capsid triplex protein (#30) and major capsid protein (#31). The length, number and location of core-gene sequences are shown in Table S3 and Table S4 (The core-gene sequences are provided in supplementary material).

The number of shared or unique orthogroups between genus/species was displayed by a Venn diagram (Fig. 4B and C). The Fig. 4C showed that the four groups had a relatively high number of unique orthogroups (AngHV1=97, RaHV1=100, RaHV2=97 and RaHV3=101). Meanwhile, the evolutionary distance of these four species was also relatively far, which suggests that the loss or gain of genes is related to alloherpesvirus evolution.

### 3.5 Shared orthogroups visualization

**Fig. 5:**
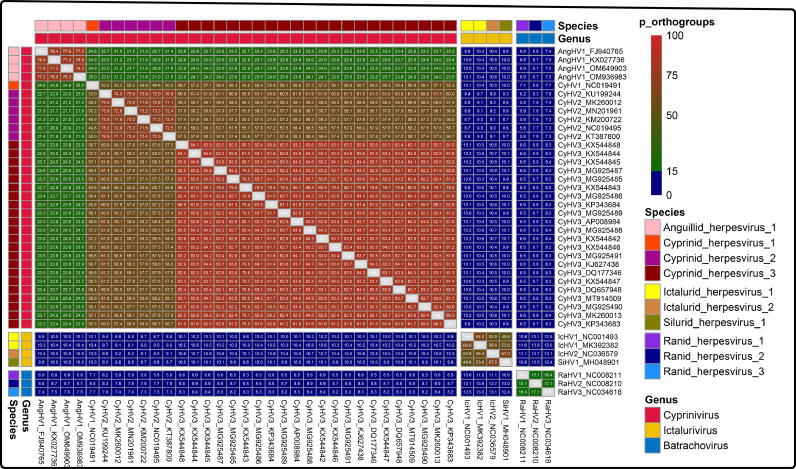
The heatmap showing pairwise p_orthogroups values between 40 alloherpesviruses.

Comparing the percent identity of orthologous gene cut-offs is common approach to define taxonomic classification prokaryotic virus (Bin Jang et al., 2019), and loss or gain of genes is related to alloherpesvirus evolution. To explore the applicability of this approach to alloherpesvirus, the percent identity of shared orthogroups (p_orthogroups) between alloherpesviruses was computed. The heatmap of p_orthogroups showed the threshold of 15% p_orthogroups can be used as genus-level boundaries for alloherpesviruse (Fig. 5, p_orthogroups between all members of same genus-level was more than 15% while members of a given genus share less than 15 p_orthogroups with members of other genera).

### 3.6 Phylogenetic tree based on single core-genes

The above methods are all based on the genome of the virus, but the phylogenetic analysis of individual or concatenated multiple gene sequences is more convenient for the preliminary identification of novel virus isolates. To select qualified core-genes and methods, we performed phylogenetic analysis of 11 alloherpesvirus core-genes using different methods and sequence types. The phylogenetic trees based on single core-genes are shown in Figure S6. The criteria for judging which phylogenetic trees are qualified are that the members of same genus or species can form a monophyletic group (results shown in Table 1, "Yes" means the tree meets the criteria).The amino acid sequences of four genes (Allo37, Terminase, Allo64 and Capsid maturation protease) and the nucleic acid sequences of two genes (DNA polymerase and Major capsid protein) were qualified for phylogenetic analysis based on the ML method. Meanwhile, five core genes may serve as markers for phylogenetic tree construction based on the NJ method (Figure S6 and Table 1). These results suggest that selection of sequence types and method are important for alloherpesvirus single-gene phylogenetic analysis.

**Table 1:** The comparative qualification of alloherpesvirus core-genes for phylogenetic tree.

Orthogroups (Annotation)	ML method using IQ-TREE		ML method using MEGA		NJ method	
	AA	NA	AA	NA	AA	NA
#6* (Allo37)	no	no	yes	no	yes	no
#9* (Terminase)	no	no	yes	no	no	no
#12* (DNA polymerase)	no	yes	no	yes	no	no
#13* (Allo56)	no	no	no	no	no	no
#26 (Helicase-primase subunit)	no	no	no	no	no	no
#27 (Allo60)	no	no	no	no	yes	no
#28 (Allo64)	no	no	yes	no	yes	no
#29(Capsid maturation protease)	no	no	no	no	no	no
#30 (Capsid triplex protein)	no	no	no	no	no	no
#31 (Major capsid protein)	no	yes	no	yes	no	yes
#32 (Helicase-primase subunit)	no	no	no	no	no	no

The “*” indicates multiple copies and the longest copies have been selected.

AA indicates amino acid sequences, and NA indicates nucleic acid sequences.

Yes and no indicates whether the tree meets the criteria.

## 4 Discussion

Fish alloherpesviruses are the cause of a number of serious diseases in aquaculture. They persist in infected communities in an inapparent latent state, enabling the spread to hosts through recrudescence and horizontal transmission or to offspring through vertical transmission. Currently, the official taxonomical criteria of alloherpesviruses is still pending. Thus, in view of the rising availability of viral genomes and the growing need to establish their evolutionary background and taxonomic classification, a comprehensive genus/species demarcation method is urgently needed to fill gaps.

In the present study, various methods have been validated to investigate the possibilities for genus/species demarcation of the 40 alloherpesviruses. Among them, both phylogenetic analysis (ViPtree) and dot-plot analysis exhibited obvious boundaries when applied at both the species and genus ranks (Table 2). The ViPTree is a newly proposed server, which can generate viral classification trees based on genome-wide similarities. Owing to its simplicity and high efficiency as compared with gene-based phylogenic analysis (Wu et al., 2009), ViPTree has already been used in several studies related to phages (Adriaenssens et al., 2015; Bhunchoth et al., 2016; Bellas et al., 2015). Herein, the obvious monophylies of genus *Cyprinivirus, Ictalurivirus* and *Batrachovirus* and branches of indicated species as showcased by the ViPTree strongly support its potential in being used for genus and species classification within *Allohepesviridae* family, although it cannot be quantified. Similarly, the dot-plot analysis exhibited a clear genus/species distribution by setting at specific parameters (30% and 95% identical co-linearity fragments at genus-level and species-level, respectively, Fig. S5). However, the cumbersome steps and parameters optimization process make it inconvenient for users. In our previous study, we have demonstrated the potential of ANI analysis for viral classification at species rank (Deng et al., 2022). Herein, although both ANI and AAI analyses were not suitable at the genus rank, they were applicable for species rank demarcation (Figs. 2, and S3), which paves the way for broad use in viral species classification. Similarly, the results of core-pan analysis showed limited application at genus rank, but not at species rank (Fig. 5). Interestingly, although the CV tree has been widely used for investigation for evolutionary relatedness of virus, chloroplasts and fungi (Wu et al., 2009; Choi and Kim, 2017), in the present study it failed to form a monophyletic clade at both species and genus rank. Apart from these, an interesting phenomenon was observed. The results of phylogenetic analysis (Fig. 1) and dot-plot analysis (Fig. 3) showed the close relatedness between SiHV1 and IcHV1 and 2, indicating their potential affiliation. Taken together, the above approaches exhibit different advantages and disadvantages, indicating that the practical use of them should include consideration of realistic circumstances. In addition, according to ICTV 9th Report about herpesvirus species (https://ictv.global/report_9th/dsDNA/Herpesvirales), Species demarcation criteria should also consider epidemiological and biological characteristics (e.g. host identity, pathogenic and epidemiological properties, and the lack of occurrence of natural recombinants). The viruses should be classified as new species if have distinct epidemiological and biological characteristics, even if they do not exceed thresholds at the genetic level.

**Table 2:** Genus/Species demarcation proposal based on genome sequences for the family *Alloherpesviridae*.

Method	Algorithms / Tools	Demarcation proposal		Disadvantages
		Species-level	Genus-level	
Phylogenetic analysis	VipTree	form a monophyletic group	form a monophyletic group	Cannot quantify
	CVtree	Not applicable	Not applicable	Not applicable
Genomic distance	ANI/AAI	90% ANI	Not applicable	Only applicable for species demarcation
Genomic organization	BLAST and MCScanX	95% identical collinearity fragment	30% identical collinearity fragment	Complex analysis process
Core-pan analysis	The percent identity of shared orthogroups	Not applicable	15% identity of orthologous gene	Only applicable for genus demarcation

Classifying viruses by means that necessitate prior knowledge of their complete genomic sequences is time-consuming. In contrast, the use of marker genes shared by all genomic sequences or the concatenated sequences of them makes it easier. It has been reported that while alloherpesvirus genomes share 40–60 homologous genes within a genus, only 12 genes have been found shared by all completely sequenced alloherpesviruses (Davison et al., 2013). Interestingly, in the present study, 11 conserved genes have been identified among 40 completely sequenced alloherpesvirus genomes, sharing the similarities in DNA polymerase and Terminase, two sequences frequently used for alloherpesvirus phylogenetic analysis (Origgi et al., 2017a; Marcos-Lopez et al., 2012). However, protein Allo54 was not involved in our selection. A comparison of each gene has been performed to verify the feasibility of phylogenetic analysis based on these candidates (Table 1). Surprisingly, although the deduced amino acid sequences of Terminase were applicable for ML tree using MEGA, the trial based on amino acid sequences of DNA polymerase was unsuccessful, which was different from previous reports (Origgi et al., 2017a; Marcos-Lopez et al., 2012). In addition, Allo37/Allo64 (amino acid sequences) and major capsid protein (nucleic acid sequences) qualified to be used in connstrunction of the ML tree. Similarly, NJ tree construction using Allo37, Allo60 and Allo64 (amino acid sequences), as well as major capsid protein (nucleic acid sequences), revealed their potential to serve as conserved genes for phylogenetic analysis. In conclusion, our findings update the pool of the conserved genes, while providing a theoretical basis for the selection of conserved genes under different circumstances.

## CRediT authorship contribution statement

**Wenjie Zhang:** Conceptualization, Visualization, Writing – original draft, Writing – review & editing. **Ran Wang:** Writing – original draft, Writing – review & editing. **Xiaoxia Zou:** Conceptualization, Visualization, Data curation. **Congwei Gu:** Formal analysis, Writing – review & editing. **Qian Yang:** Data curation. **Manli He:** Data curation. **Wudian Xiao:** Formal analysis. **Lvqin He:** Data curation. **Mingde Zhao:** Formal analysis, Writing – original draft. **Zehui Yu:** Conceptualization, Visualization, Formal analysis, Writing – review & editing, Funding acquisition.

## Declaration of Competing Interest

The authors declare that they have no known competing financial interests or personal relationships that could have appeared to influence the work reported in this paper.

## Data Availability

Data will be made available on request.

## Appendix Supplementary materials

Image, application 1

Image, application 2

Image, application 3

Image, application 4

Image, application 5

Image, application 6

Image, application 7

Image, application 8

## References

[bib0001] Adriaenssens E.M., Edwards R., Nash J.H., Mahadevan P., Seto D., Ackermann H.W., Lavigne R., Kropinski A.M. (2015). Integration of genomic and proteomic analyses in the classification of the Siphoviridae family. Virology.

[bib0002] Bellas C.M., Anesio A.M., Barker G. (2015). Analysis of virus genomes from glacial environments reveals novel virus groups with unusual host interactions. Front. Microbiol..

[bib0003] Bhunchoth A., Blanc-Mathieu R., Mihara T., Nishimura Y., Askora A., Phironrit N., Leksomboon C., Chatchawankanphanich O., Kawasaki T., Nakano M., Fujie M., Ogata H., Yamada T. (2016). Two asian jumbo phages, ϕRSL2 and ϕRSF1, infect Ralstonia solanacearum and show common features of ϕKZ-related phages. Virology.

[bib0004] Bin Jang H., Bolduc B., Zablocki O., Kuhn J.H., Roux S., Adriaenssens E.M., Brister J.R., Kropinski A.M., Krupovic M., Lavigne R., Turner D., Sullivan M.B. (2019). Taxonomic assignment of uncultivated prokaryotic virus genomes is enabled by gene-sharing networks. Nat. Biotechnol..

[bib0005] Biosca E.G., Català-Senent J.F., Figàs-Segura À., Bertolini E., López M.M., Álvarez B. (2021). Genomic analysis of the first european bacteriophages with depolymerase activity and biocontrol efficacy against the phytopathogen. Viruses.

[bib0006] Boutier M., Ronsmans M., Rakus K., Jazowiecka-Rakus J., Vancsok C., Morvan L., Peñaranda M.M.D., Stone D.M., Way K., van Beurden S.J., Davison A.J., Vanderplasschen A. (2015). Cyprinid herpesvirus 3: an archetype of fish alloherpesviruses. Adv. Virus Res..

[bib0007] Buckley D., T. Odamaki, J. Xiao, J. Mahony, D. van Sinderen and F. Bottacini, 2021: Diversity of human-associated bifidobacterial prophage sequences. Microorganisms*,* 9. 10.3390/microorganisms9122559 PMC8705816 34946160

[bib0008] Camacho C., Coulouris G., Avagyan V., Ma N., Papadopoulos J., Bealer K., Madden T.L. (2009). BLAST+: architecture and applications. BMC Bioinf..

[bib0009] Choi J., Kim S.H. (2017). A genome tree of life for the fungi kingdom. Proc. Natl. Acad. Sci..

[bib0010] Csardi G., Nepusz T. (2006). The igraph software package for complex network research. Interjournal Complex Syst..

[bib0012] Davison A.J., Kurobe T., Gatherer D., Cunningham C., Korf I., Fukuda H., Hedrick R.P., Waltzek T.B. (2013). Comparative genomics of carp herpesviruses. J. Virol..

[bib0013] Deng Z., Xia X., Deng Y., Zhao M., Gu C., Geng Y., Wang J., Yang Q., He M., Xiao Q. (2022). ANI analysis of poxvirus genomes reveals its potential application to viral species rank demarcation. Virus Evol..

[bib0014] Donohoe O., Zhang H., Delrez N., Gao Y., Suárez N.M., Davison A.J., Vanderplasschen A. (2021). Genomes of anguillid herpesvirus 1 strains reveal evolutionary disparities and low genetic diversity in the genus. Microorganisms.

[bib0015] Doszpoly A., Karaseva T.A., Waltzek T.B., Kalabekov I.M., Shchelkunov I.S. (2013). Atlantic salmon papillomatosis in Russia and molecular characterization of the associated herpesvirus. Dis. Aquat. Org..

[bib0016] Glenney G.W., Barbash P.A., Coll J.A. (2016). Initial detection and molecular characterization of namaycush herpesvirus (salmonid herpesvirus 5) in lake trout. J. Aquat. Anim. Health.

[bib0017] Katoh K., Standley D.M. (2013). MAFFT multiple sequence alignment software version 7: improvements in performance and usability. Mol. Biol. Evol..

[bib0018] Letunic I., Bork P. (2021). Interactive Tree Of Life (iTOL) v5: an online tool for phylogenetic tree display and annotation. Nucleic. Acids. Res..

[bib0019] Marcos-Lopez M., Waltzek T.B., Hedrick R.P., Baxa D.V., Garber A.F., Liston R., Johnsen E., Forward B.S., Backman S., Ferguson H.W. (2012). Characterization of a novel alloherpesvirus from Atlantic cod (Gadus morhua). J. Vet. Diagn. Invest..

[bib0020] Nguyen L.T., Schmidt H.A., von Haeseler A., Minh B.Q. (2015). IQ-TREE: a fast and effective stochastic algorithm for estimating maximum-likelihood phylogenies. Mol. Biol. Evol..

[bib0021] Nishimura Y., Yoshida T., Kuronishi M., Uehara H., Ogata H., Goto S. (2017). ViPTree: the viral proteomic tree server. Bioinformatics.

[bib0022] Origgi F., Schmidt B., Lohmann P., Otten P., Akdesir E., Gaschen V., Aguilar-Bultet L., Wahli T., Sattler U., Stoffel M.H. (2017). Ranid herpesvirus 3 and proliferative dermatitis in free-ranging wild common frogs (Rana temporaria). Vet. Pathol..

[bib0023] Origgi F.C., Schmidt B.R., Lohmann P., Otten P., Akdesir E., Gaschen V., Aguilar-Bultet L., Wahli T., Sattler U., Stoffel M.H. (2017). Ranid herpesvirus 3 and proliferative dermatitis in free-ranging wild common frogs (Rana Temporaria). Vet. Pathol..

[bib0024] Pritchard L., Glover R.H., Humphris S., Elphinstone J.G., Toth I.K. (2016). Genomics and taxonomy in diagnostics for food security: soft-rotting enterobacterial plant pathogens. Anal. Methods.

[bib0025] Richter M., Rosselló-Móra R. (2009). Shifting the genomic gold standard for the prokaryotic species definition. Proc. Natl. Acad. Sci. U. S. A..

[bib0026] Seemann T. (2014). Prokka: rapid prokaryotic genome annotation. Bioinformatics.

[bib0027] Shannon P., Markiel A., Ozier O., Baliga N.S., Wang J.T., Ramage D., Amin N., Schwikowski B., Ideker T. (2003). Cytoscape: a software environment for integrated models of biomolecular interaction networks. Genome Res..

[bib0028] Shen W., Le S., Li Y., Hu F. (2016). SeqKit: a cross-platform and ultrafast toolkit for FASTA/Q file manipulation. PLoS One.

[bib0029] Swinton J. (2013). Venn diagrams in R with the Vennerable package.

[bib0030] Tamura K., Stecher G., Kumar S. (2021). MEGA11: molecular evolutionary genetics analysis version 11. Mol. Biol. Evol..

[bib0031] Waltzek T.B., G.O. Kelley, M.E. Alfaro, T. Kurobe, A.J. Davison and R.P. Hedrick, 2009: phylogenetic relationships in the family alloherpesviridae. 10.3354/dao02023 19565695

[bib0032] Wang Y., Tang H., Debarry J.D., Tan X., Li J., Wang X., Lee T.H., Jin H., Marler B., Guo H., Kissinger J.C., Paterson A.H. (2012). MCScanX: a toolkit for detection and evolutionary analysis of gene synteny and collinearity. Nucleic. Acids. Res..

[bib0033] Wu G.A., Jun S.R., Sims G.E., Kim S.H. (2009). Whole-proteome phylogeny of large dsDNA virus families by an alignment-free method. Proc. Natl. Acad. Sci..

[bib0034] Yu G. (2020). Using ggtree to visualize data on tree-like structures. Curr. Protoc. Bioinform..

[bib0035] Yu Z., Zhang W., Fu H., Zou X., Zhao M., Liang S., Gu C., Yang Q., He M., Xiao Q., Xiao W., He L., Lü M. (2021). Genomic analysis of Poxviridae and exploring qualified gene sequences for phylogenetics. Comput. Struct. Biotechnol. J..

[bib0036] Zuo G. (2021). CVTree: a parallel alignment-free phylogeny and taxonomy tool based on composition vectors of genomes. Genom. Proteom. Bioinform..

