# Supplementary material for: Comparative genomic analysis of alloherpesviruses: Exploring an available genus/species demarcation proposal and method
Source: Virus Res. 2023 Jul 26;334:199163. doi: 10.1016/j.virusres.2023.199163 (PMC10410580; doi:10.1016/j.virusres.2023.199163)
Supplement: Supplementary file 1 [file mmc1.pdf]

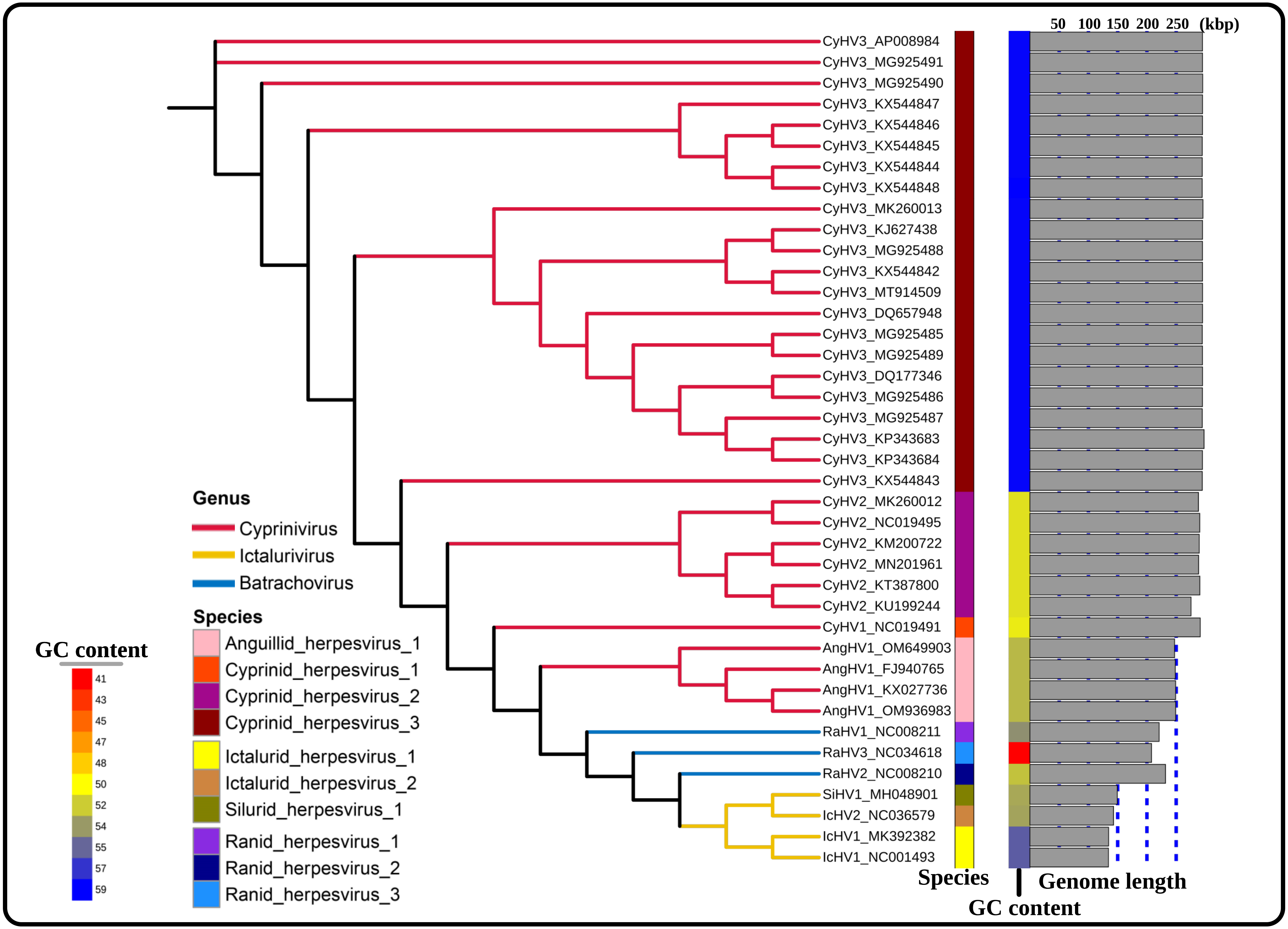

Figure S1 Phylogenetic tree based on genome using CVtree. Branch color indicates genus-level classification. GC content and genome length are annotated on the right side of the tree
