# Supplementary material for: Comparative genomic analysis of alloherpesviruses: Exploring an available genus/species demarcation proposal and method
Source: Virus Res. 2023 Jul 26;334:199163. doi: 10.1016/j.virusres.2023.199163 (PMC10410580; doi:10.1016/j.virusres.2023.199163)
Supplement: Supplementary file 2 [file mmc2.pdf]

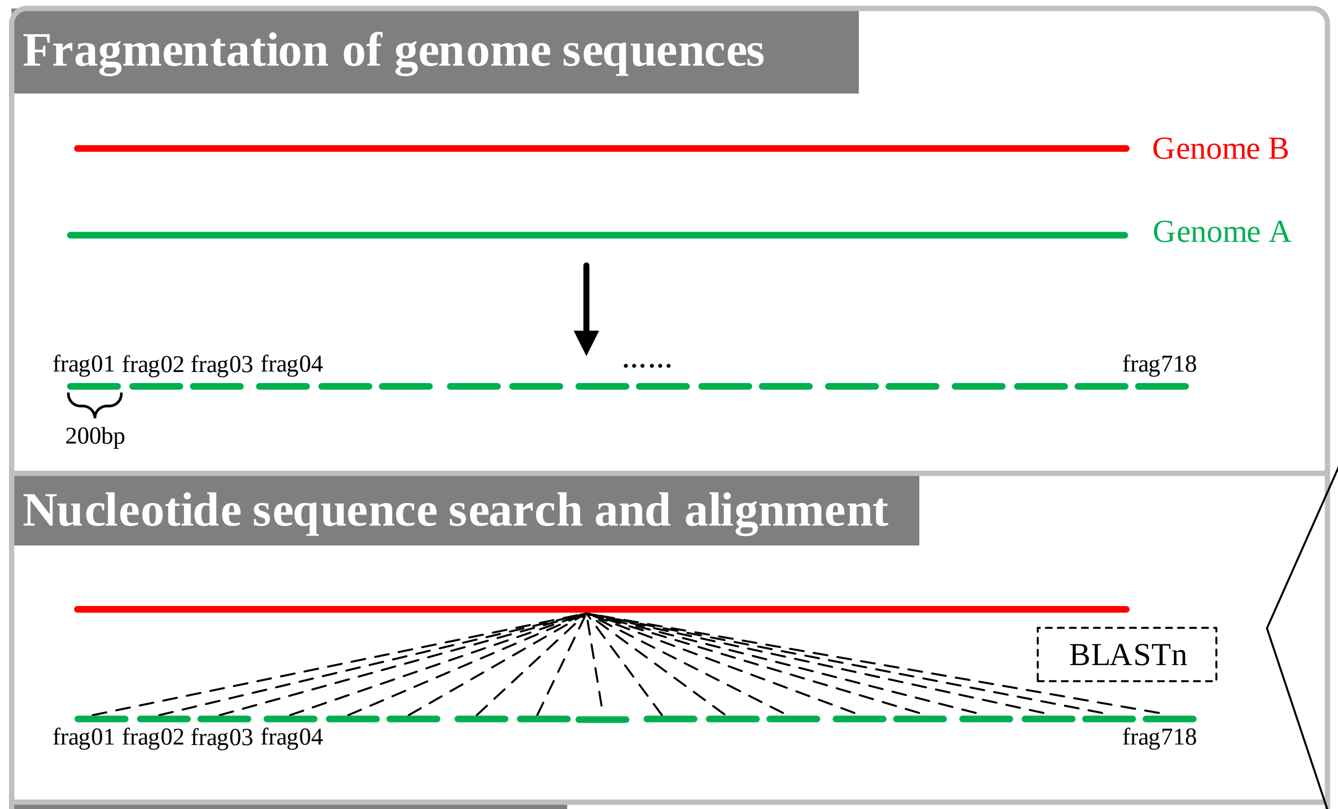

Identity calculation

$$ANI = \frac{\sum_{bbh}(\text{Alignment length} * \text{Percent Identity})}{\sum_{bbh} \text{Alignment length}}$$

The result of BLAST+

qseqid (Genome B)	sseqid (Genome A)	Alignment length	Percent Identity	Bidirectional Best Hits (BBH)	Alignment length * Percent Identity
frag00001	MH048901	70	85.714	yes	5999.98
frag00001	MH048901	199	83.417	no	
frag00002	MH048901	194	88.144	yes	17099.936
frag00003	MH048901	197	92.386	yes	18200.042
frag00004	MH048901	216	78.241	yes	16900.056
frag00706	MH048901	184	81.522	yes	15000.048
......	......	......	......	......	......
frag00709	MH048901	182	81.319	yes	14800.058
frag00710	MH048901	195	92.308	yes	18000.06
frag00711	MH048901	198	92.424	yes	18299.952
frag00712	MH048901	200	92.5	yes	18500
frag00713	MH048901	200	91.5	yes	18300
frag00714	MH048901	173	87.861	yes	15199.953
frag00715	MH048901	123	91.87	yes	11300.01
		$\sum_{bbh}$Alignment length=129624			$\sum_{bbh}$ (Alignment length * Percent Identity)=11470701.13

ANI (NC036579 --> MH048901) = 129624/11470701.13 = 88.49

Figure S2 Flowchart detailing the calculation of ANIb. Taking the calculation of (NC036579 --> MH048901) as an example. the Genome A (NC036579) sequence is initially fragmented into 715 segments of 200 bp, and 715 fragments are then BLAST searched against the Genome B (MH048901) to determine the alignment length and percent identity. The final ANI value are caculated using BBH alignment.
