# Supplementary figures and images for: Comparative genomic analysis of alloherpesviruses: Exploring an available genus/species demarcation proposal and method
Source: Virus Res. 2023 Jul 26;334:199163. doi: 10.1016/j.virusres.2023.199163 (PMC10410580; doi:10.1016/j.virusres.2023.199163)

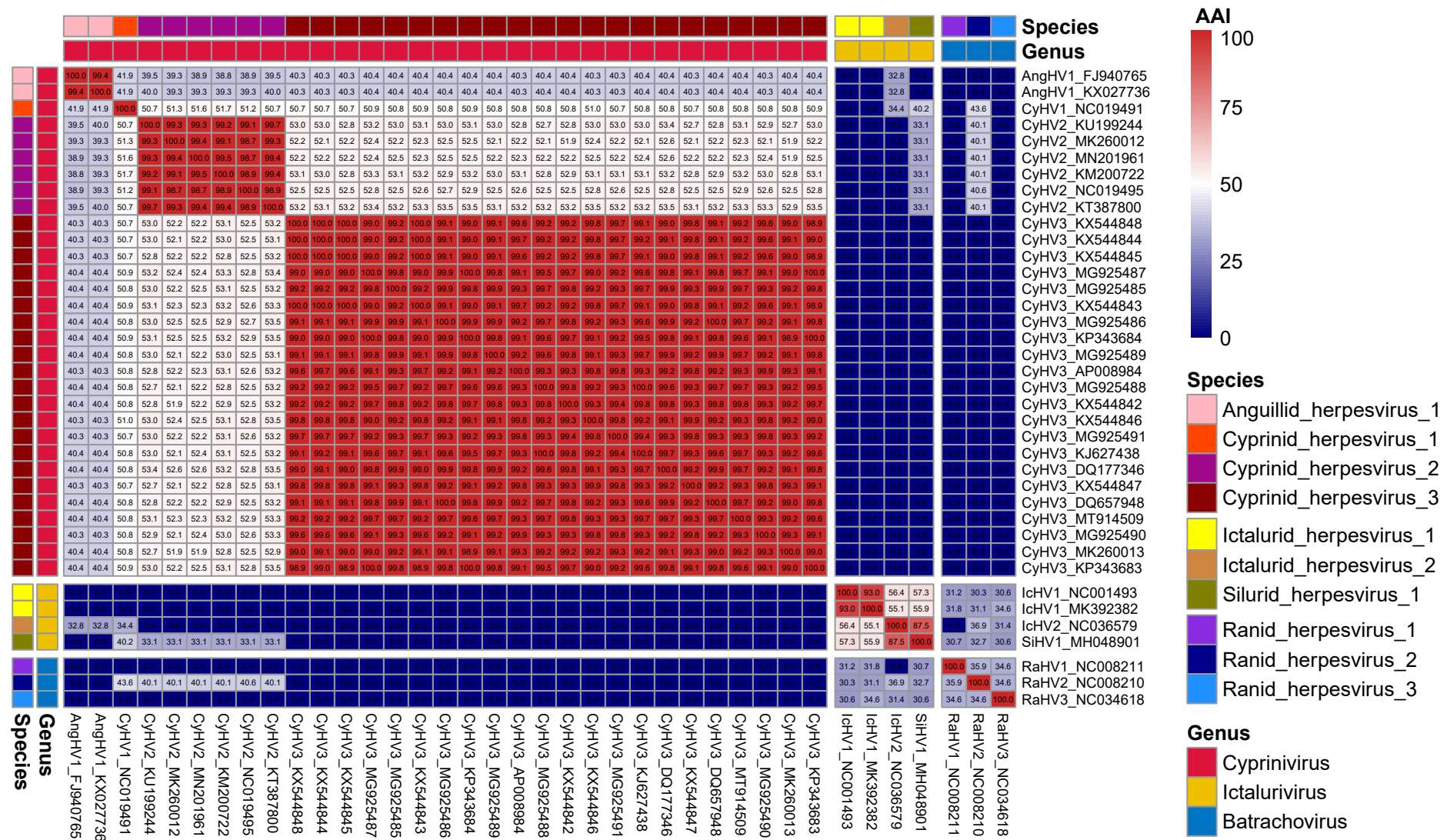

Figure S3 The heatmap showing pairwise AAI values between 38 alloherpesviruses

Supplement: Supplementary file 3 [file mmc3.pdf]
