# Supplementary material for: Comparative genomic analysis of alloherpesviruses: Exploring an available genus/species demarcation proposal and method
Source: Virus Res. 2023 Jul 26;334:199163. doi: 10.1016/j.virusres.2023.199163 (PMC10410580; doi:10.1016/j.virusres.2023.199163)
Supplement: Supplementary file 4 [file mmc4.pdf]

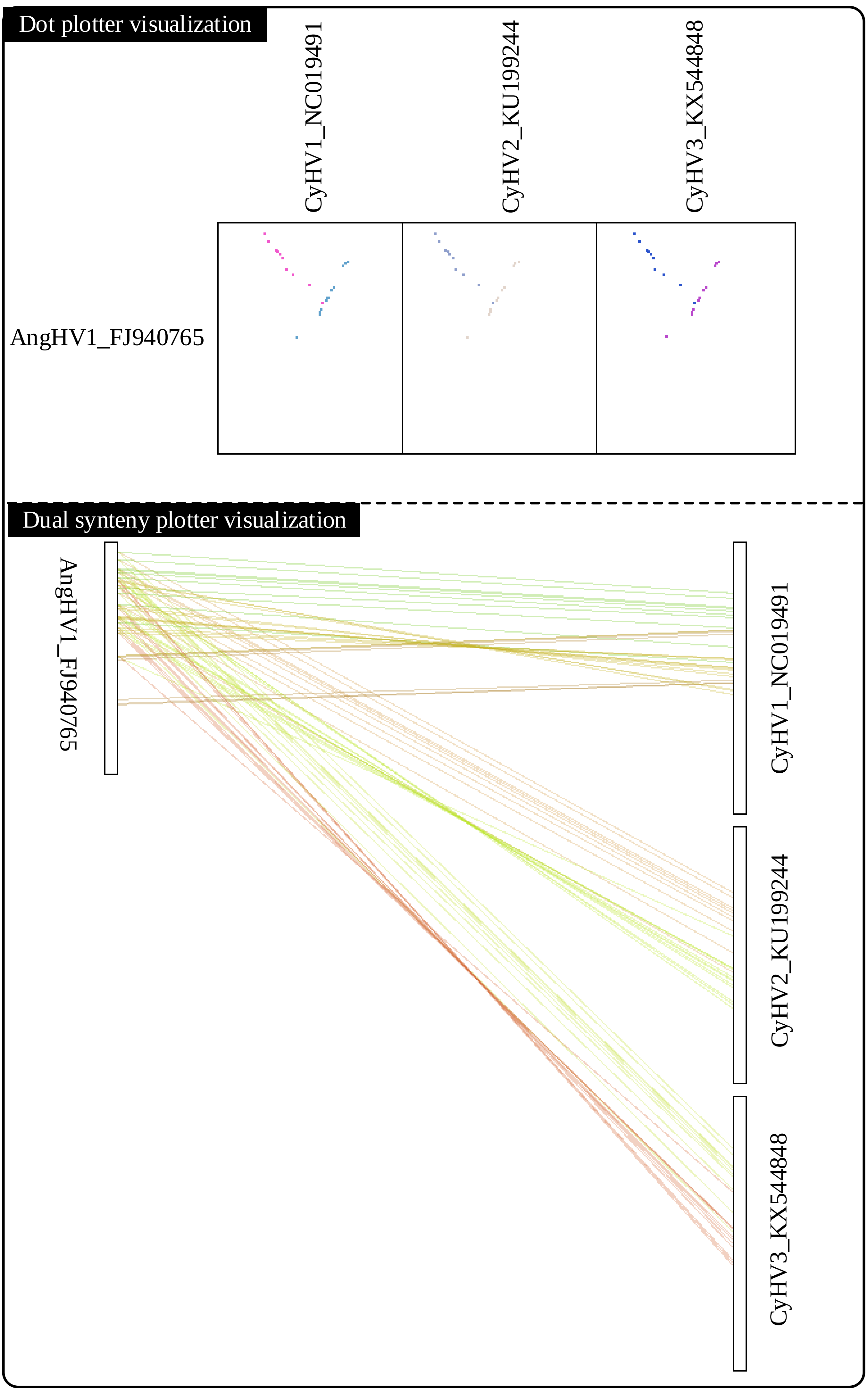

Figure S4 The the collinearity analysis between AngHV1 and CyHV1-3. Selecting one genome sequence from each species for the analysis.
