# Supplementary material for: Comparative genomic analysis of alloherpesviruses: Exploring an available genus/species demarcation proposal and method
Source: Virus Res. 2023 Jul 26;334:199163. doi: 10.1016/j.virusres.2023.199163 (PMC10410580; doi:10.1016/j.virusres.2023.199163)
Supplement: Supplementary file 5 [file mmc5.pdf]

Figure S5 The dot-plot showing the collinearity of 40 alloherpesviruses (Filter out different BLASTP pident values). Take Figure S5-1 as an example, filter pident values of all-versus-all BLASTP result before drawing dot-plots (less than 10% are removed). Thus line segments in the boxes of S5-1 represent ≥ 90% collinear/similarity fragments.

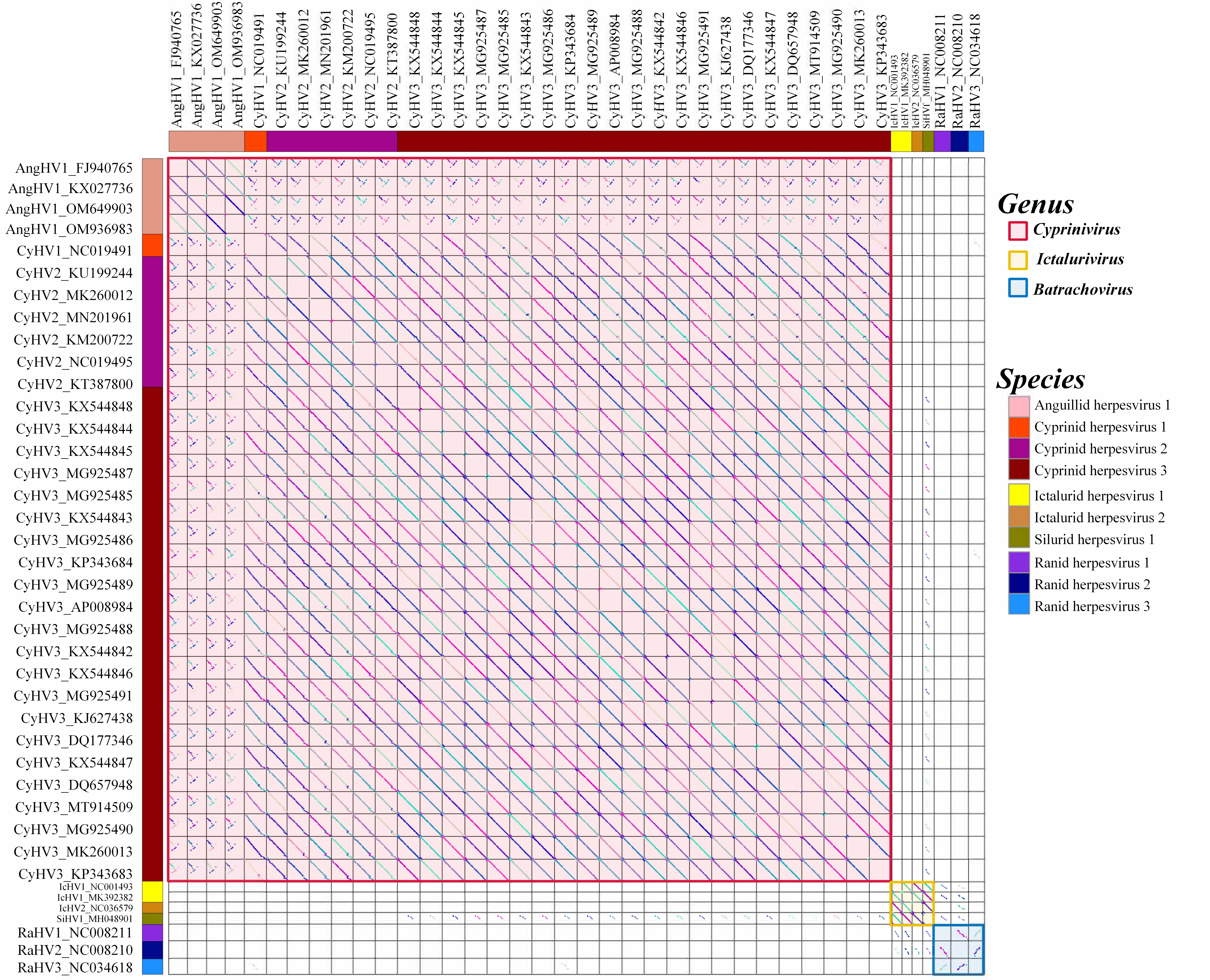

Figure S5-1: Filter out less than 10% identical matches

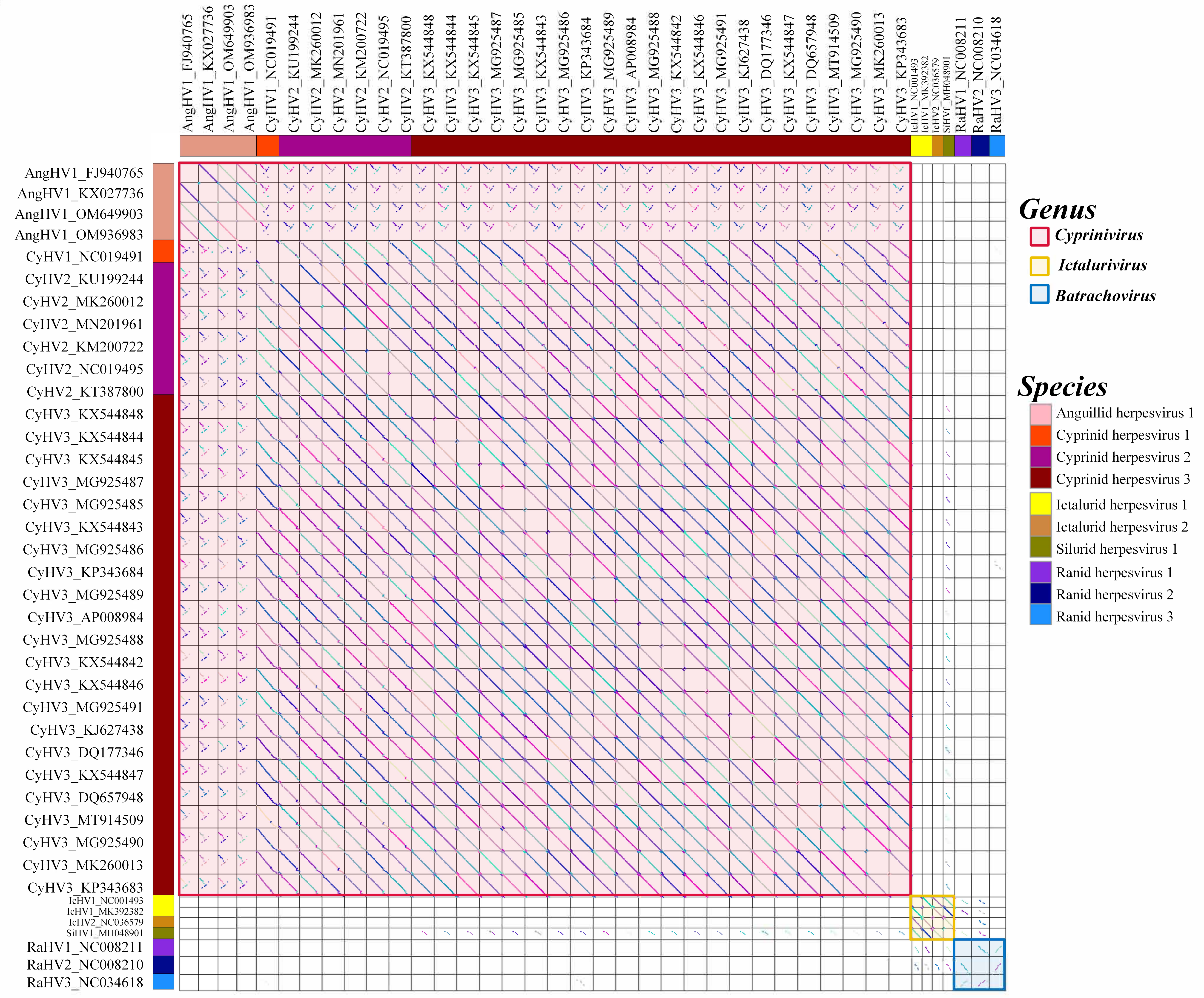

Figure S5-2: Filter out less than 20% identical matches

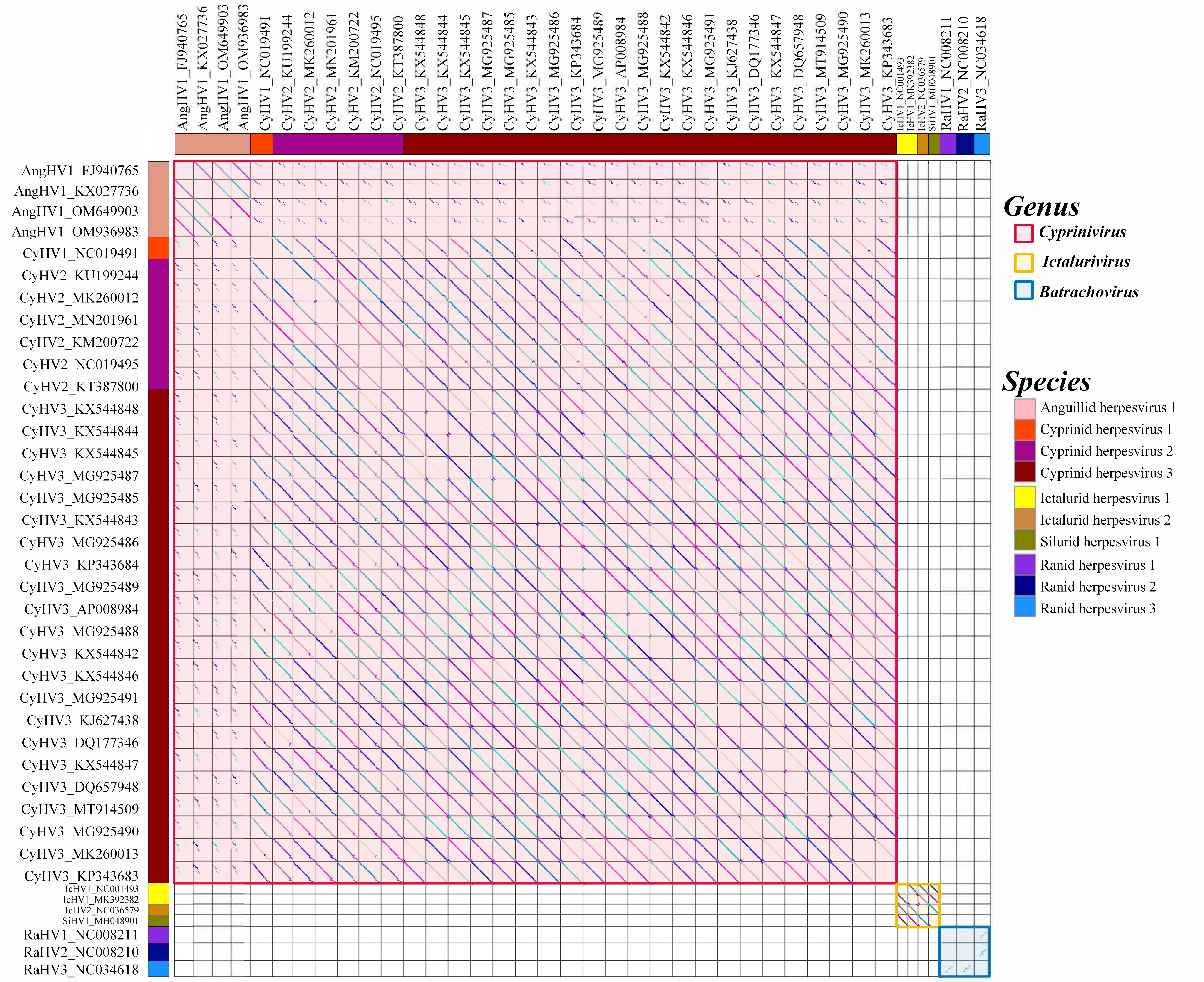

Figure S5-3: Filter out less than 30% identical matches

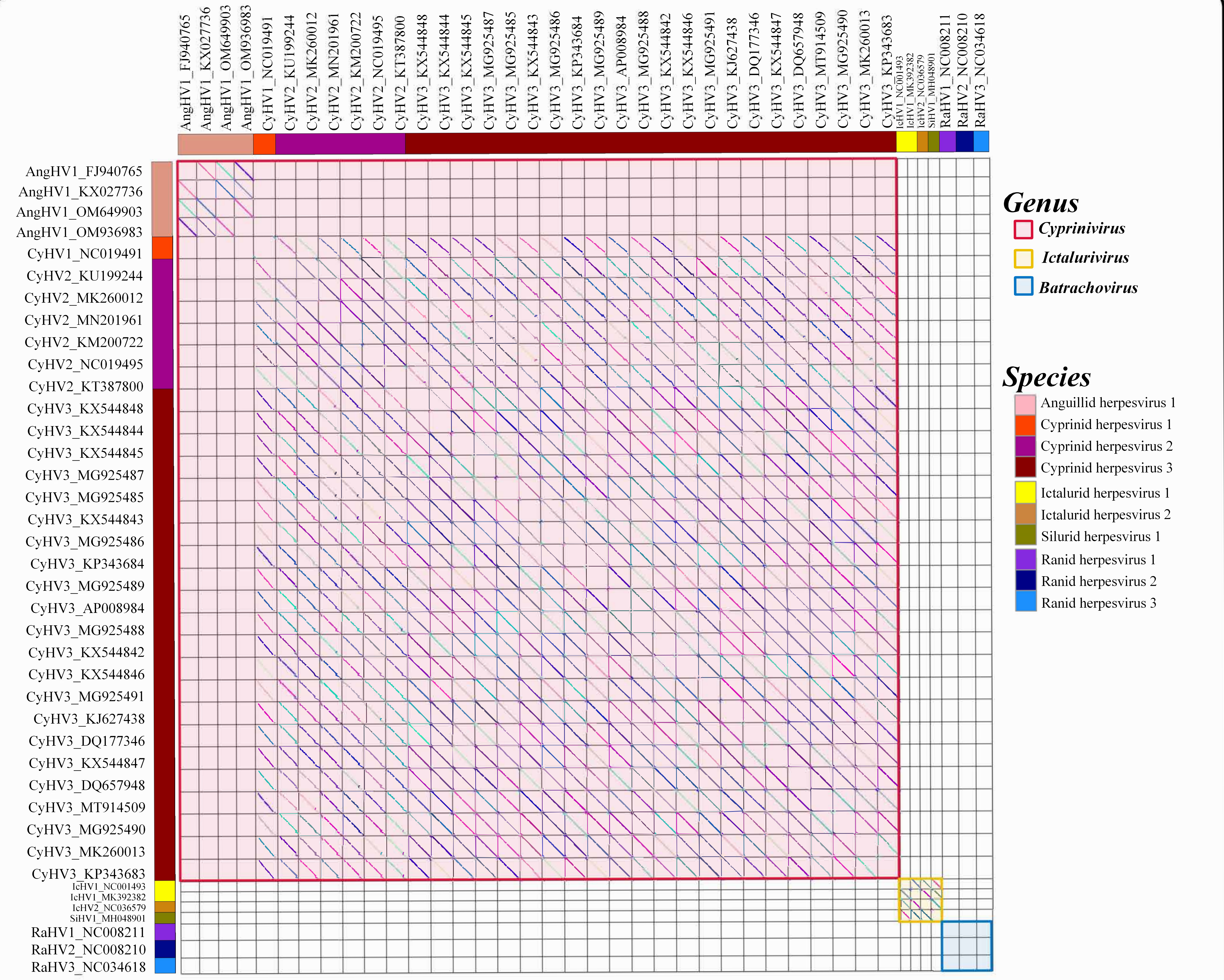

Figure S5-4: Filter out less than 40% identical matches

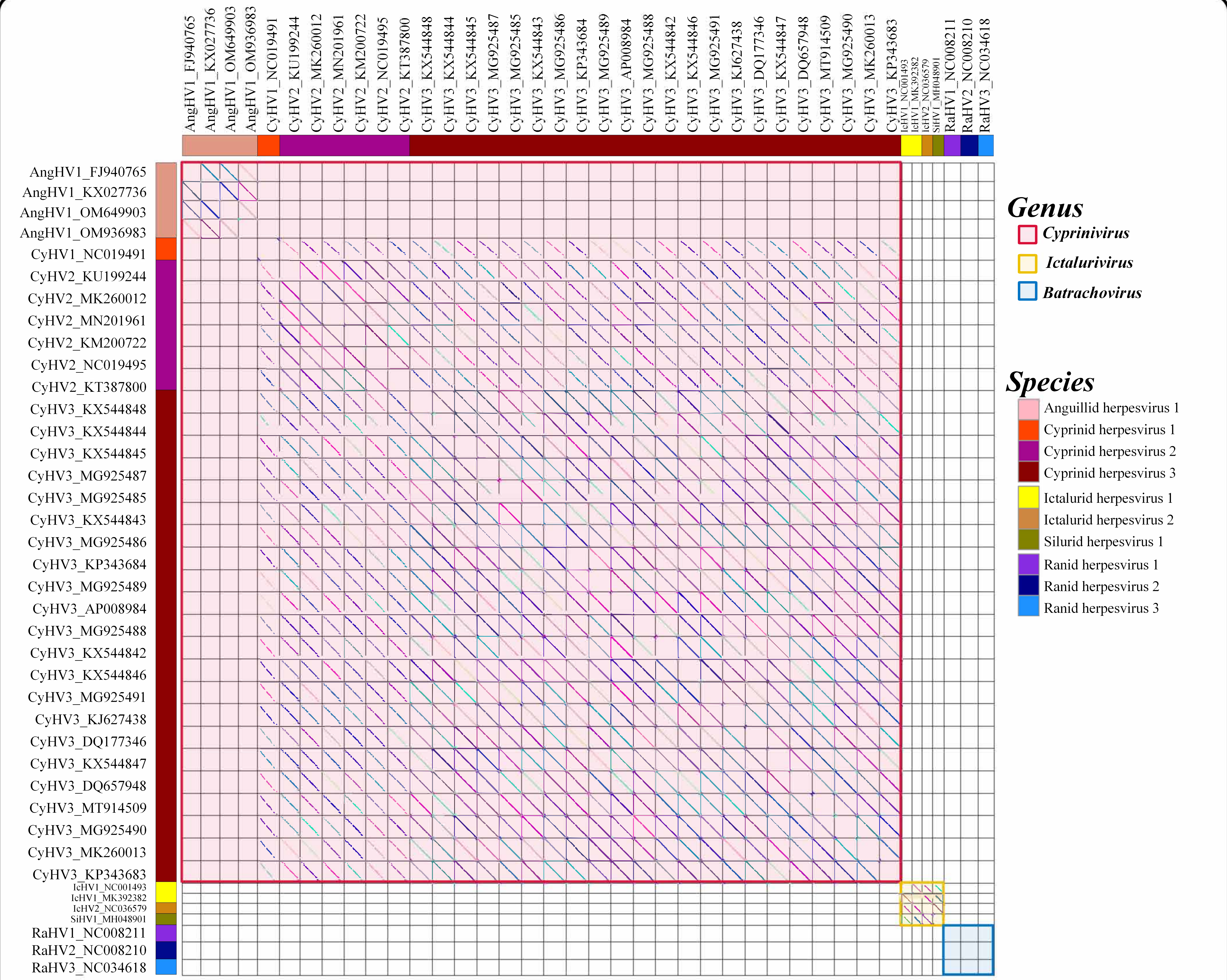

Figure S5-5: Filter out less than 50% identical matches

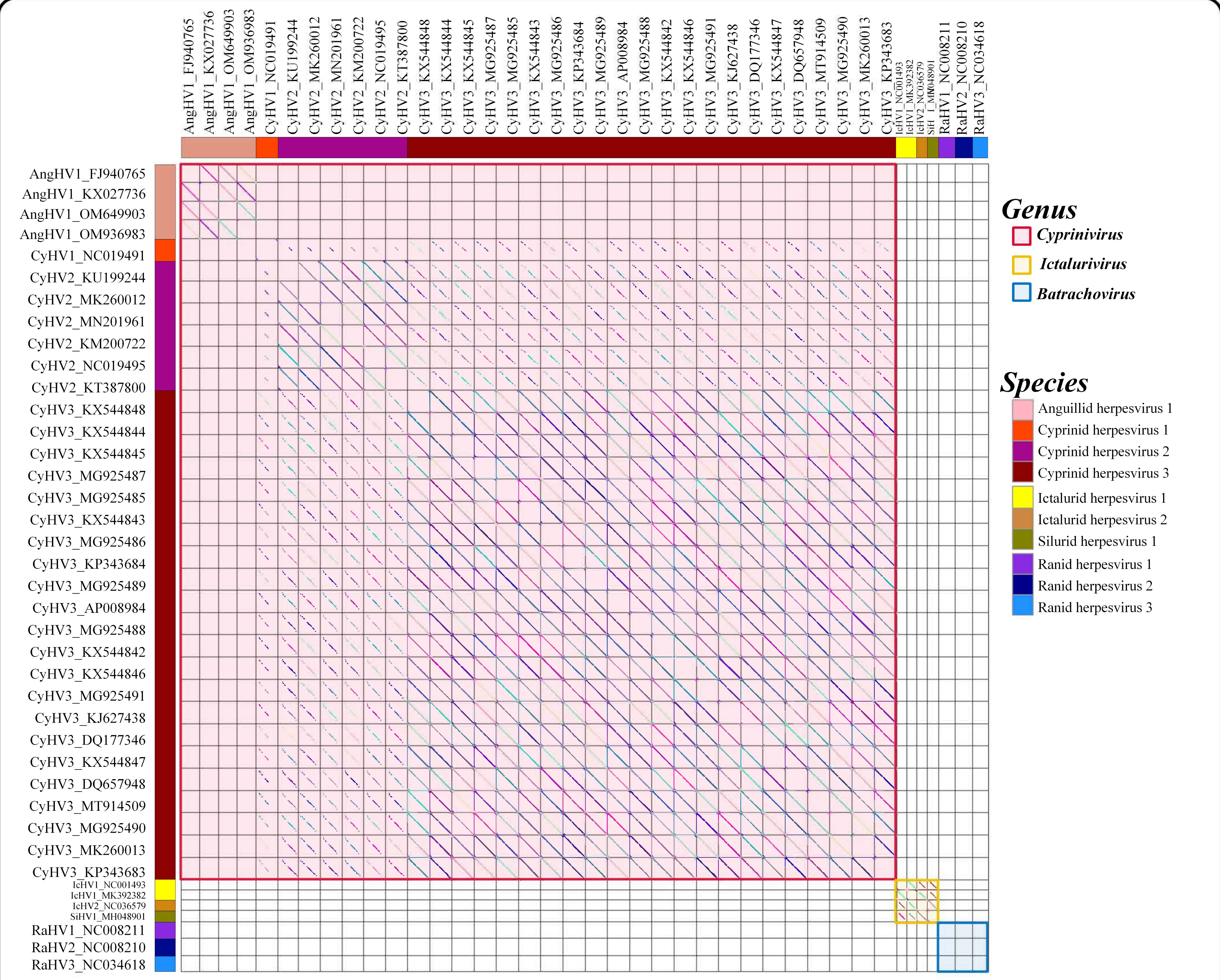

Figure S5-6: Filter out less than 60% identical matches

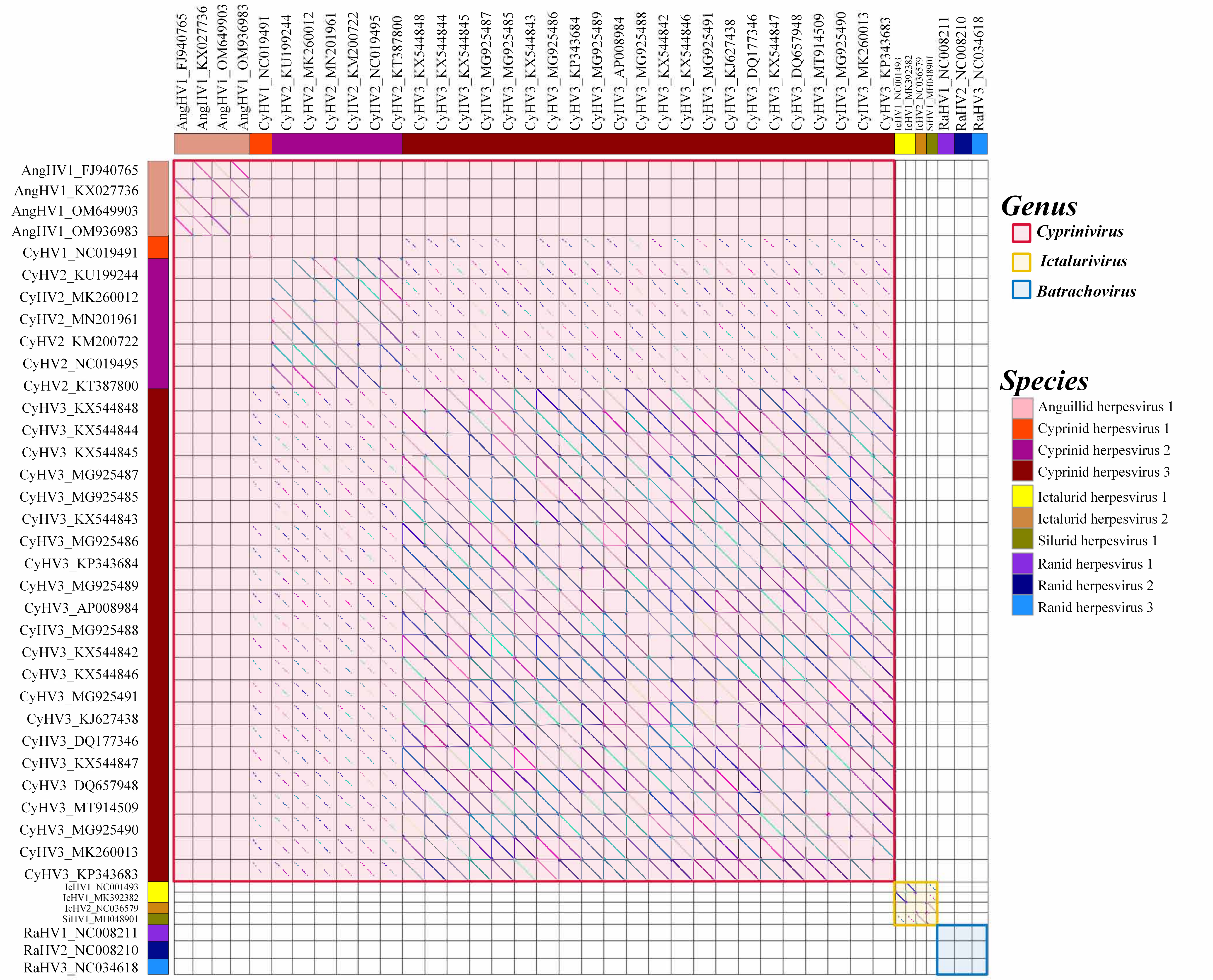

Figure S5-7: Filter out less than 70% identical matches

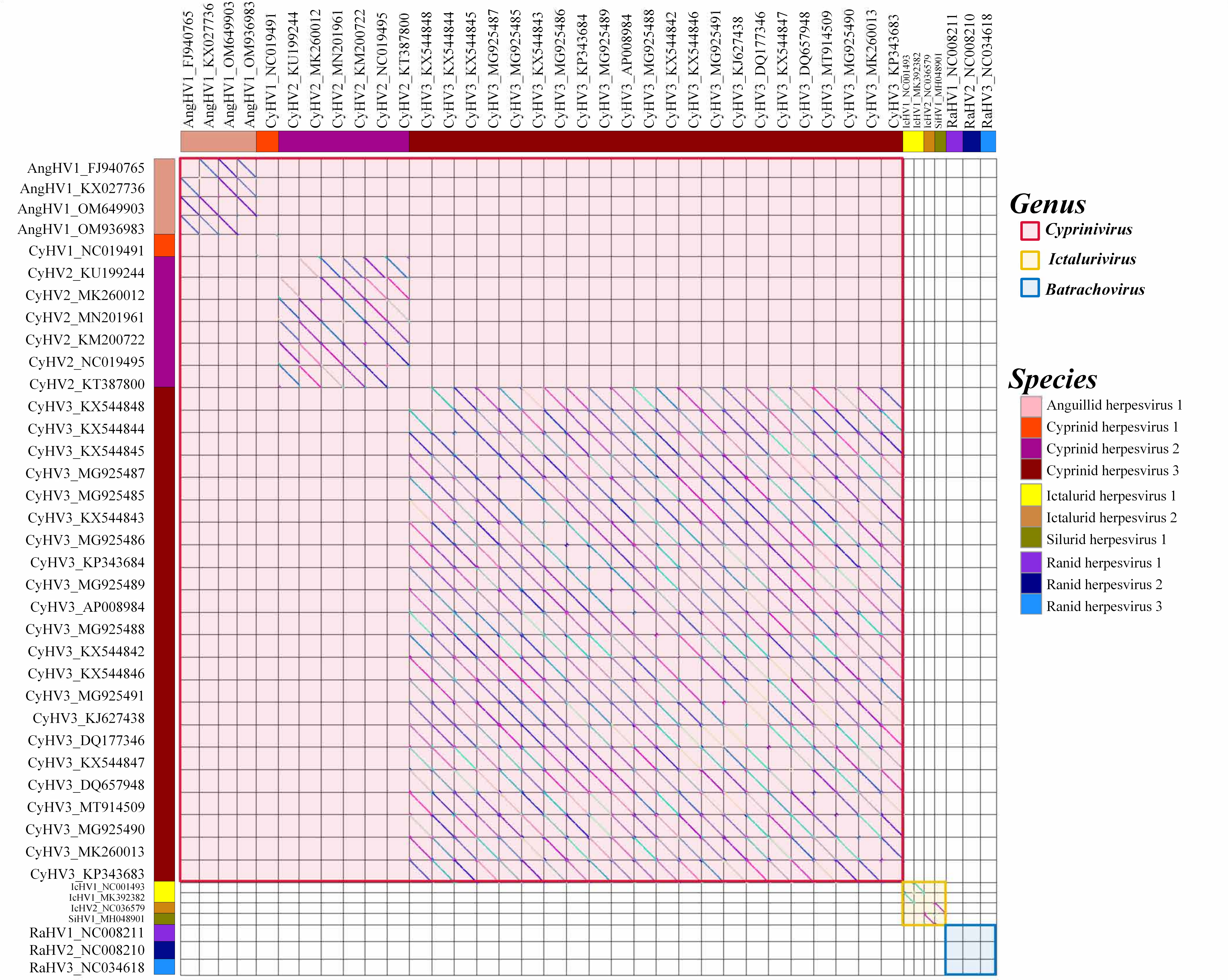

Figure S5-8: Filter out less than 80% identical matches

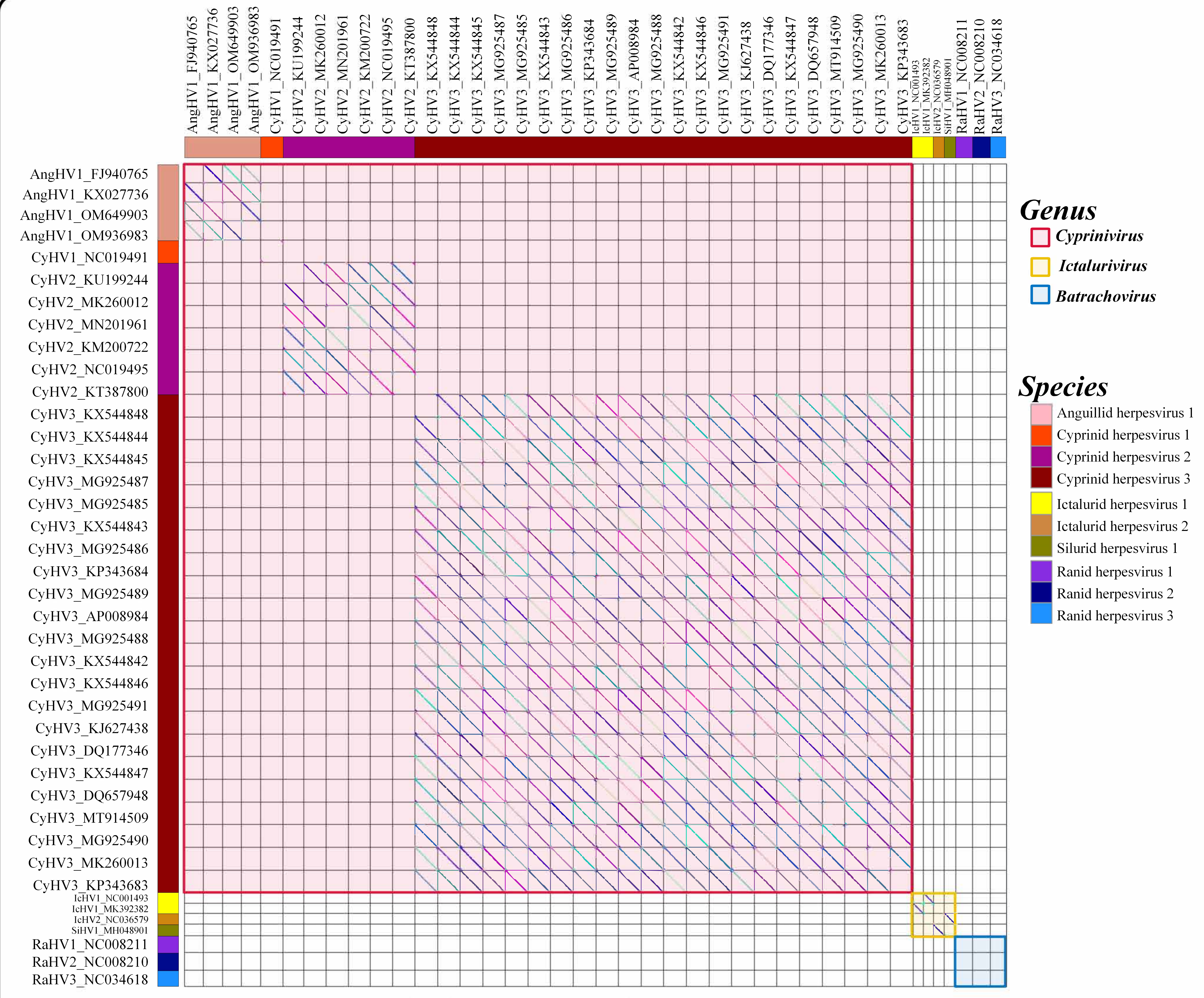

Figure S5-9: Filter out less than 90% identical matches

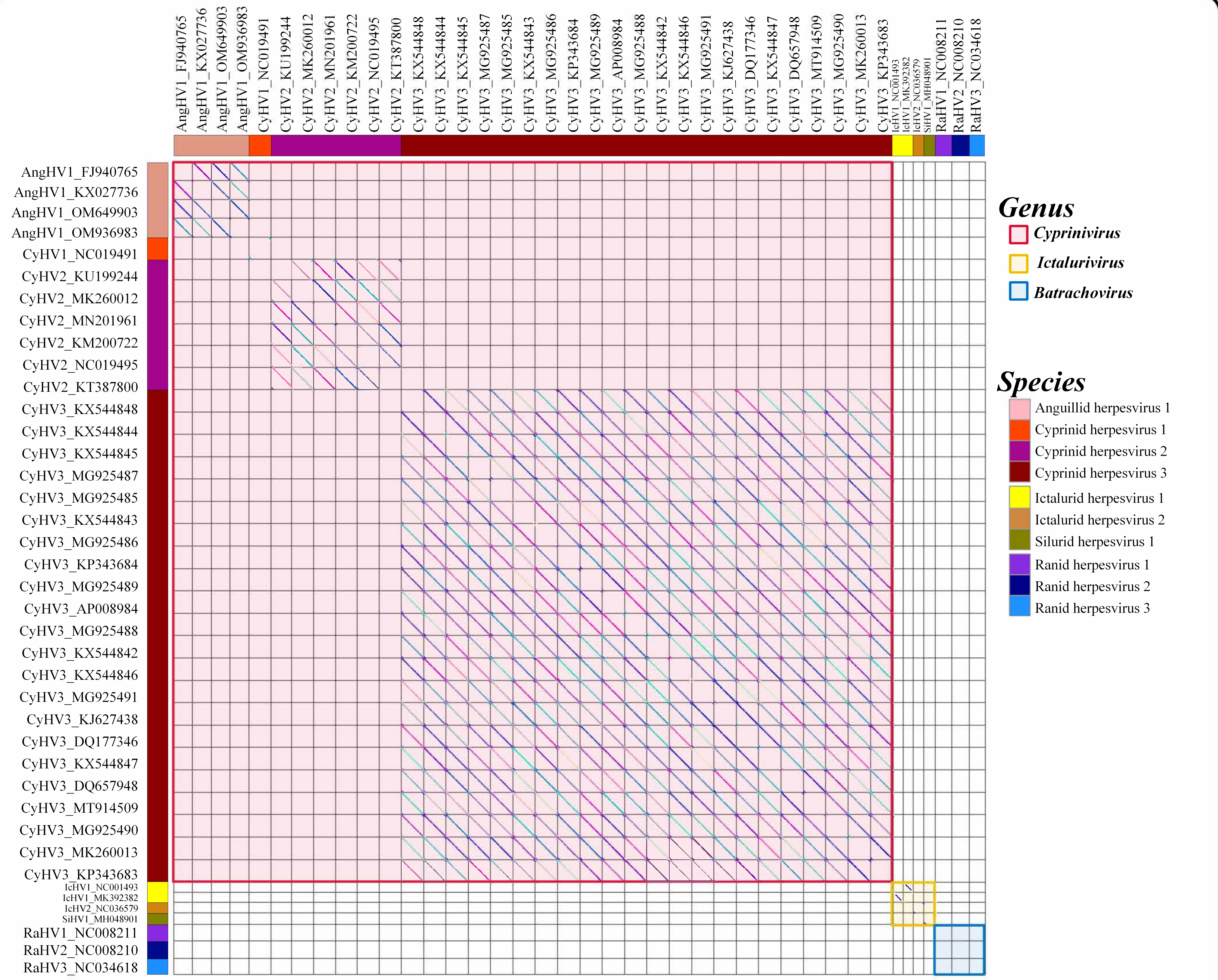

Figure S5-10: Filter out less than 98% identical matches
