# Supplementary material for: Comparative genomic analysis of alloherpesviruses: Exploring an available genus/species demarcation proposal and method
Source: Virus Res. 2023 Jul 26;334:199163. doi: 10.1016/j.virusres.2023.199163 (PMC10410580; doi:10.1016/j.virusres.2023.199163)

Figure S6 Construction of core-gene phylogenetic trees using different software (IQ-TREE/MEGA), sequence types (AA/NA sequences) and methods (ML/NJ). The number on the branch indicates the evolutionary distance. The color of the points between branches indicates Bootstrap. The shape of the endpoint indicates the genus level and the color indicates the species level.

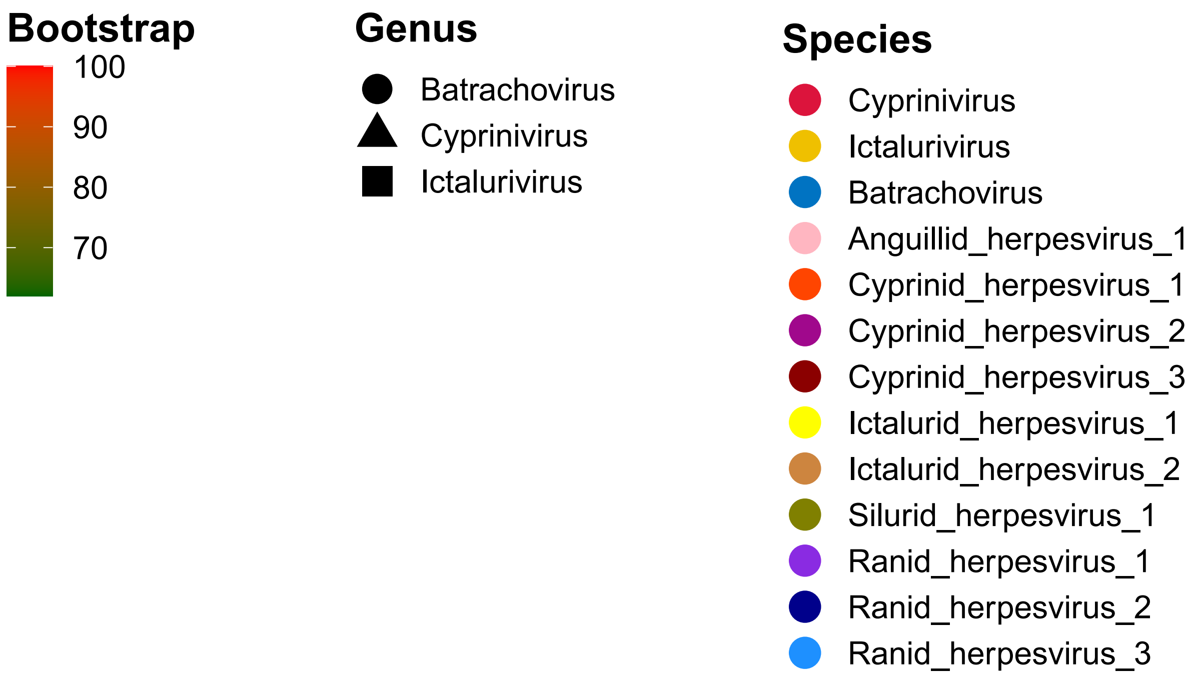

#6 AA: ML method using IQ−TREE

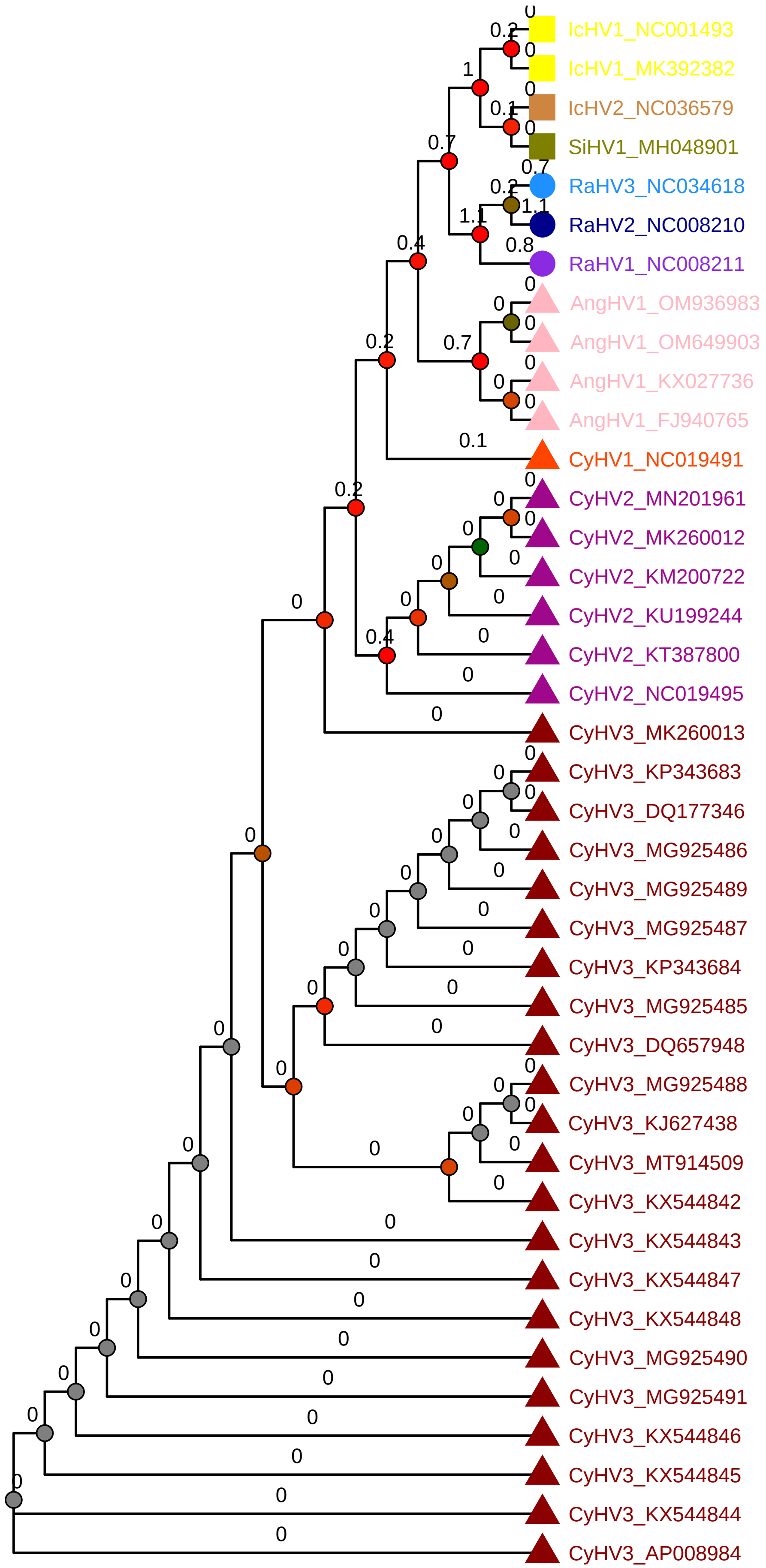

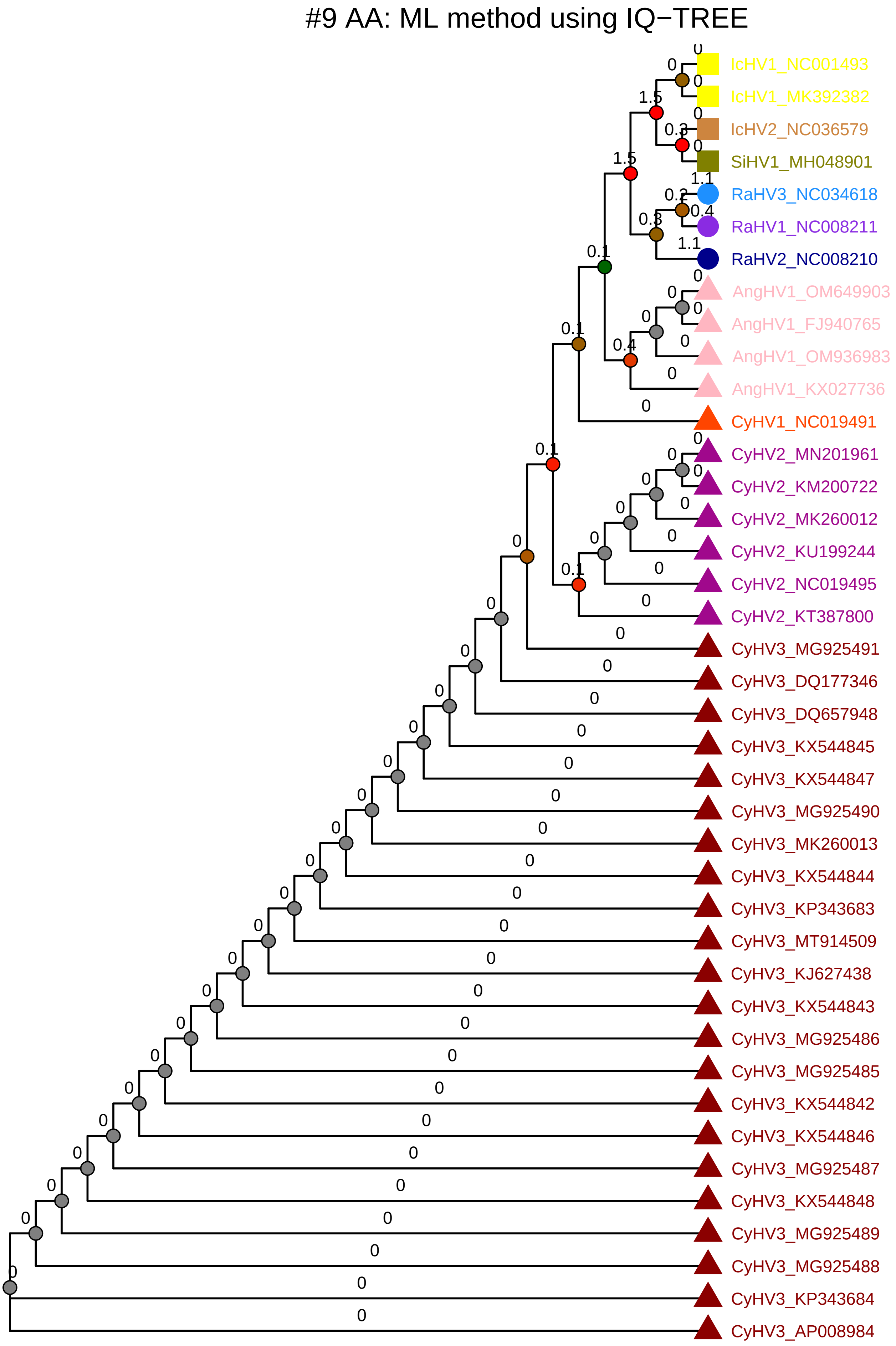

#9 AA: ML method using IQ−TREE
IcHV1_NC001493
IcHV1_MK392382
IcHV2_NC036579
SiHV1_MH048901
RaHV3_NC034618
RaHV1_NC008211
RaHV2_NC008210
AngHV1_OM649903
AngHV1_FJ940765
AngHV1_OM936983
AngHV1_KX027736
CyHV1_NC019491
CyHV2_MN201961
CyHV2_KM200722
CyHV2_MK260012
CyHV2_KU199244
CyHV2_NC019495
CyHV2_KT387800
CyHV3_MG925491
CyHV3_DQ177346
CyHV3_DQ657948
CyHV3_KX544845
CyHV3_KX544847
CyHV3_MG925490
CyHV3_MK260013
CyHV3_KX544844
CyHV3_KP343683
CyHV3_MT914509
CyHV3_KJ627438
CyHV3_KX544843
CyHV3_MG925486
CyHV3_MG925485
CyHV3_KX544842
CyHV3_KX544846
CyHV3_MG925487
CyHV3_KX544848
CyHV3_MG925489
CyHV3_MG925488
CyHV3_KP343684
CyHV3_AP008984

#12 AA: ML method using IQ−TREE

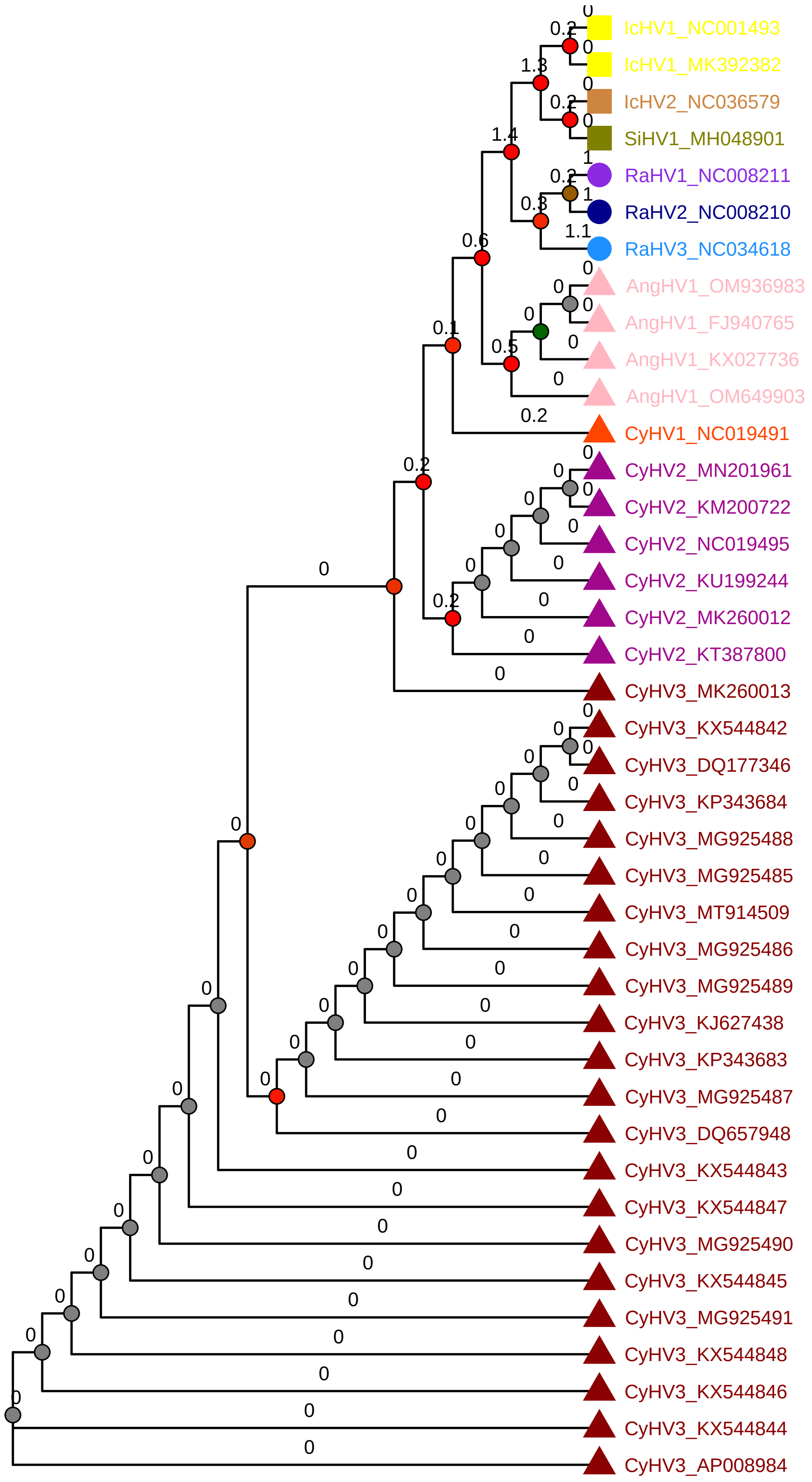

#13 AA: ML method using IQ−TREE

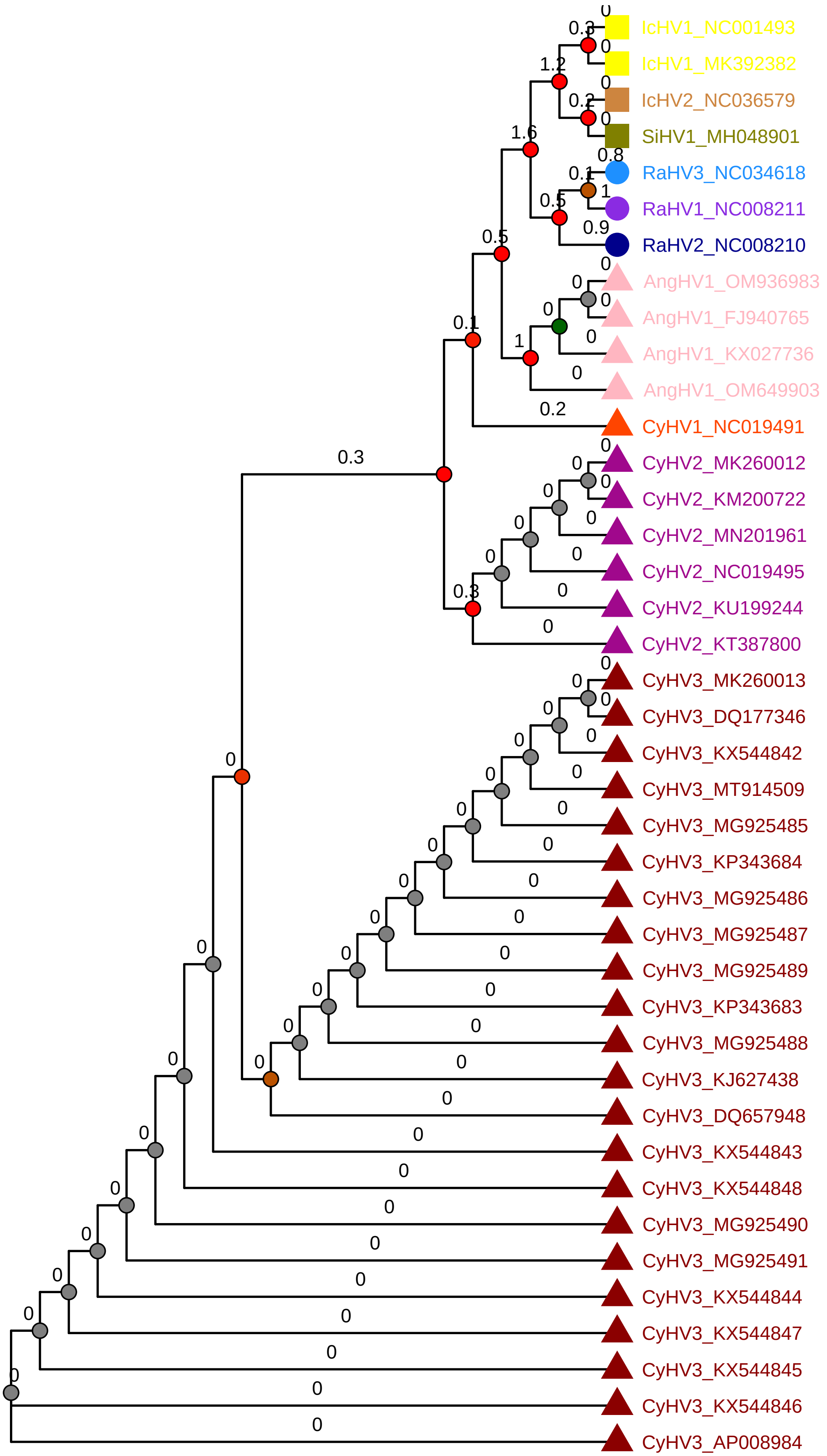

#26 AA: ML method using IQ−TREE

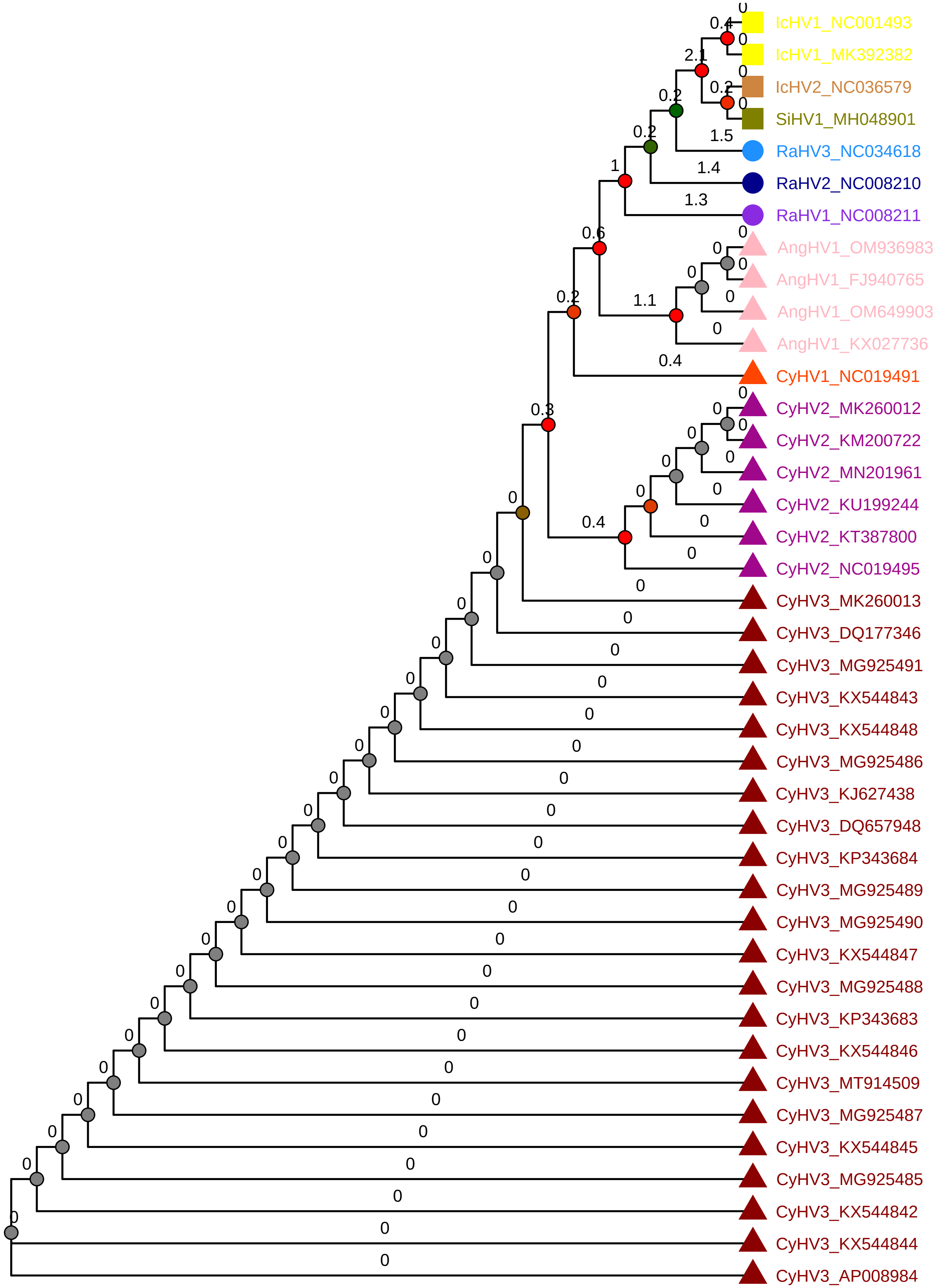

#27 AA: ML method using IQ−TREE

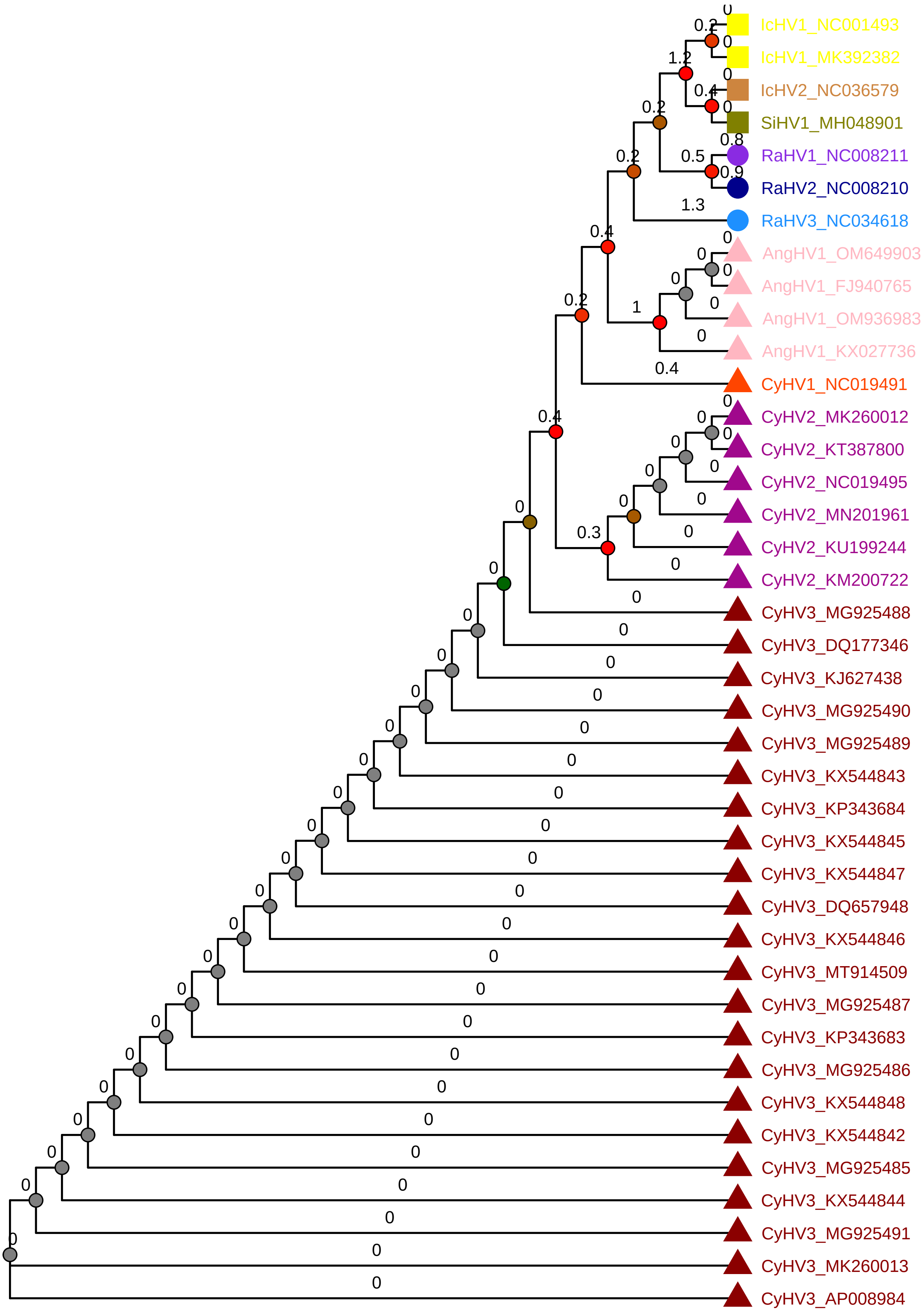

#28 AA: ML method using IQ−TREE

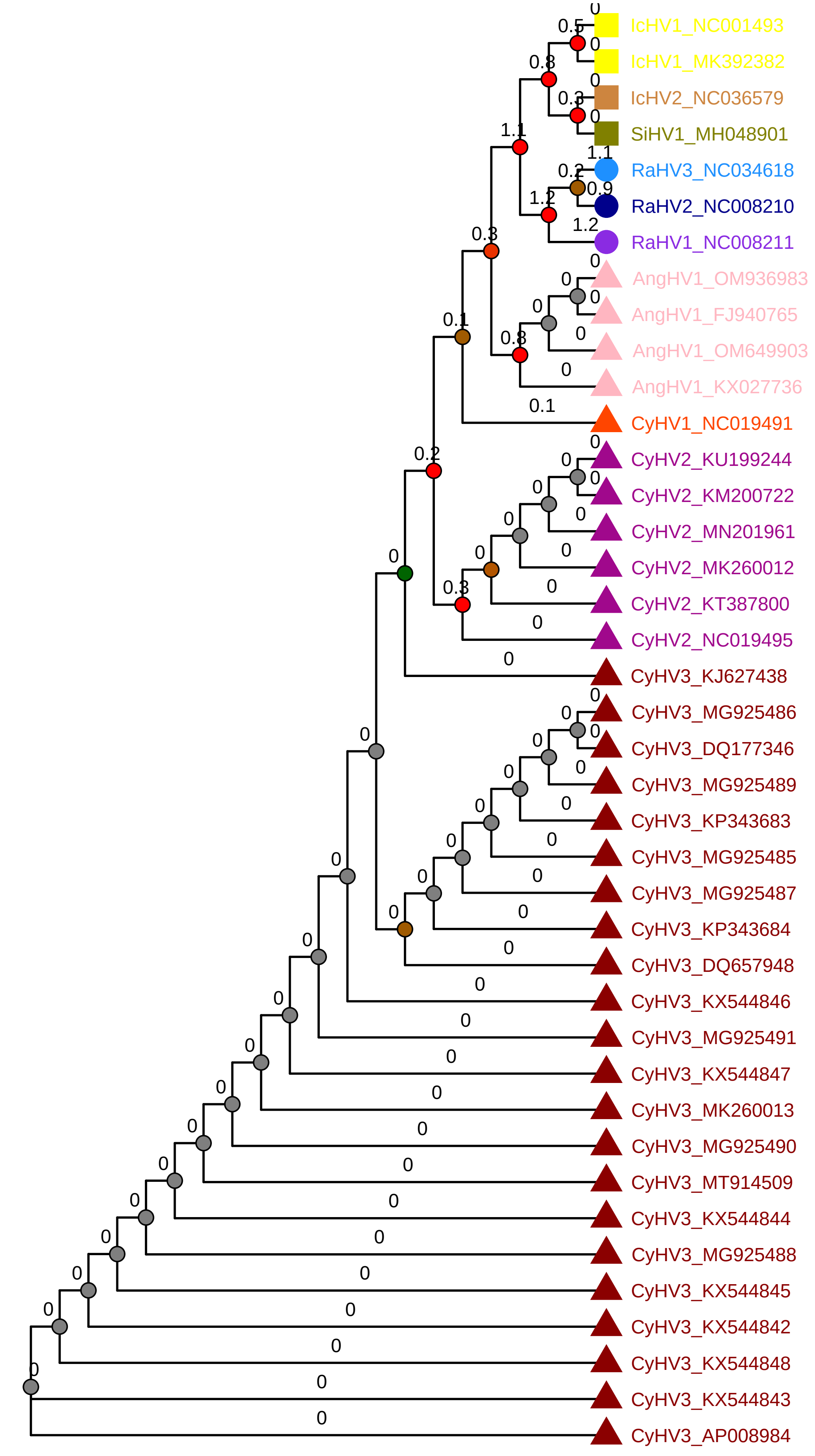

#29 AA: ML method using IQ−TREE

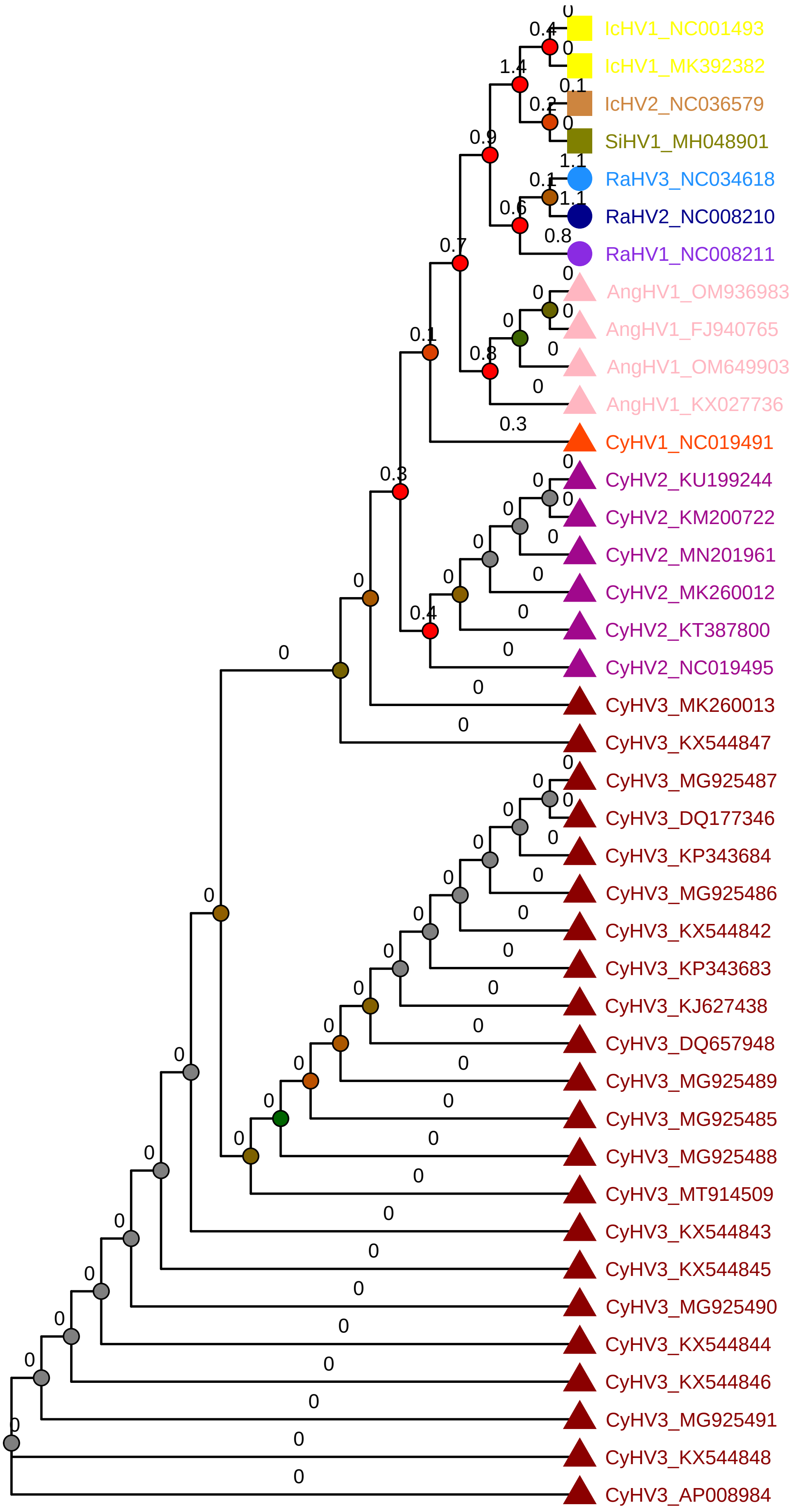

#30 AA: ML method using IQ−TREE

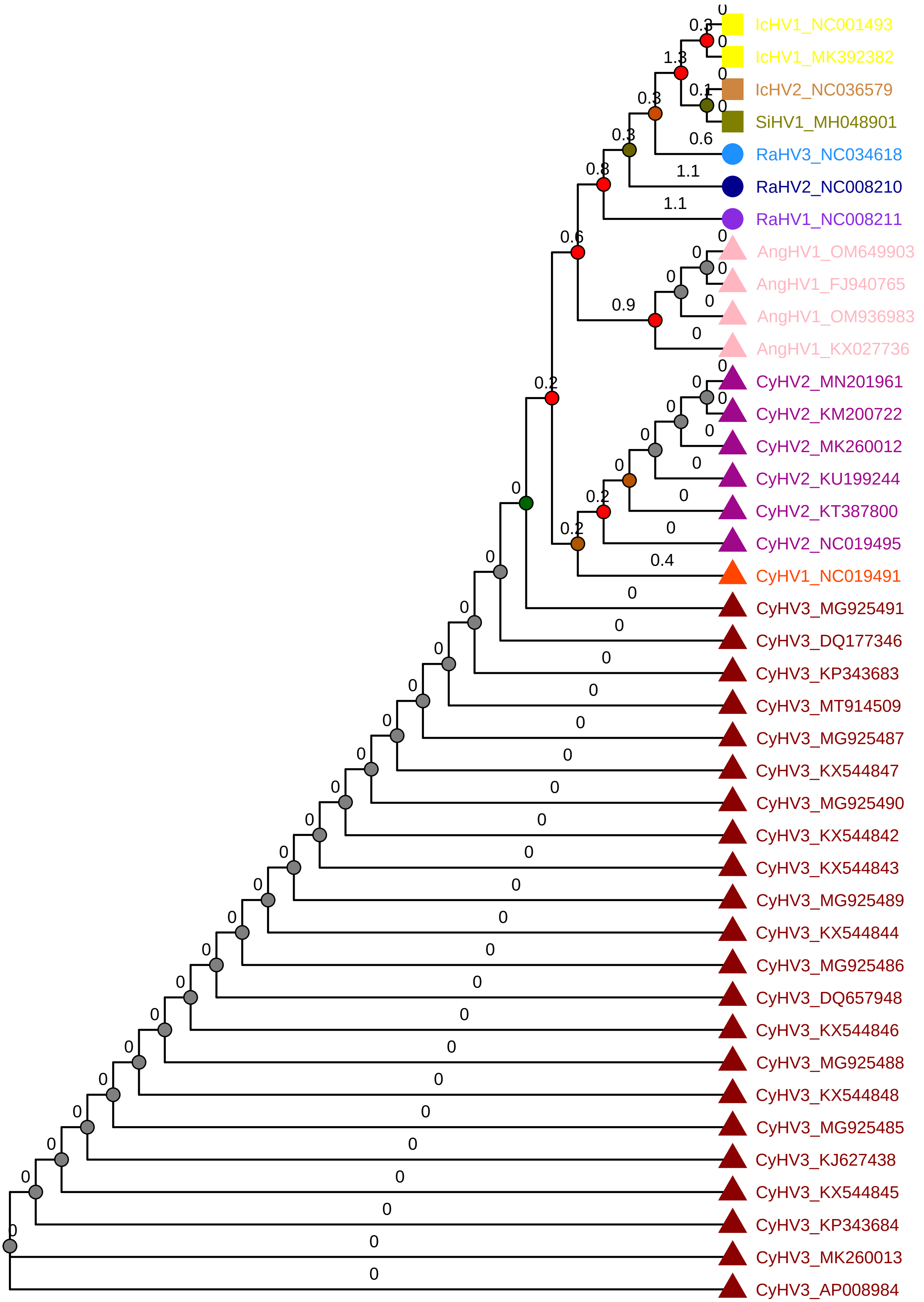

#31 AA: ML method using IQ−TREE

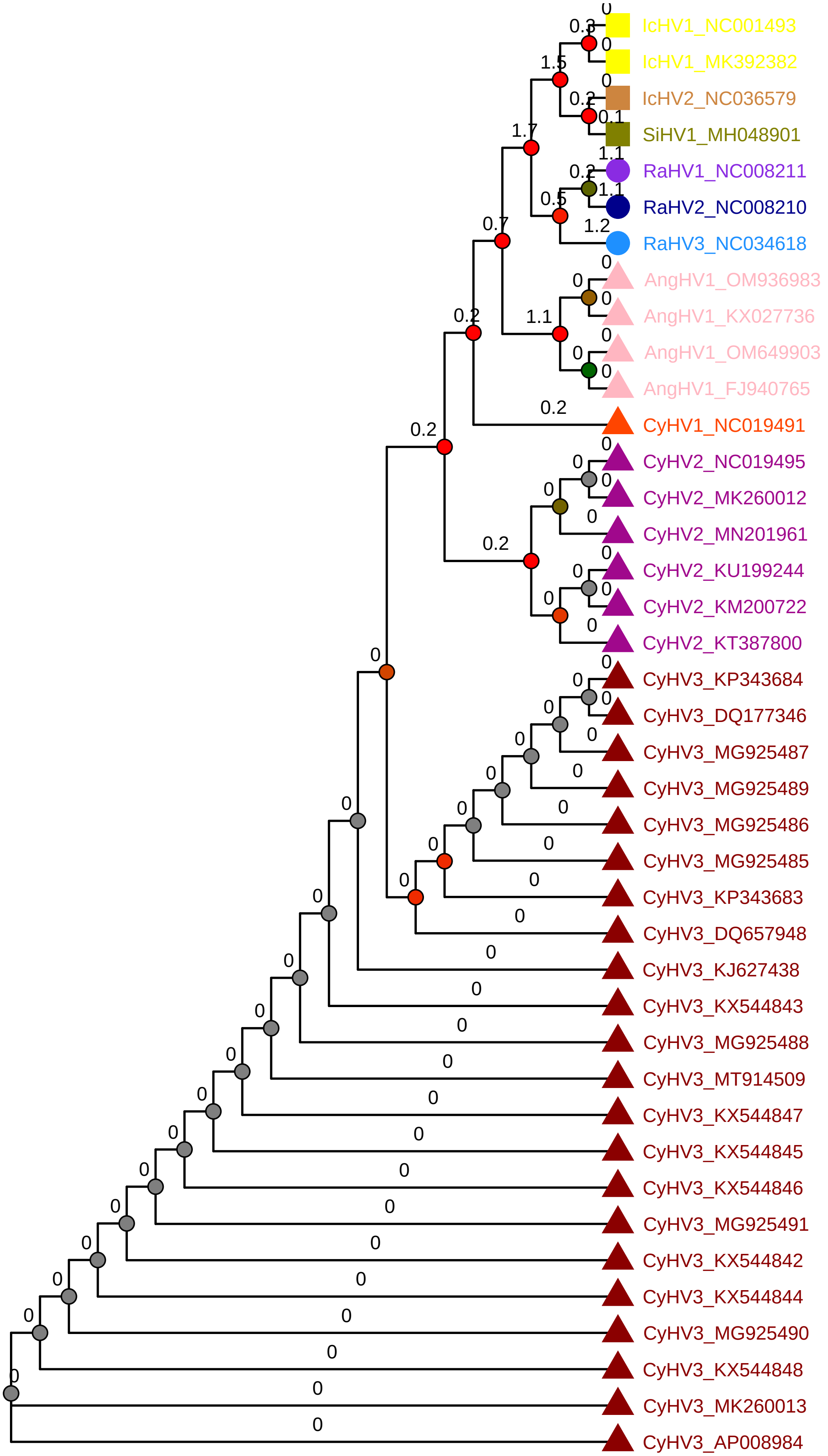

#32 AA: ML method using IQ−TREE

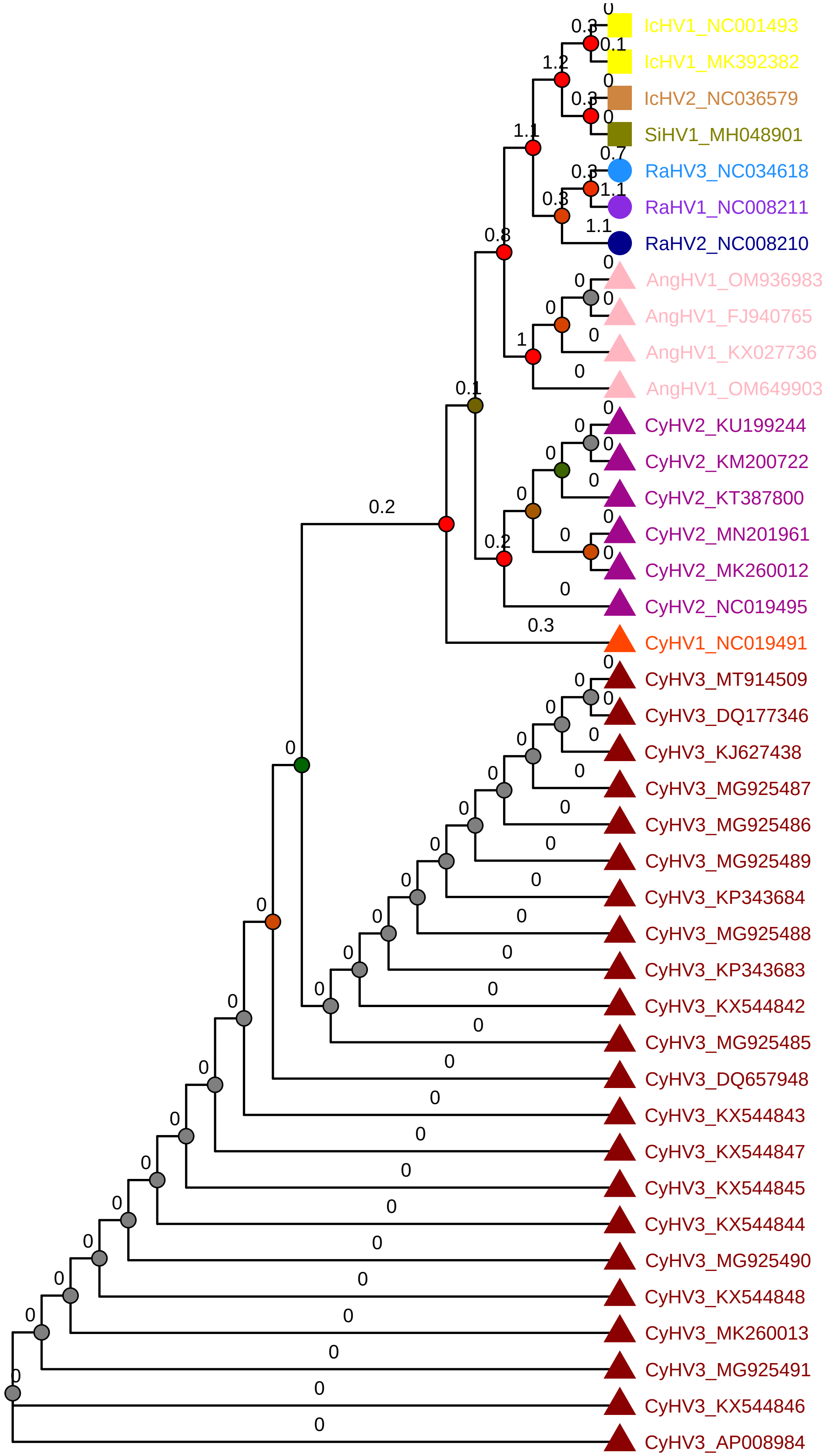

#6 NA: ML method using IQ−TREE

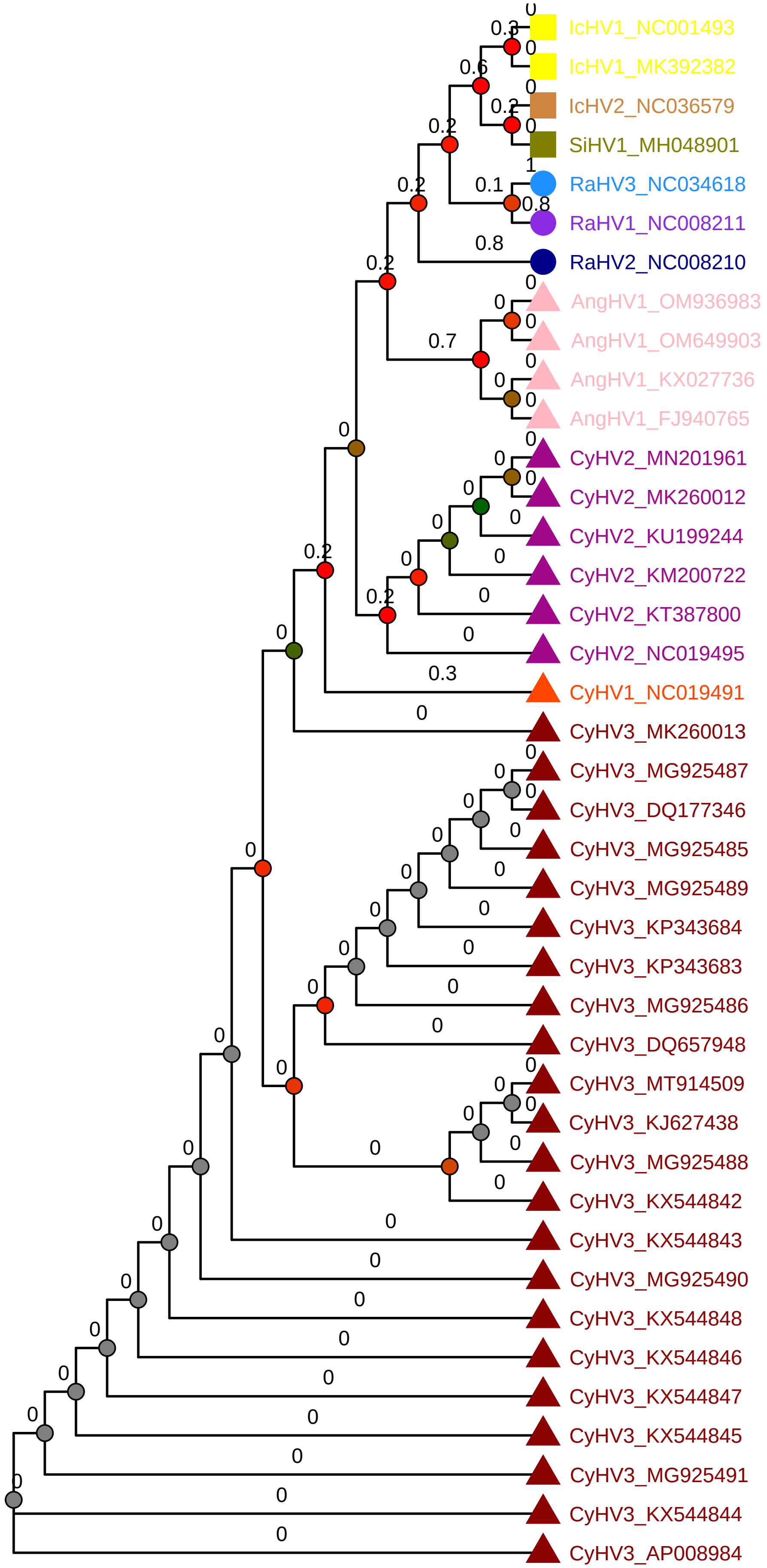

#9 NA: ML method using IQ−TREE

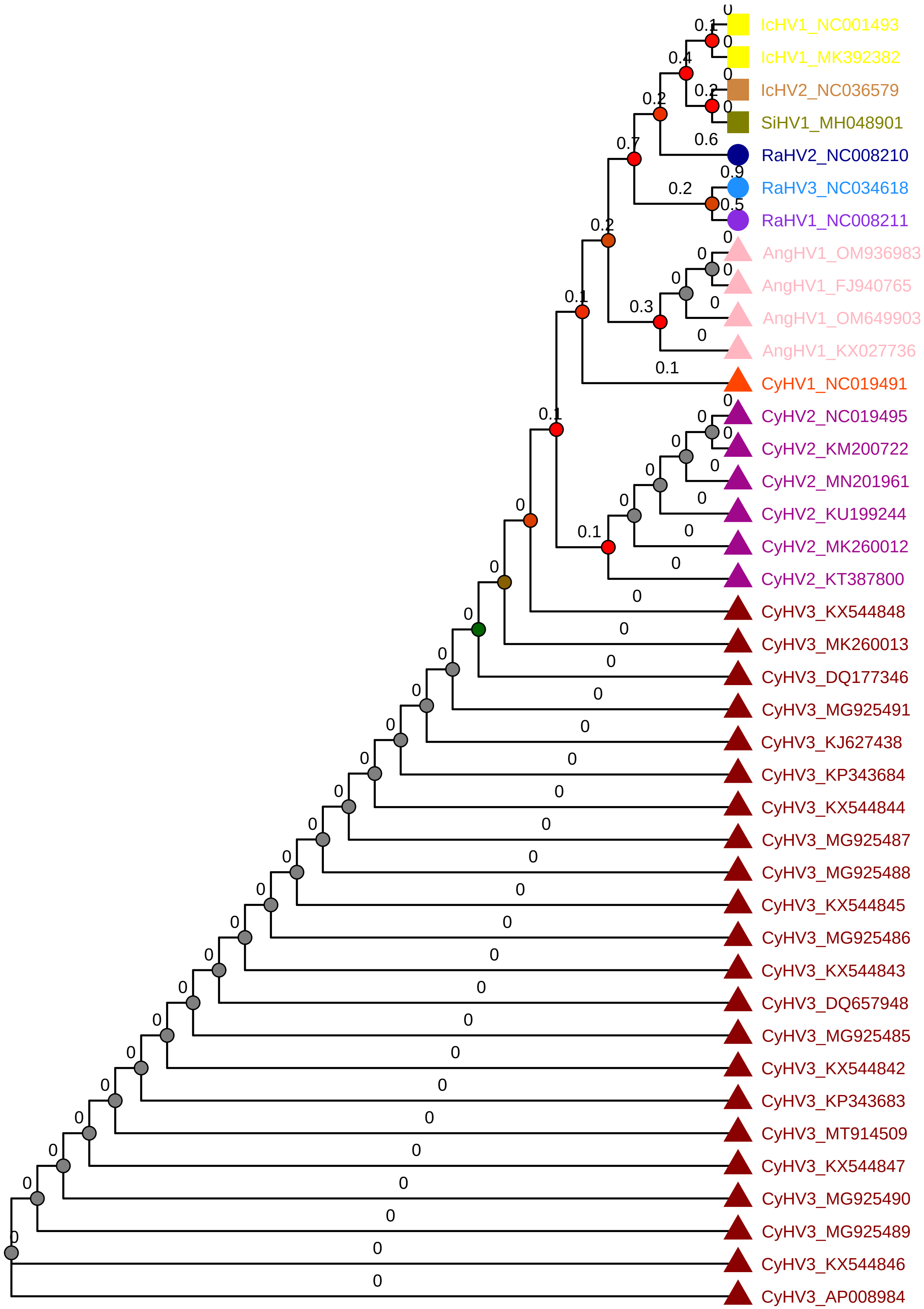

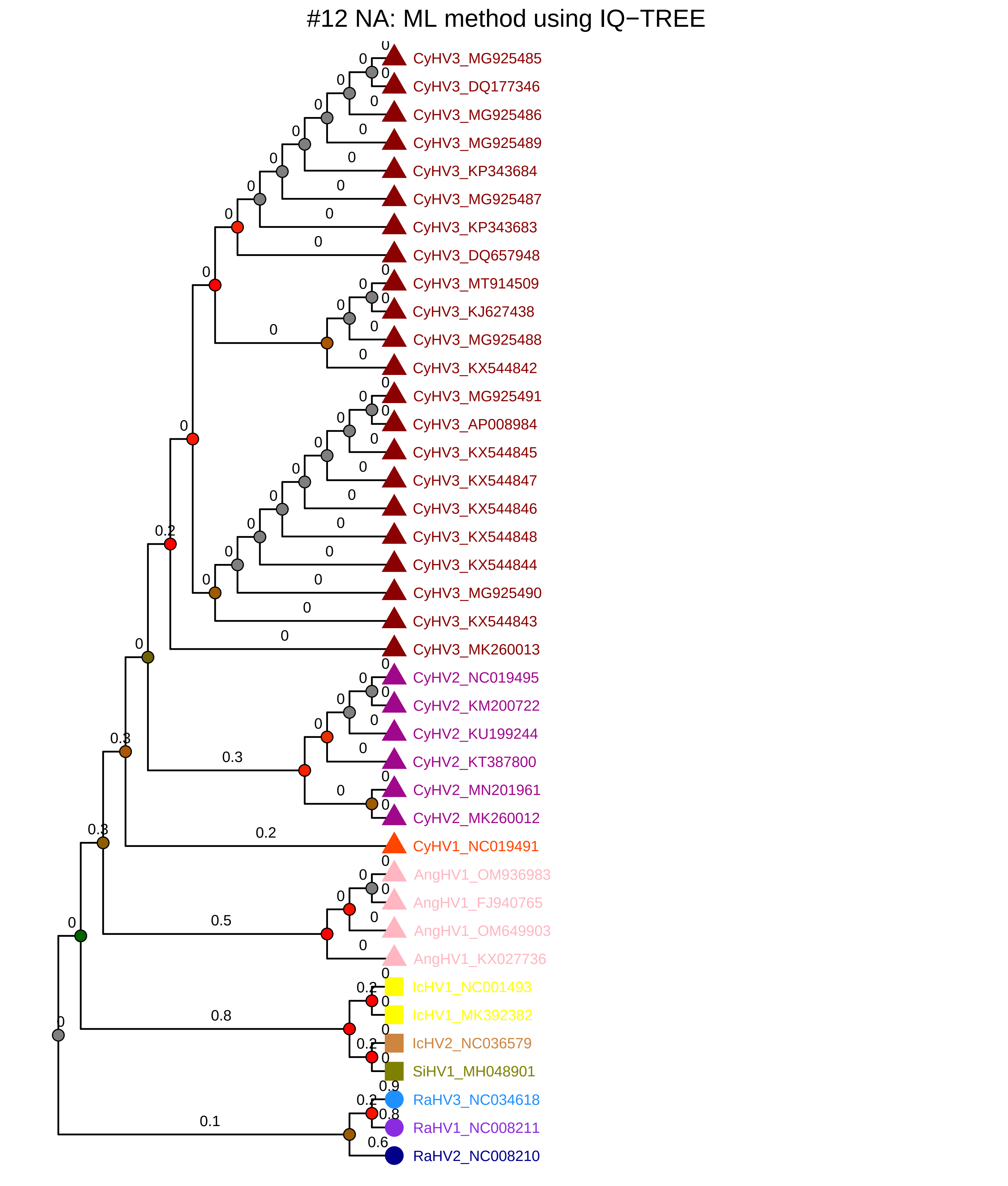
#12 NA: ML method using IQ−TREE
CyHV3_MG925485
CyHV3_DQ177346
CyHV3_MG925486
CyHV3_MG925489
CyHV3_KP343684
CyHV3_MG925487
CyHV3_KP343683
CyHV3_DQ657948
CyHV3_MT914509
CyHV3_KJ627438
CyHV3_MG925488
CyHV3_KX544842
CyHV3_MG925491
CyHV3_AP008984
CyHV3_KX544845
CyHV3_KX544847
CyHV3_KX544846
CyHV3_KX544848
CyHV3_KX544844
CyHV3_MG925490
CyHV3_KX544843
CyHV3_MK260013
CyHV2_NC019495
CyHV2_KM200722
CyHV2_KU199244
CyHV2_KT387800
CyHV2_MN201961
CyHV2_MK260012
CyHV1_NC019491
AngHV1_OM936983
AngHV1_FJ940765
AngHV1_OM649903
AngHV1_KX027736
IcHV1_NC001493
IcHV1_MK392382
IcHV2_NC036579
SiHV1_MH048901
RaHV3_NC034618
RaHV1_NC008211
RaHV2_NC008210
0.2
0.3
0.3
0.3
0.2
0.5
0.8
0.1
0.9
0.8
0.6

#13 NA: ML method using IQ−TREE

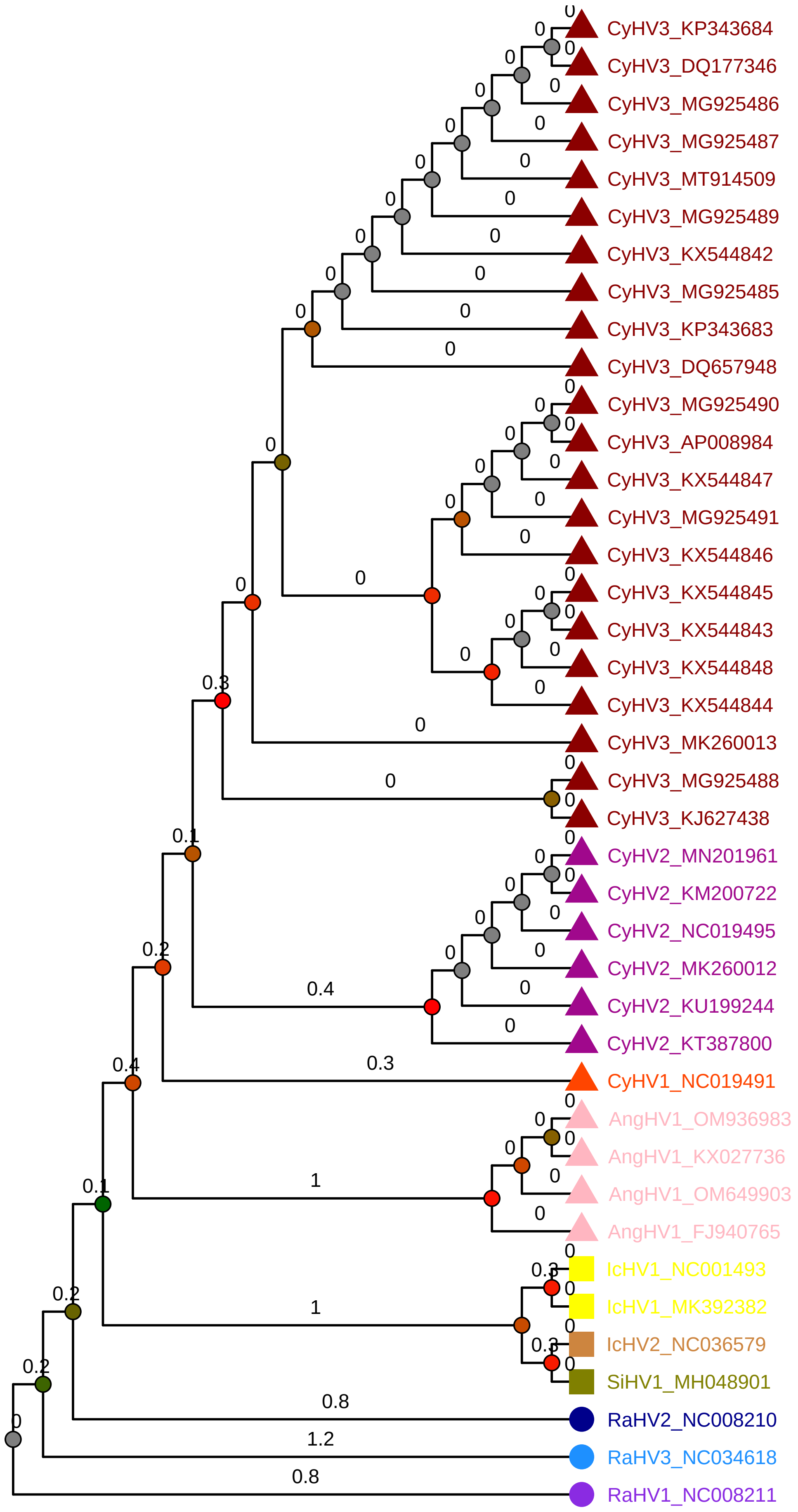

#26 NA: ML method using IQ−TREE

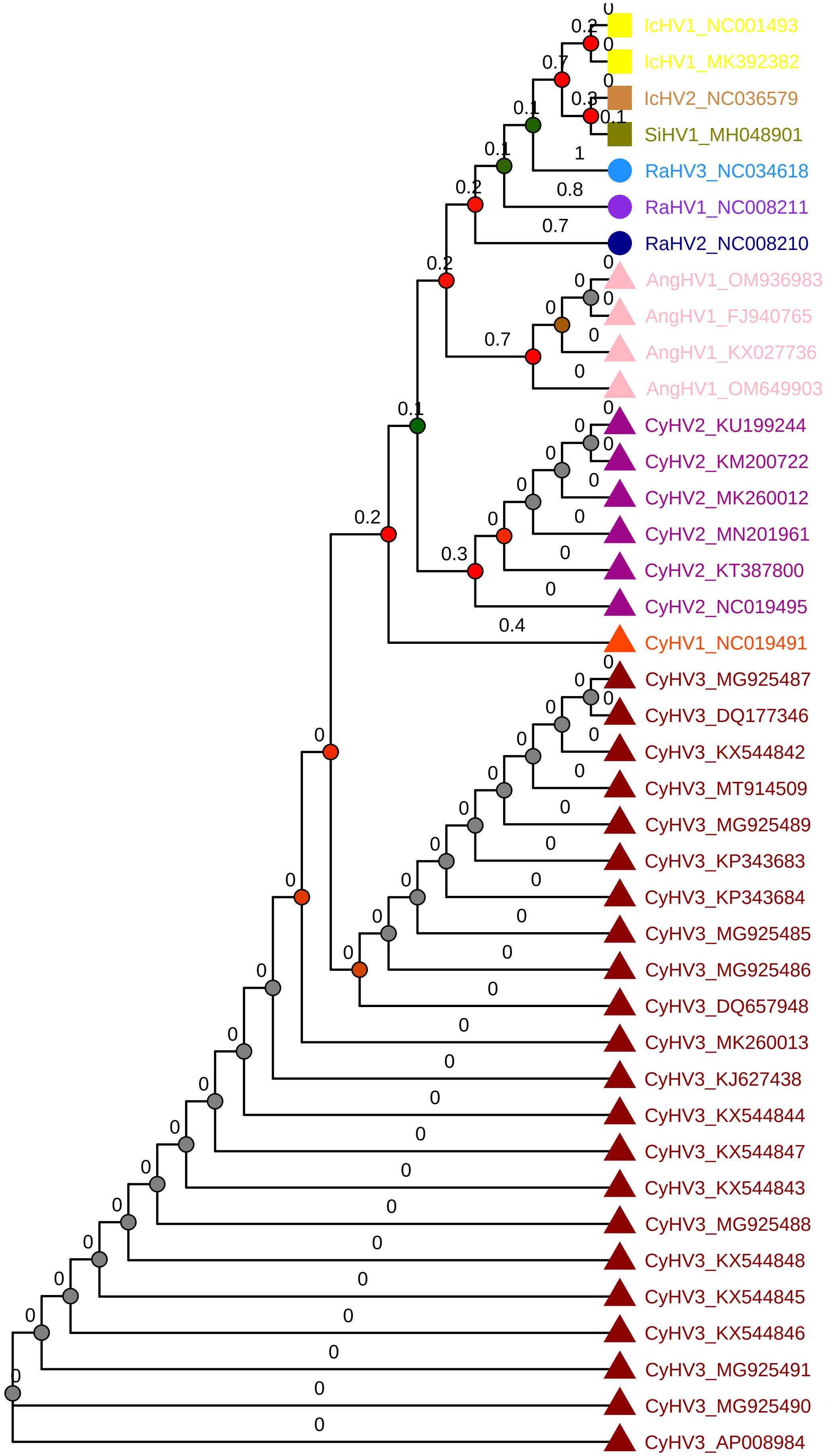

#27 NA: ML method using IQ−TREE

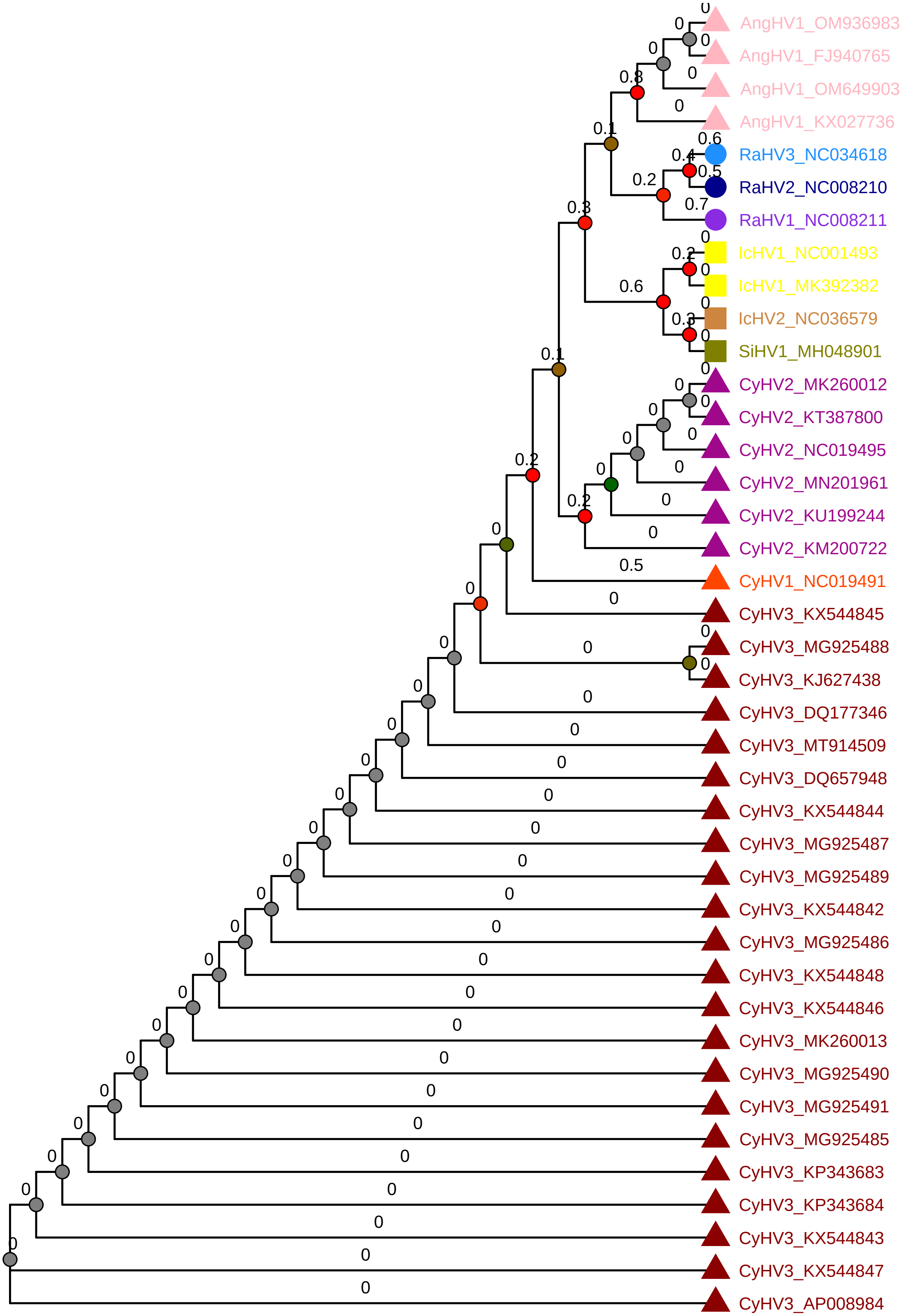

#28 NA: ML method using IQ−TREE

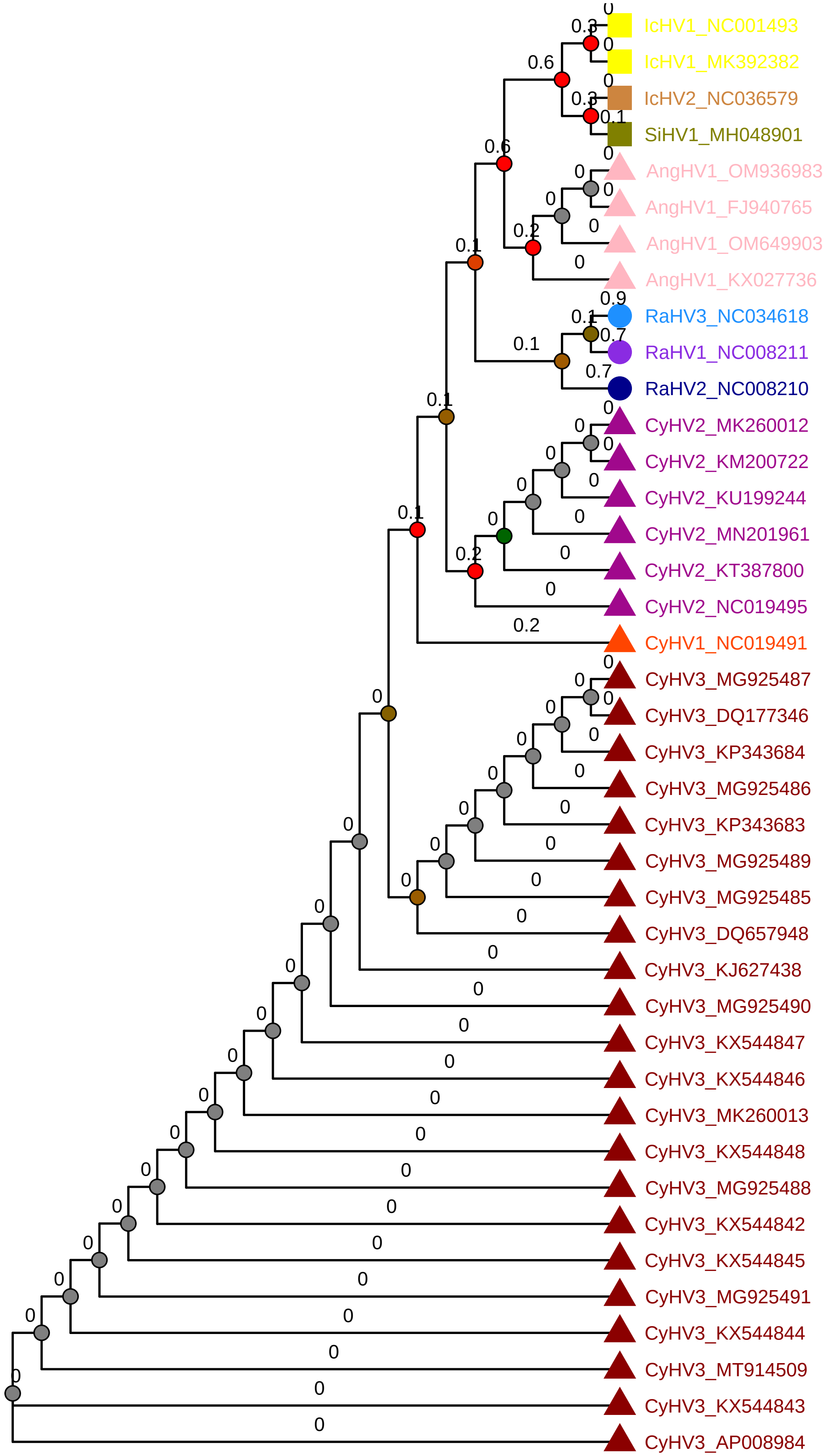

#29 NA: ML method using IQ−TREE

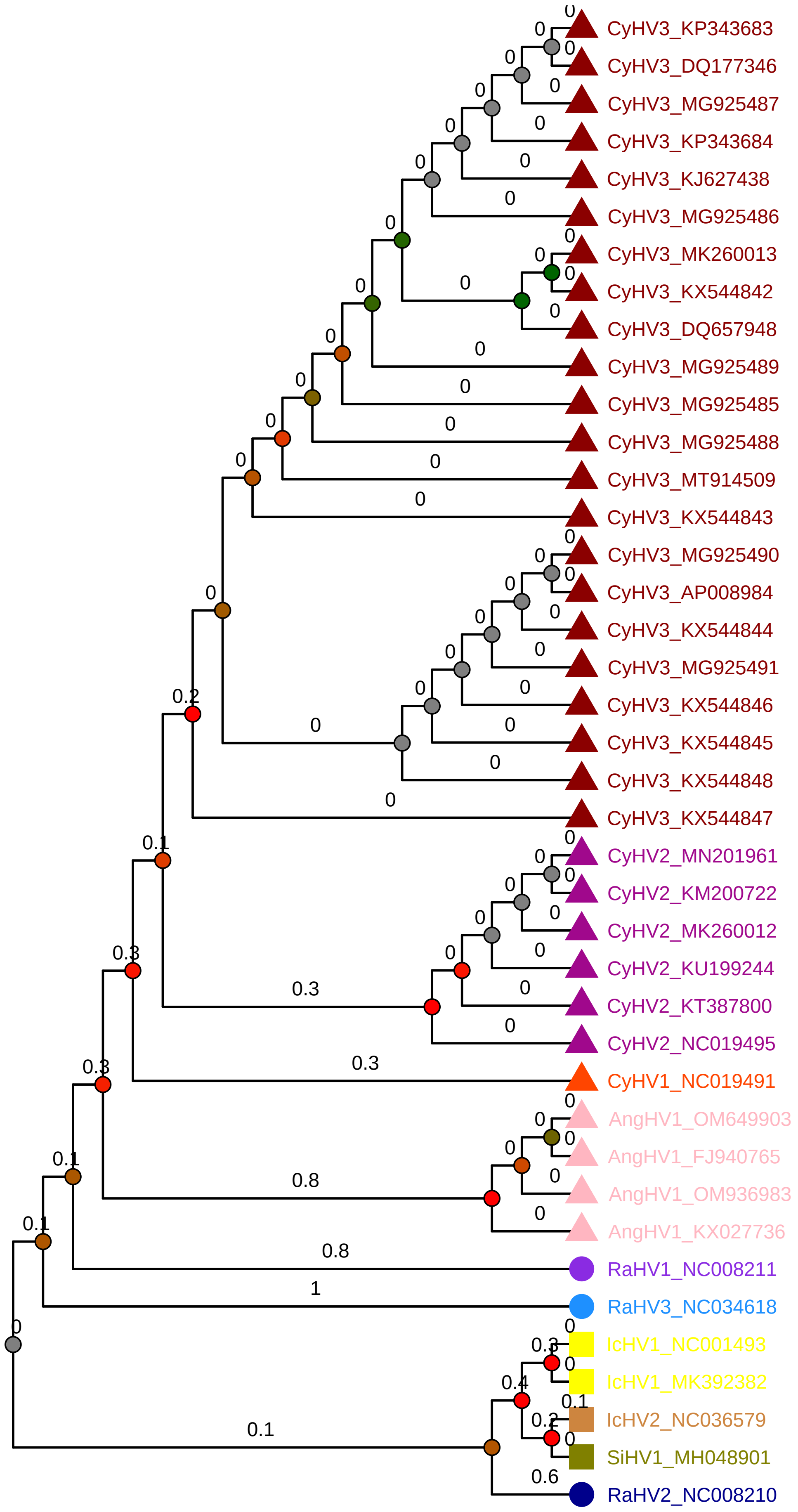

#30 NA: ML method using IQ−TREE

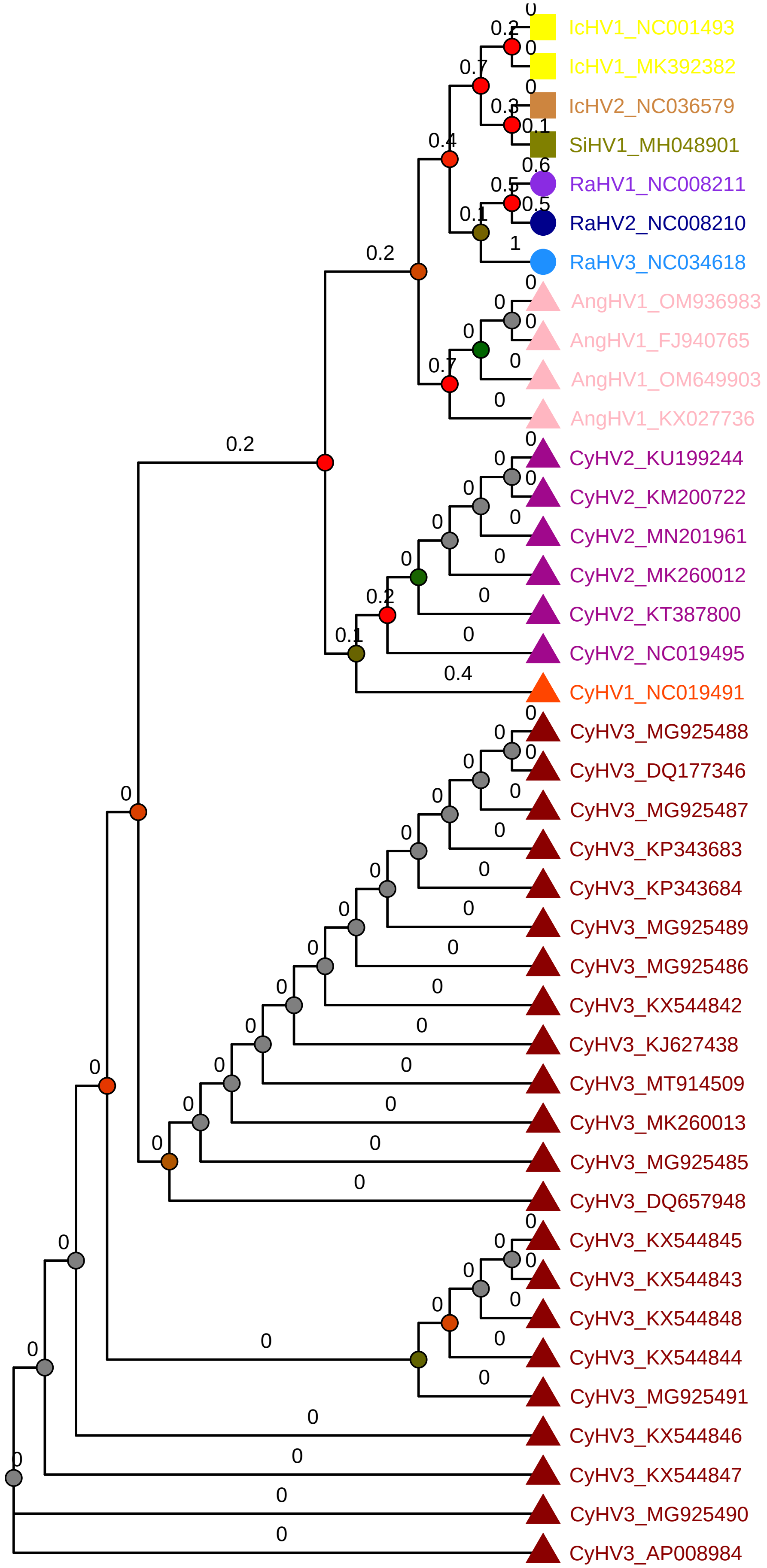

#31 NA: ML method using IQ−TREE

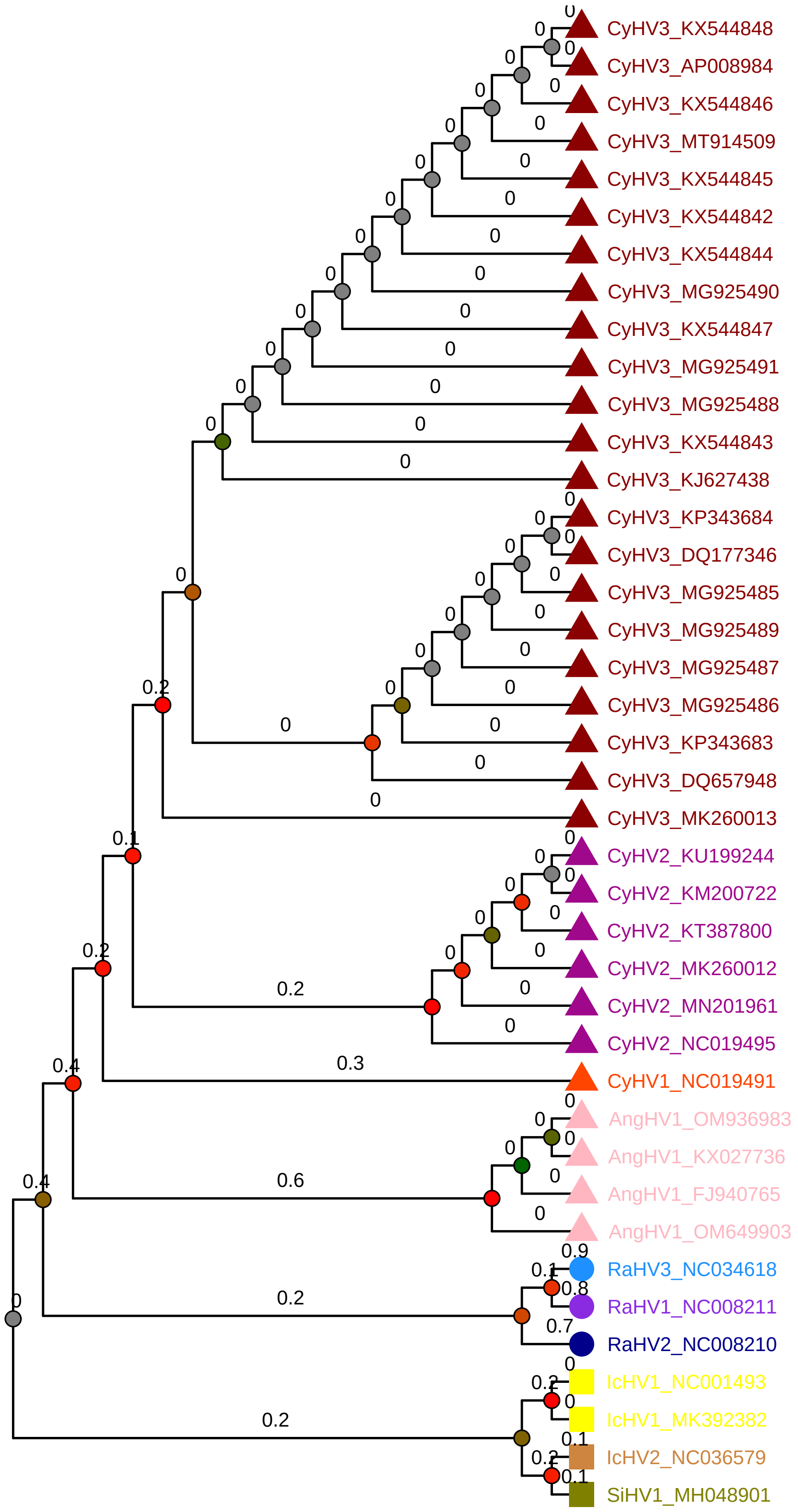

#32 NA: ML method using IQ−TREE

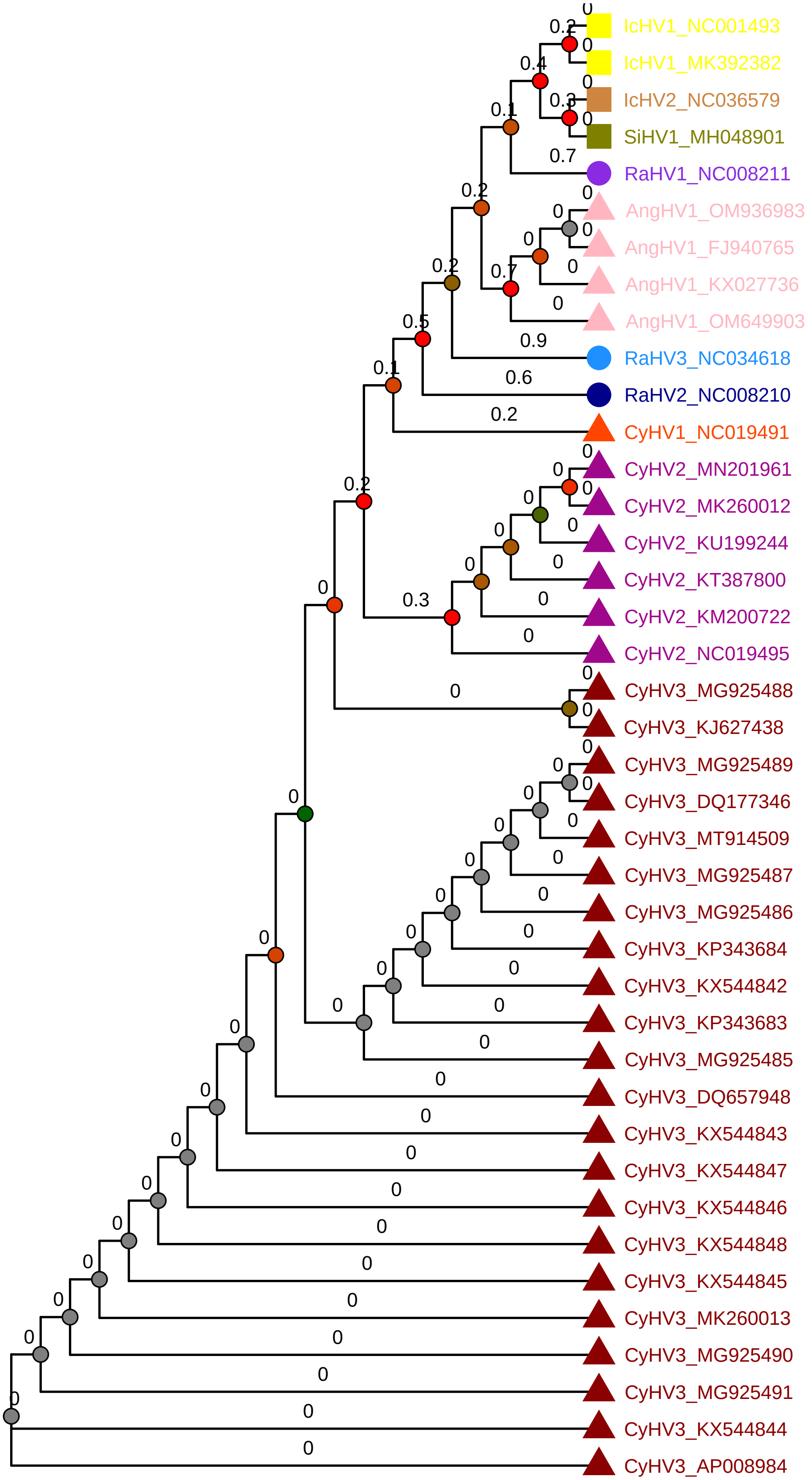

#6 AA: ML method using MEGA

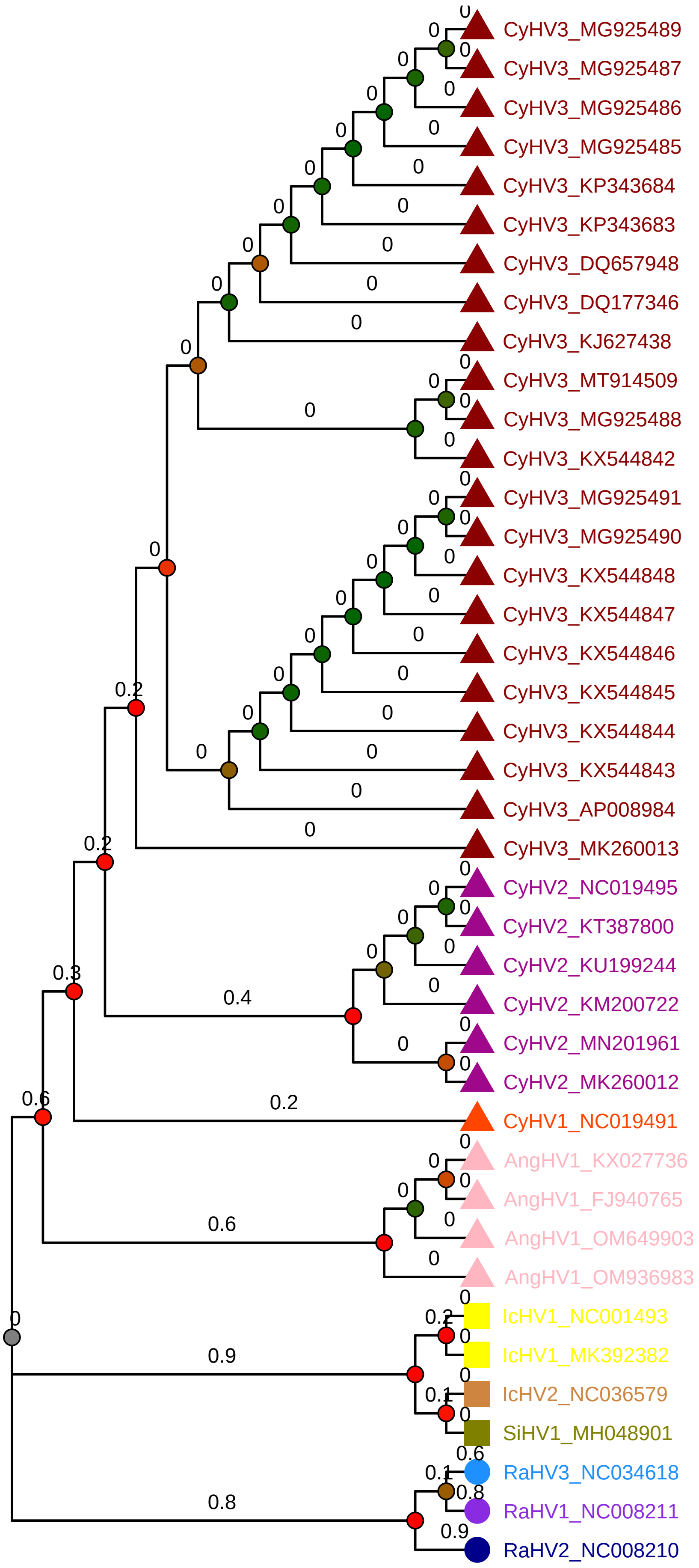

#9 AA: ML method using MEGA

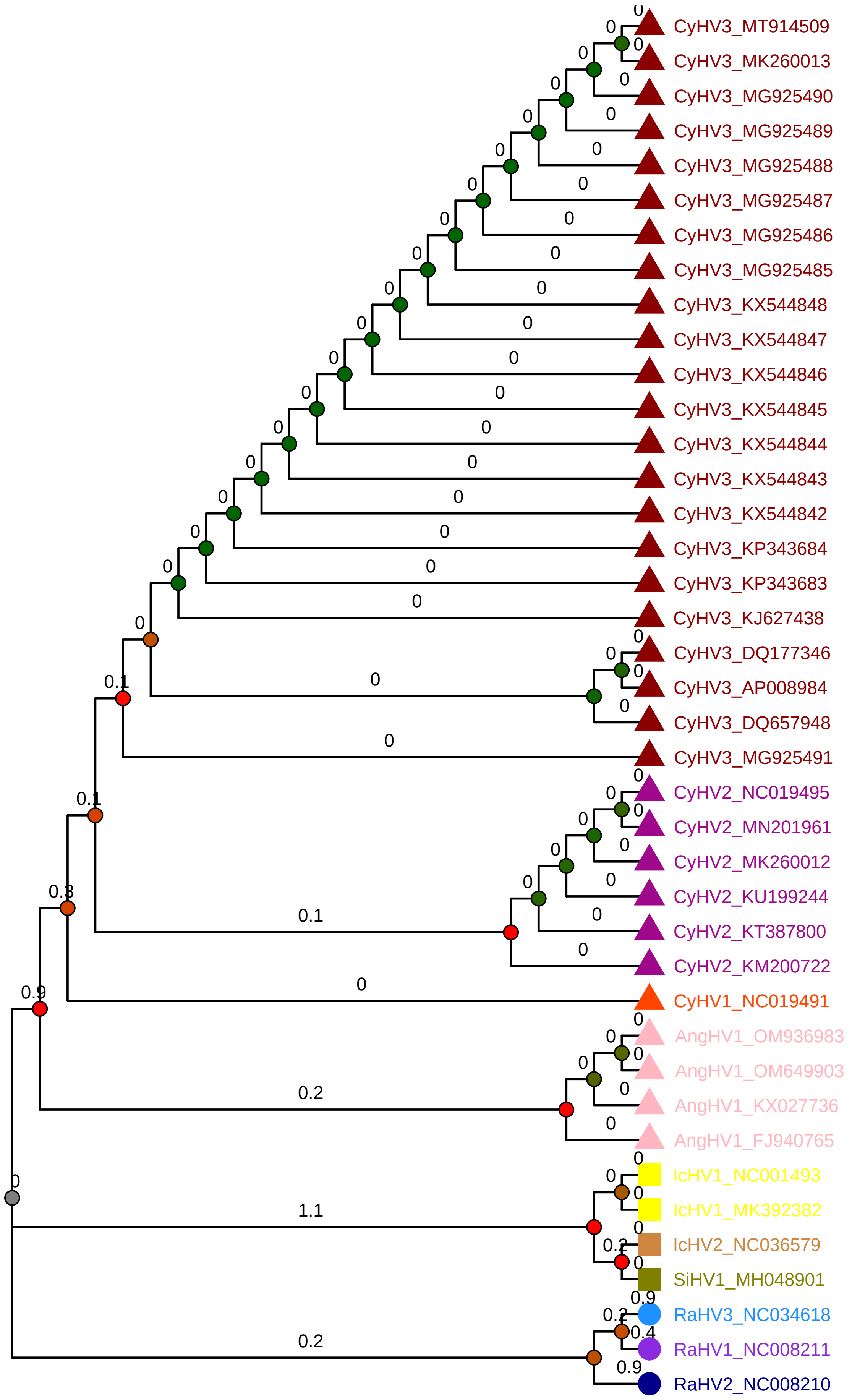

#12 AA: ML method using MEGA

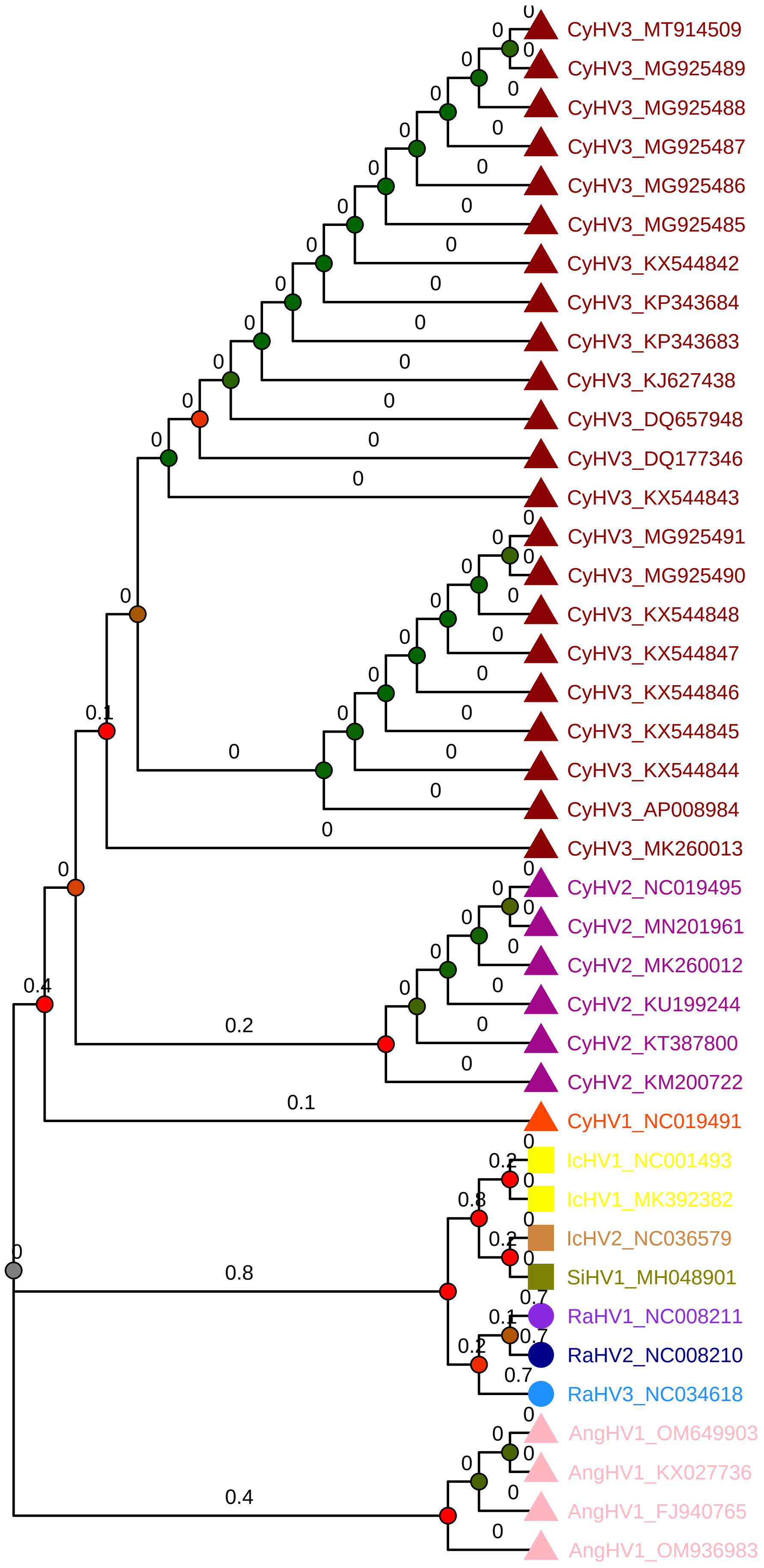

#13 AA: ML method using MEGA

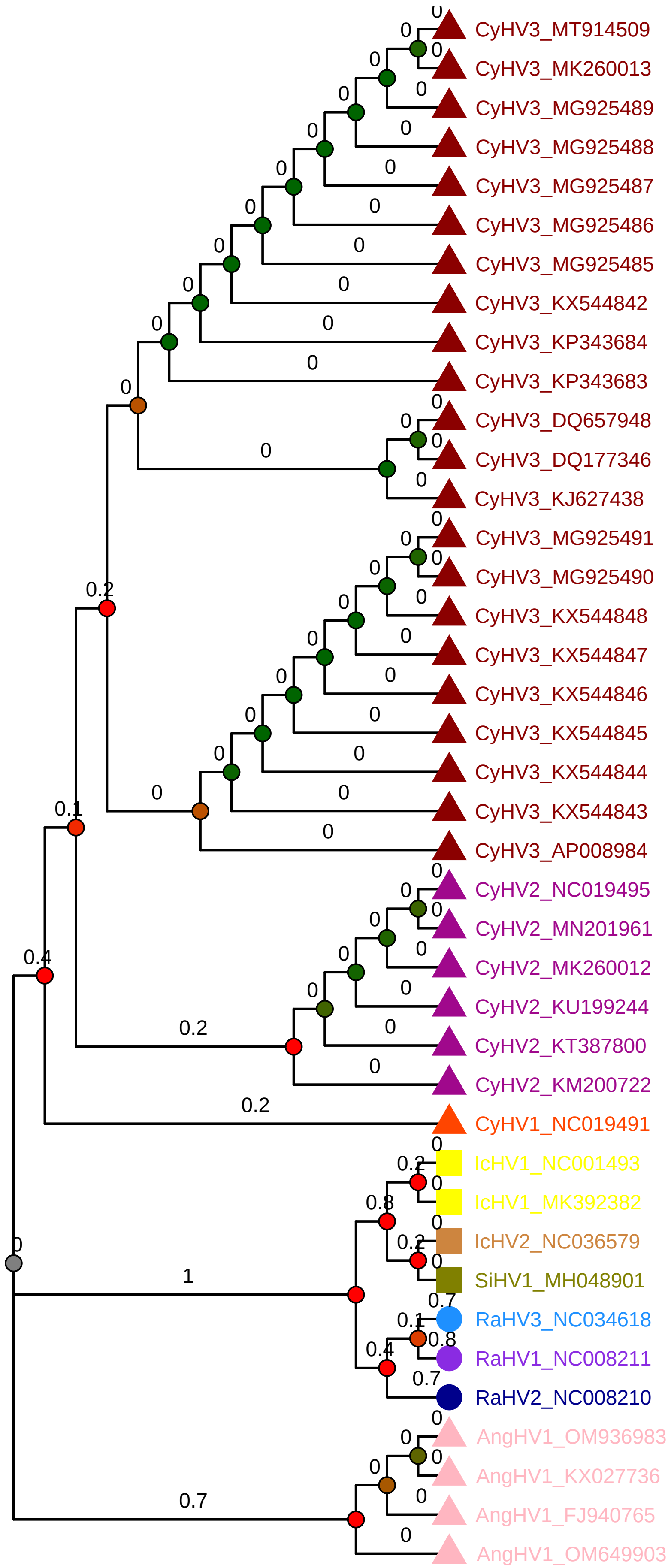

#26 AA: ML method using MEGA

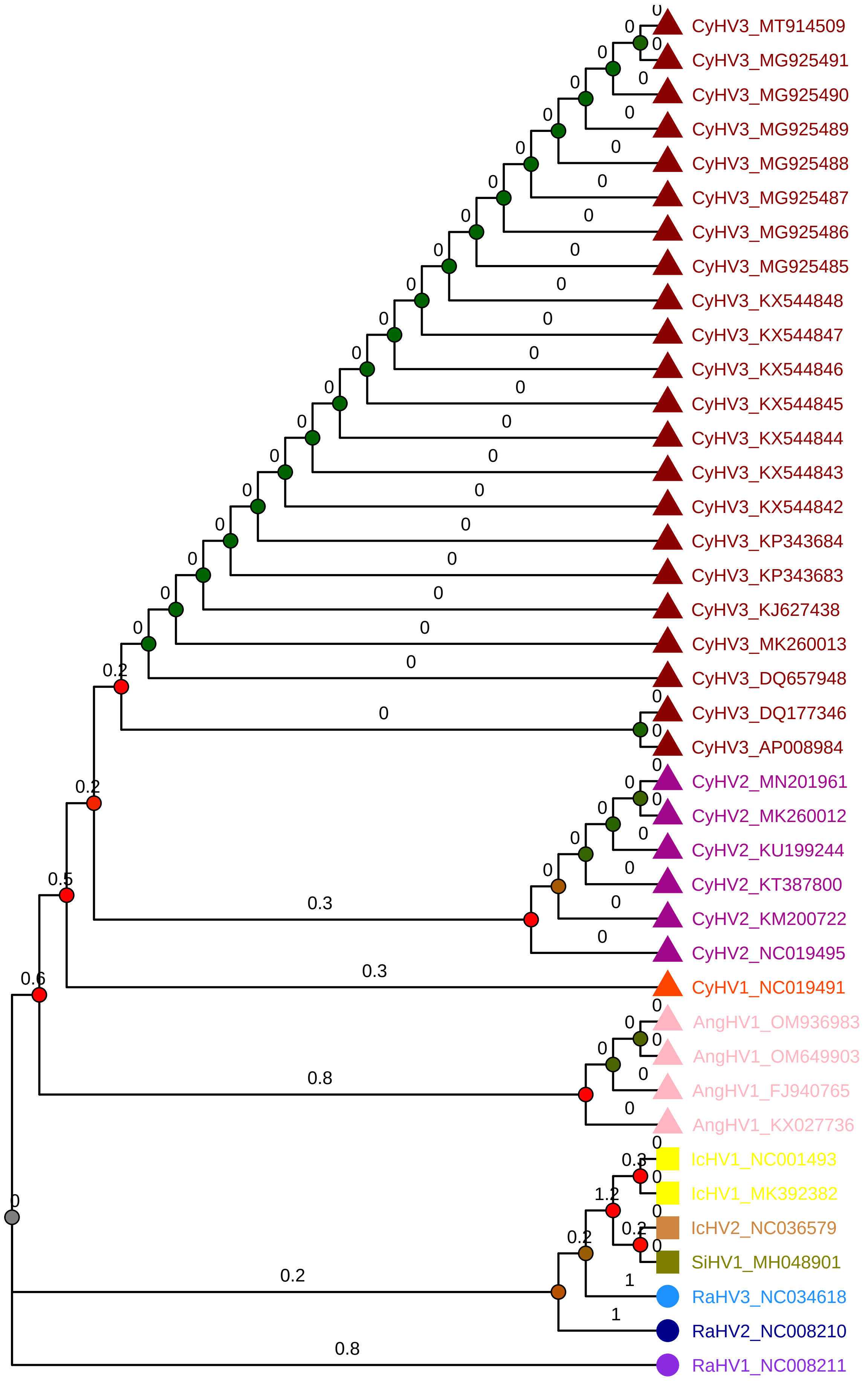

#27 AA: ML method using MEGA

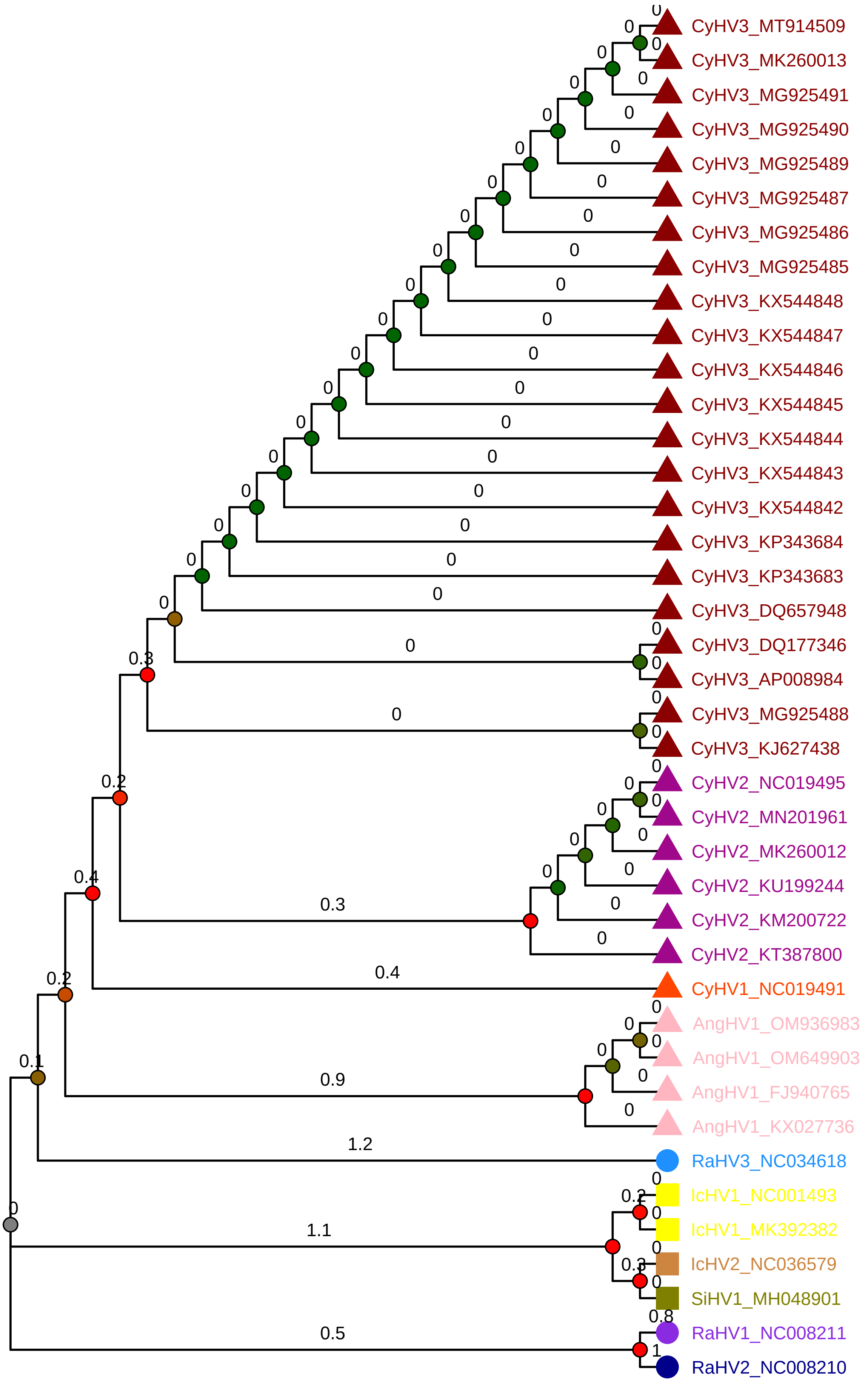

#28 AA: ML method using MEGA

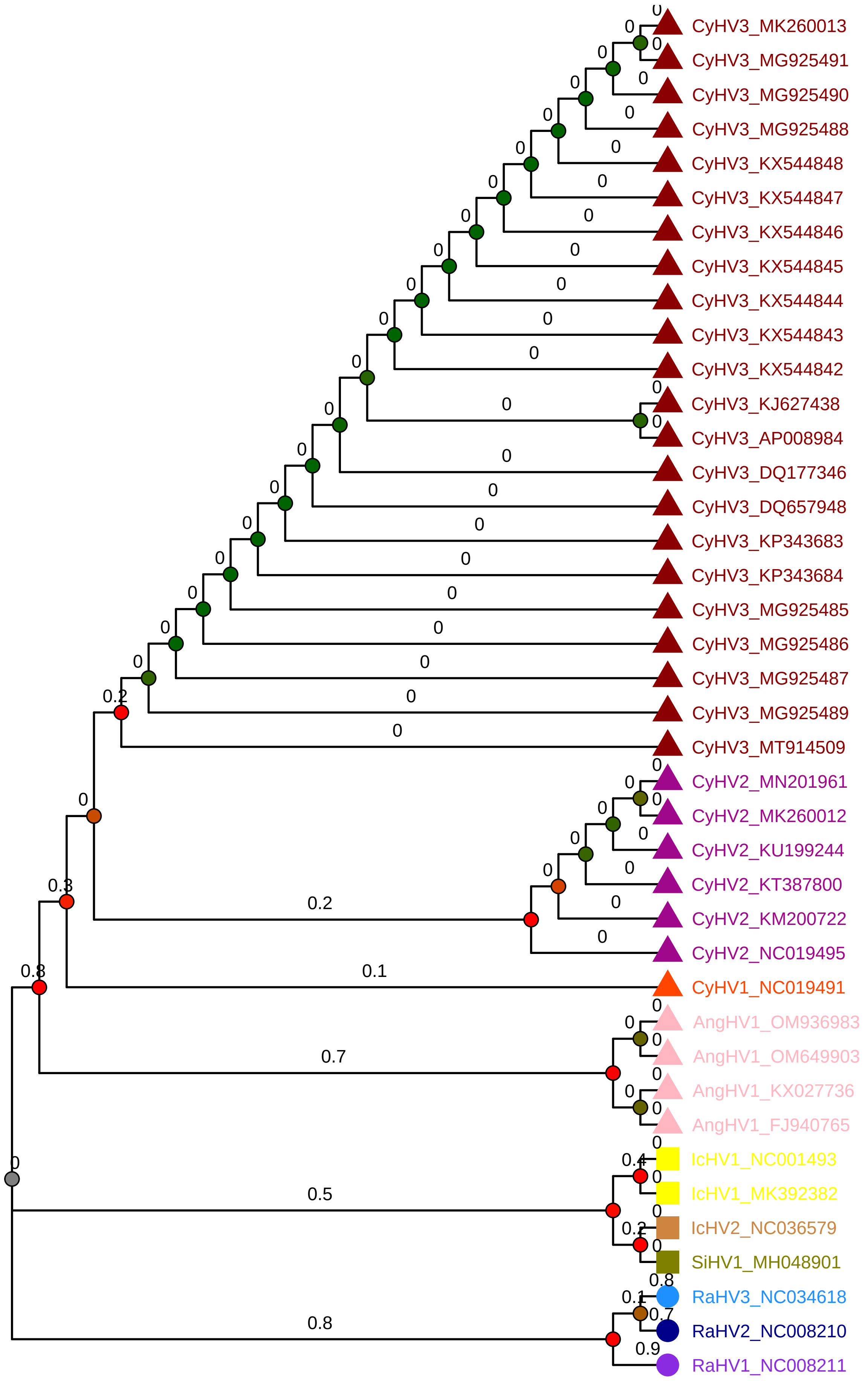

#29 AA: ML method using MEGA

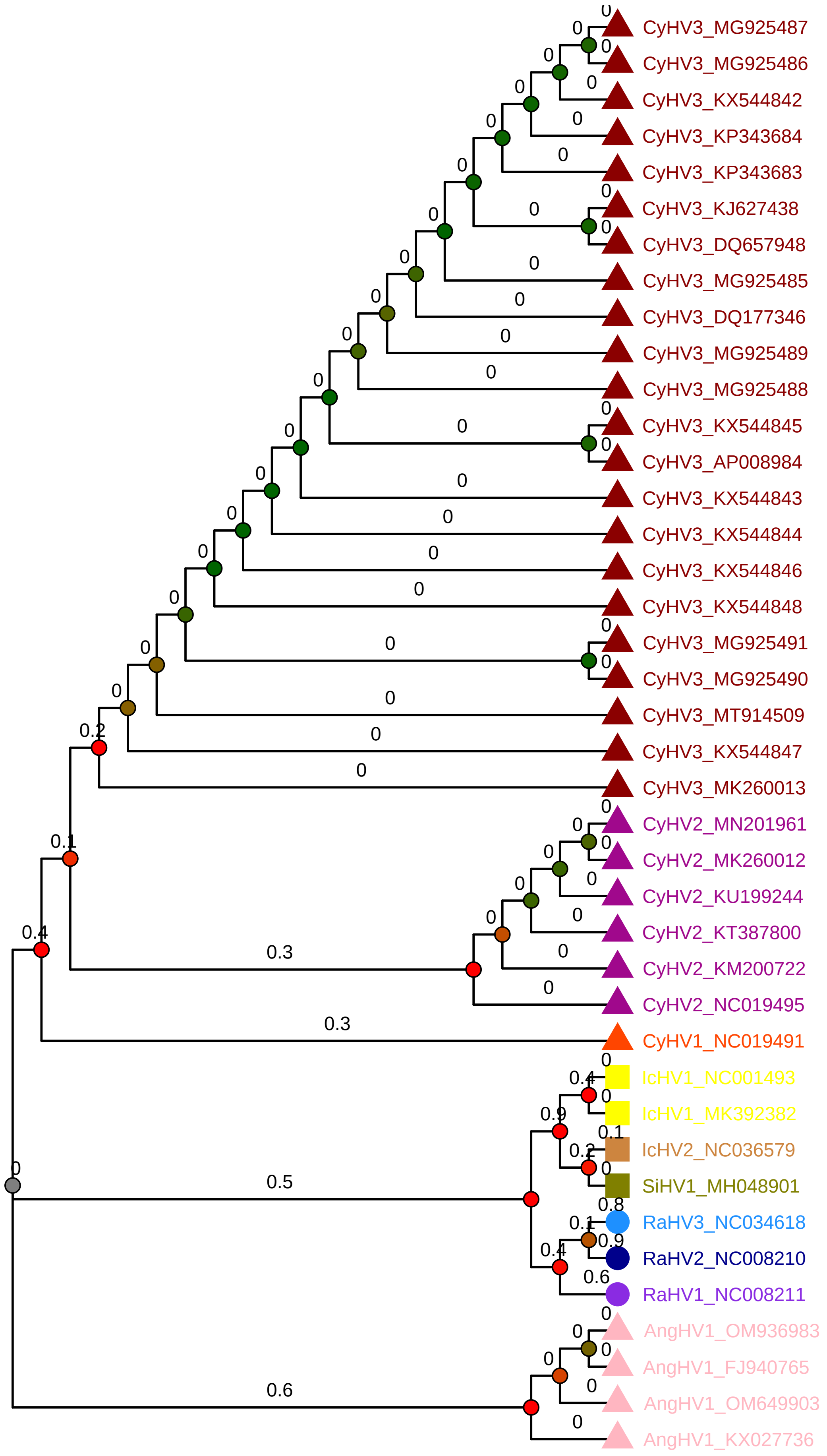

#30 AA: ML method using MEGA

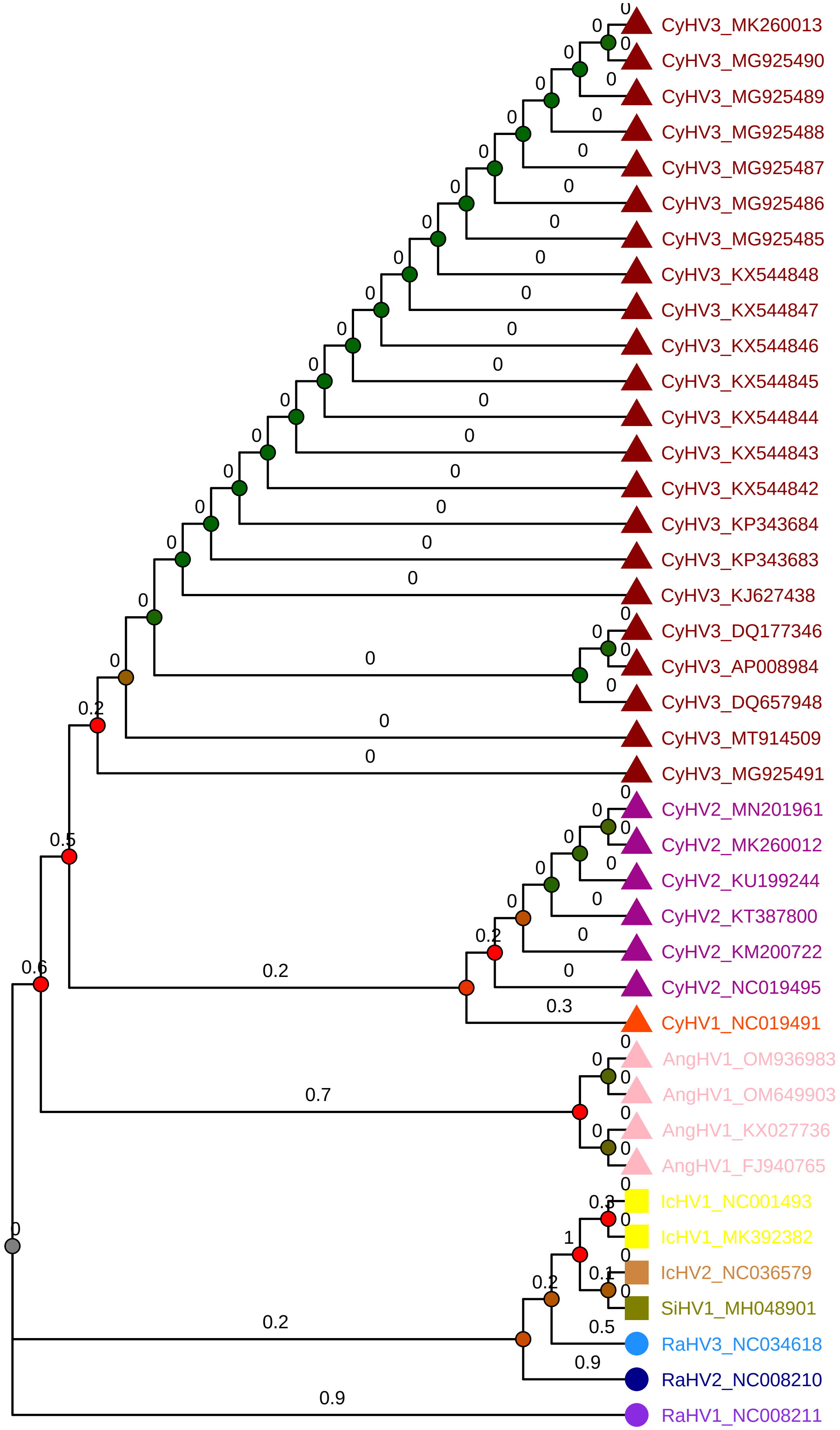

#31 AA: ML method using MEGA

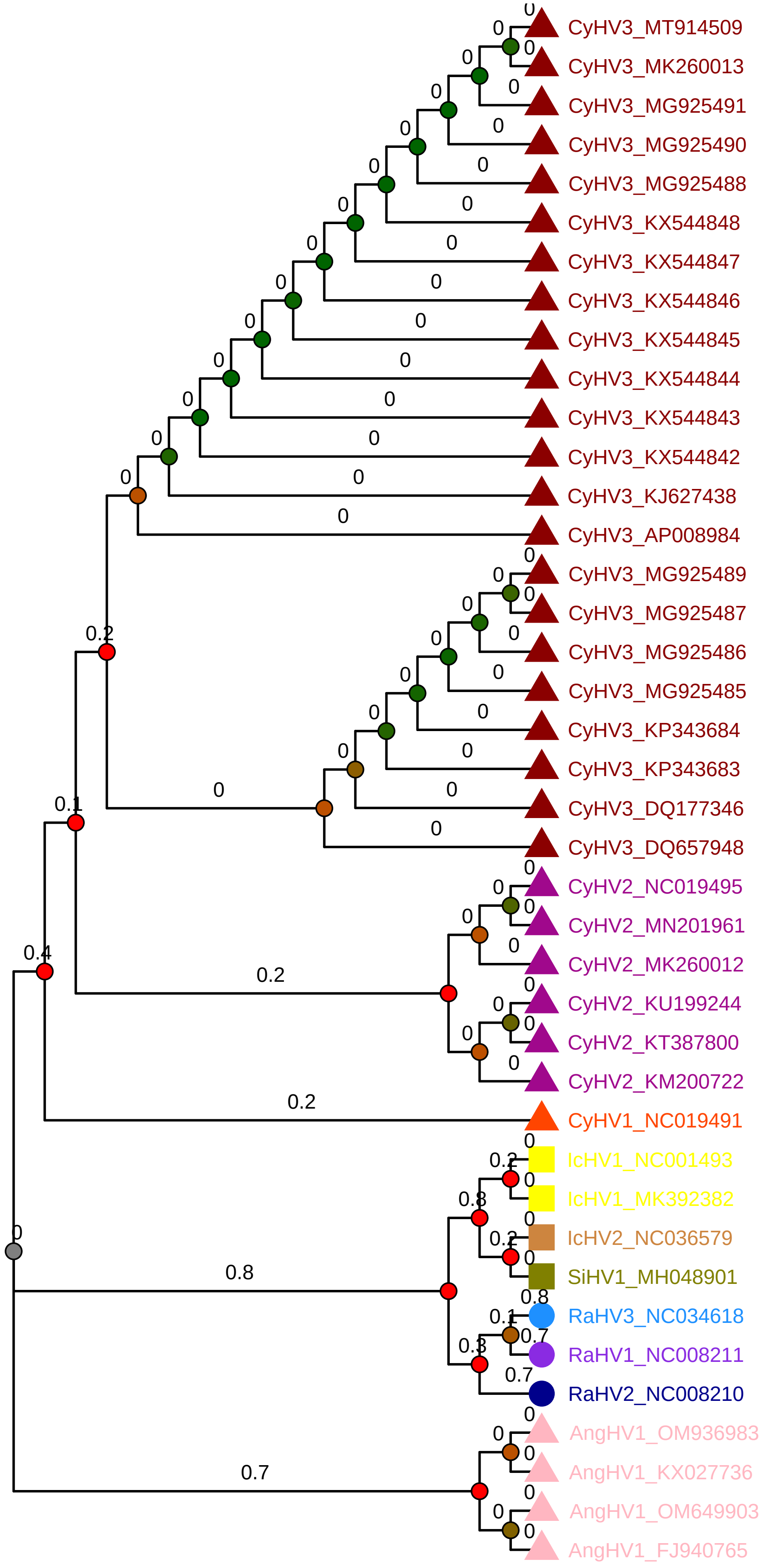

#32 AA: ML method using MEGA

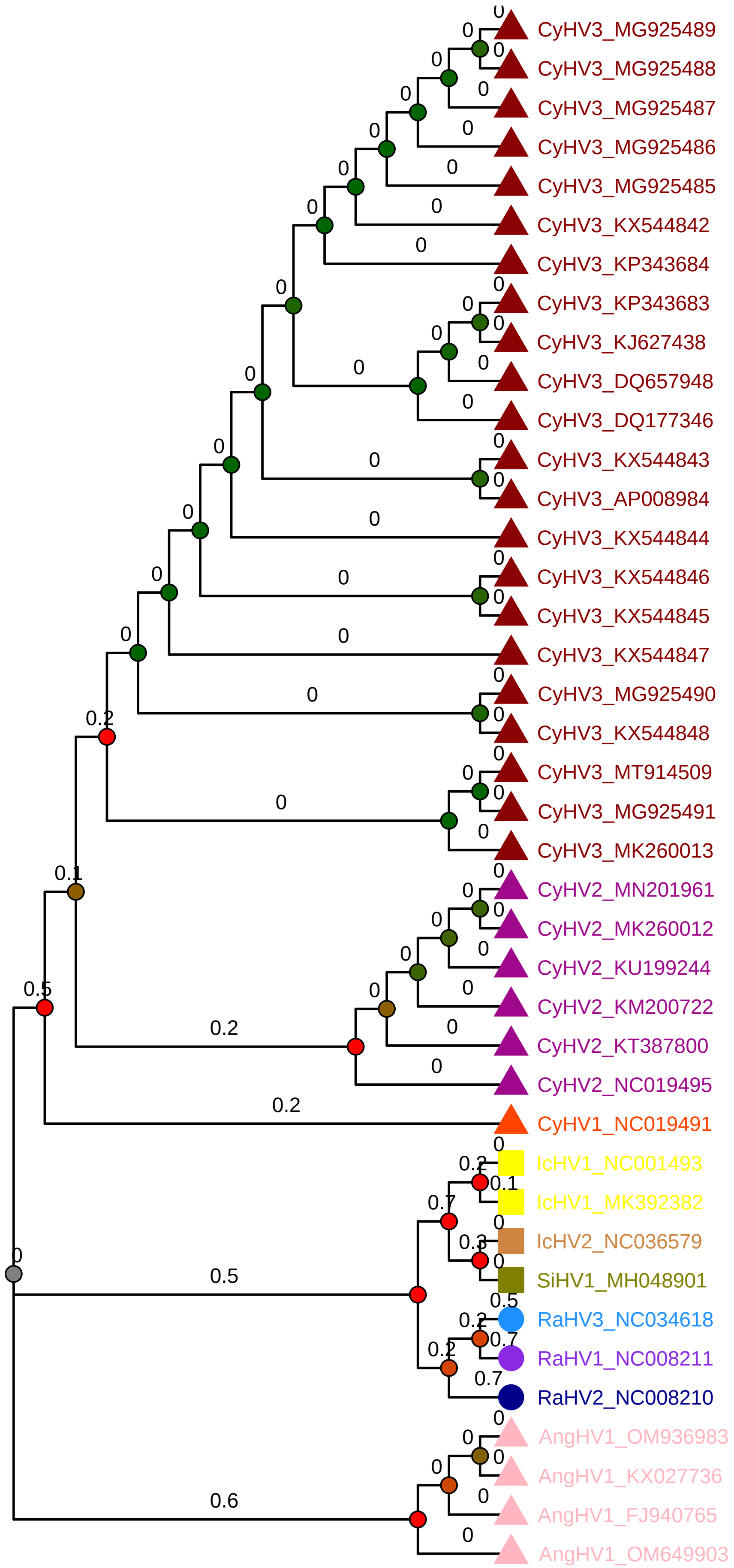

#6 NA: ML method using MEGA

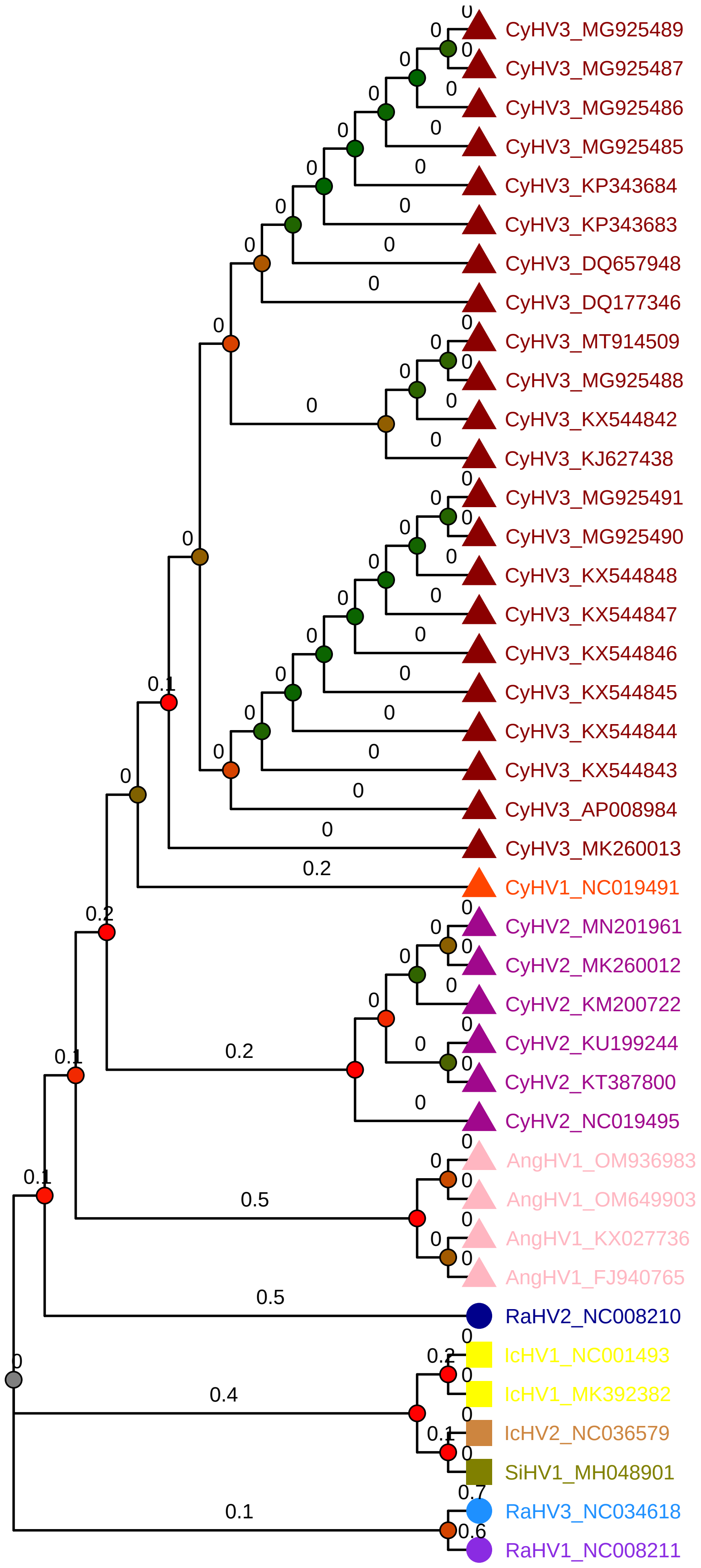

#9 NA: ML method using MEGA

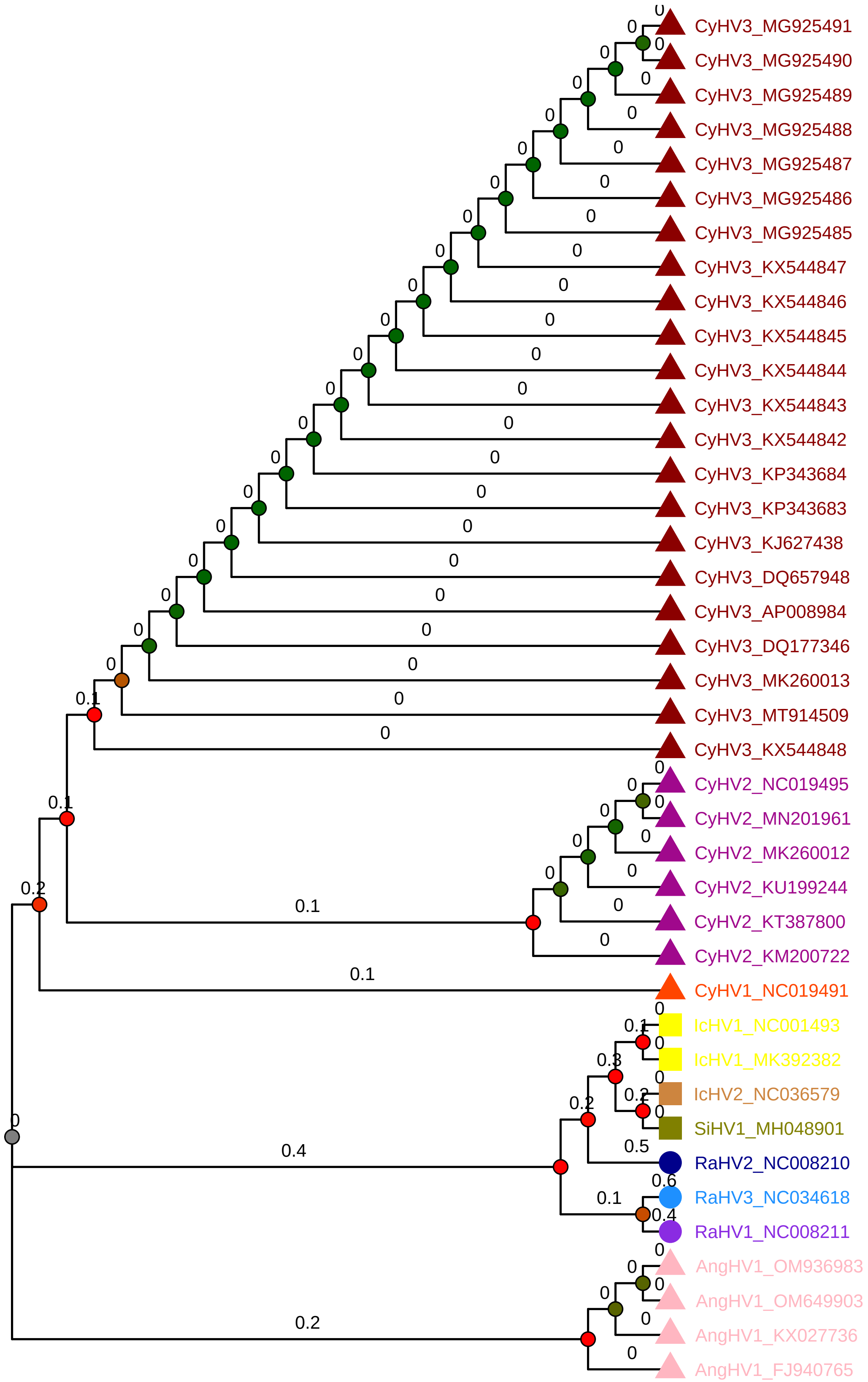

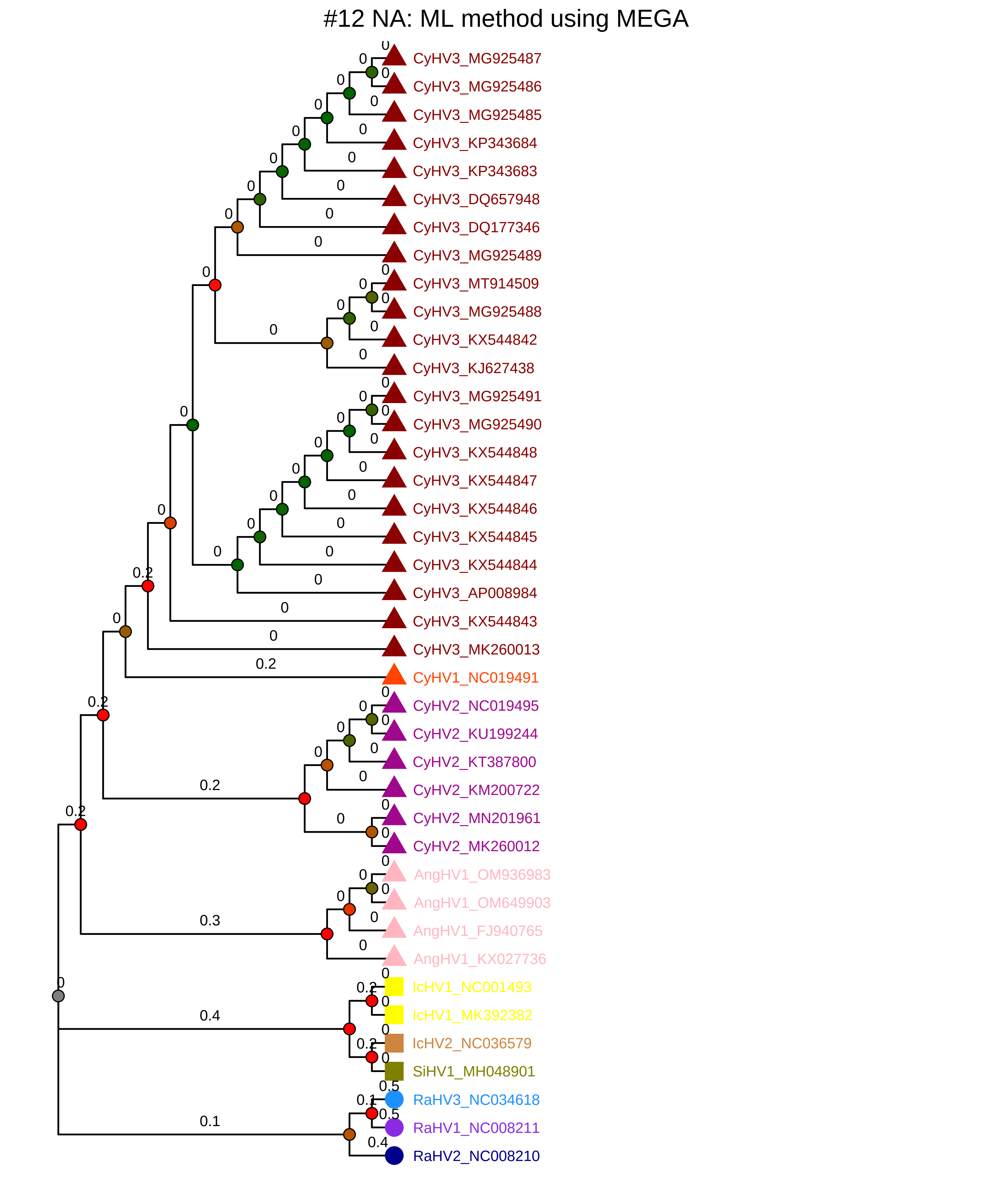
#12 NA: ML method using MEGA
CyHV3_MG925487
CyHV3_MG925486
CyHV3_MG925485
CyHV3_KP343684
CyHV3_KP343683
CyHV3_DQ657948
CyHV3_DQ177346
CyHV3_MG925489
CyHV3_MT914509
CyHV3_MG925488
CyHV3_KX544842
CyHV3_KJ627438
CyHV3_MG925491
CyHV3_MG925490
CyHV3_KX544848
CyHV3_KX544847
CyHV3_KX544846
CyHV3_KX544845
CyHV3_KX544844
CyHV3_AP008984
CyHV3_KX544843
CyHV3_MK260013
CyHV1_NC019491
CyHV2_NC019495
CyHV2_KU199244
CyHV2_KT387800
CyHV2_KM200722
CyHV2_MN201961
CyHV2_MK260012
AngHV1_OM936983
AngHV1_OM649903
AngHV1_FJ940765
AngHV1_KX027736
IcHV1_NC001493
IcHV1_MK392382
IcHV2_NC036579
SiHV1_MH048901
RaHV3_NC034618
RaHV1_NC008211
RaHV2_NC008210

#13 NA: ML method using MEGA

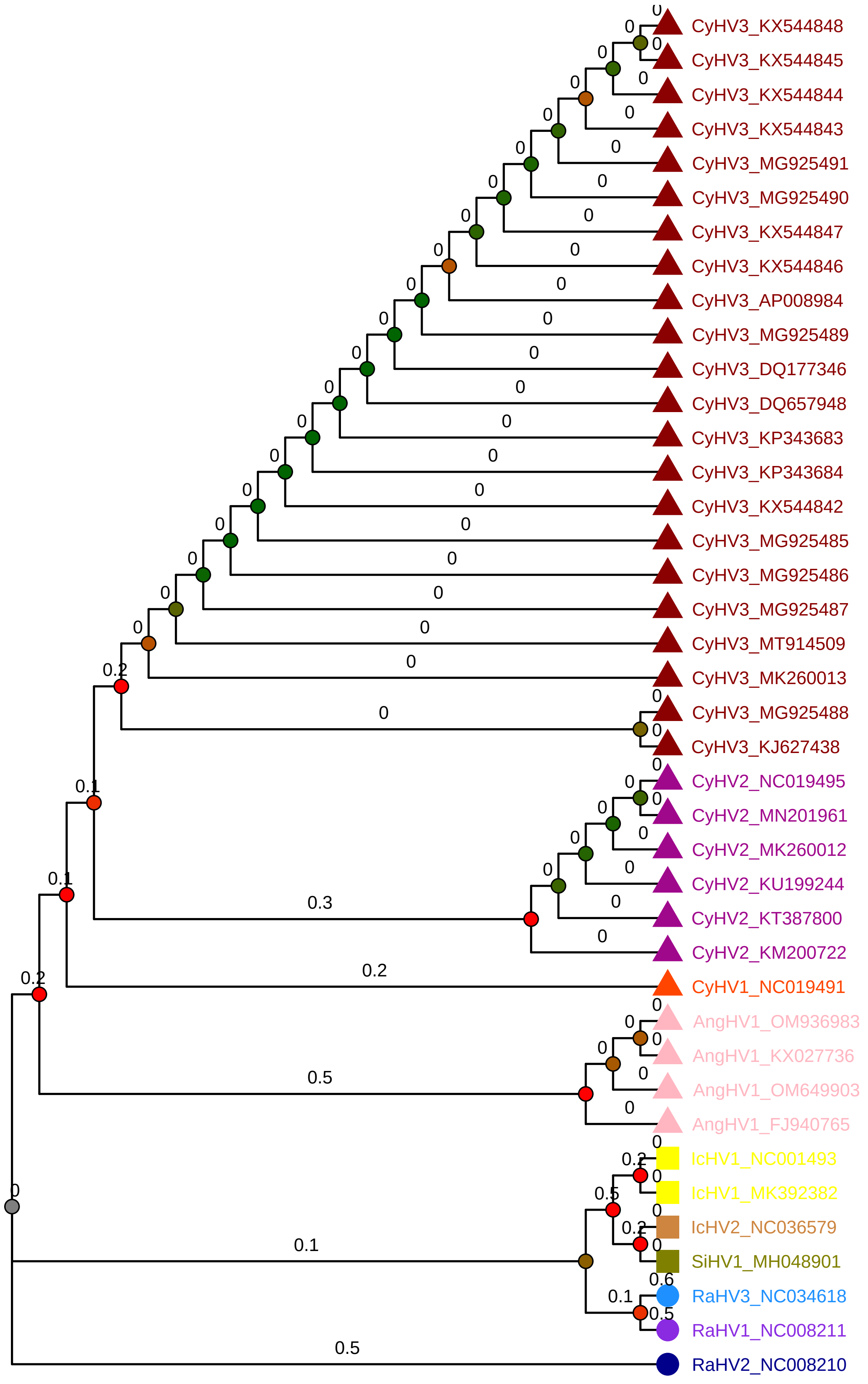

#26 NA: ML method using MEGA

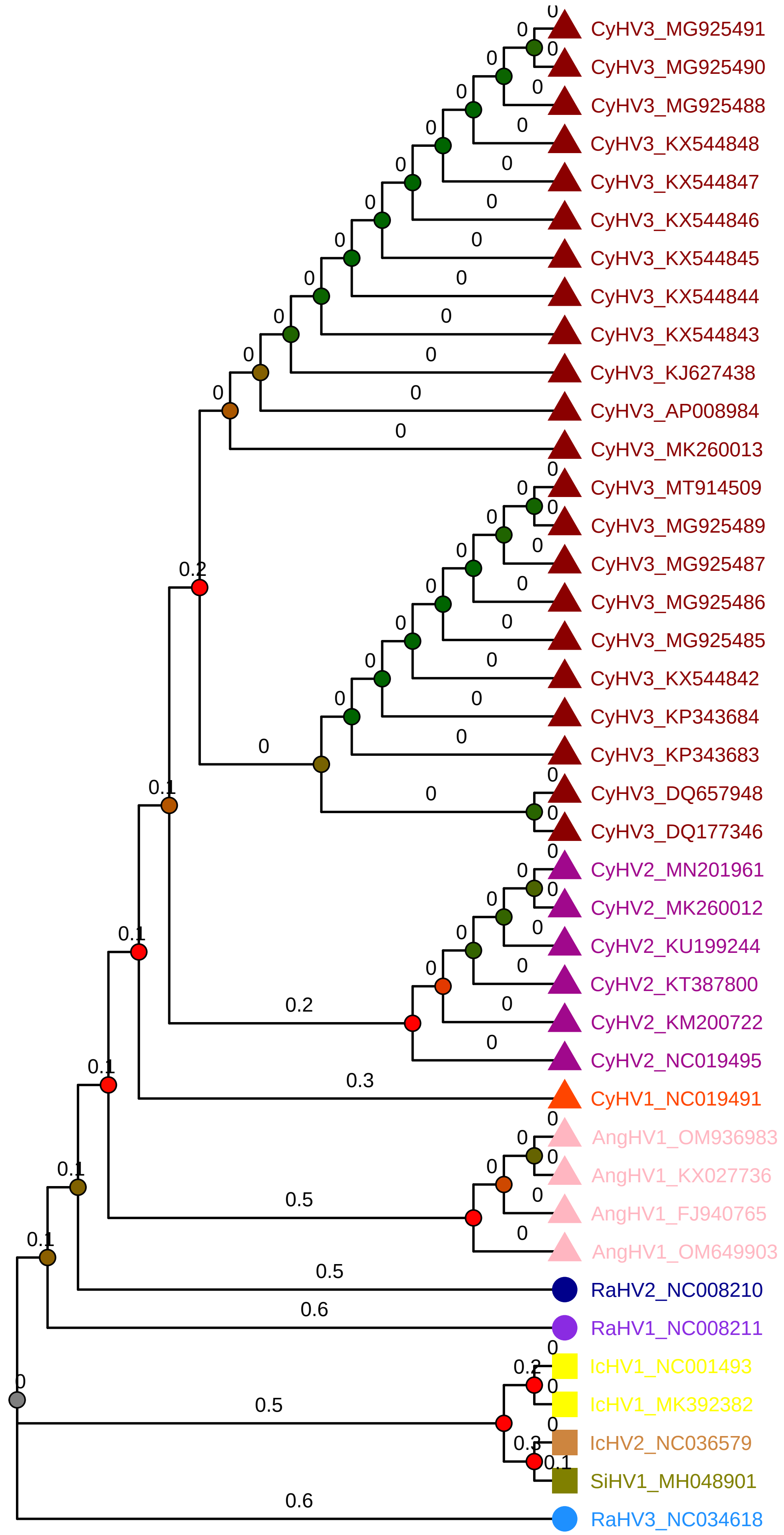

#27 NA: ML method using MEGA

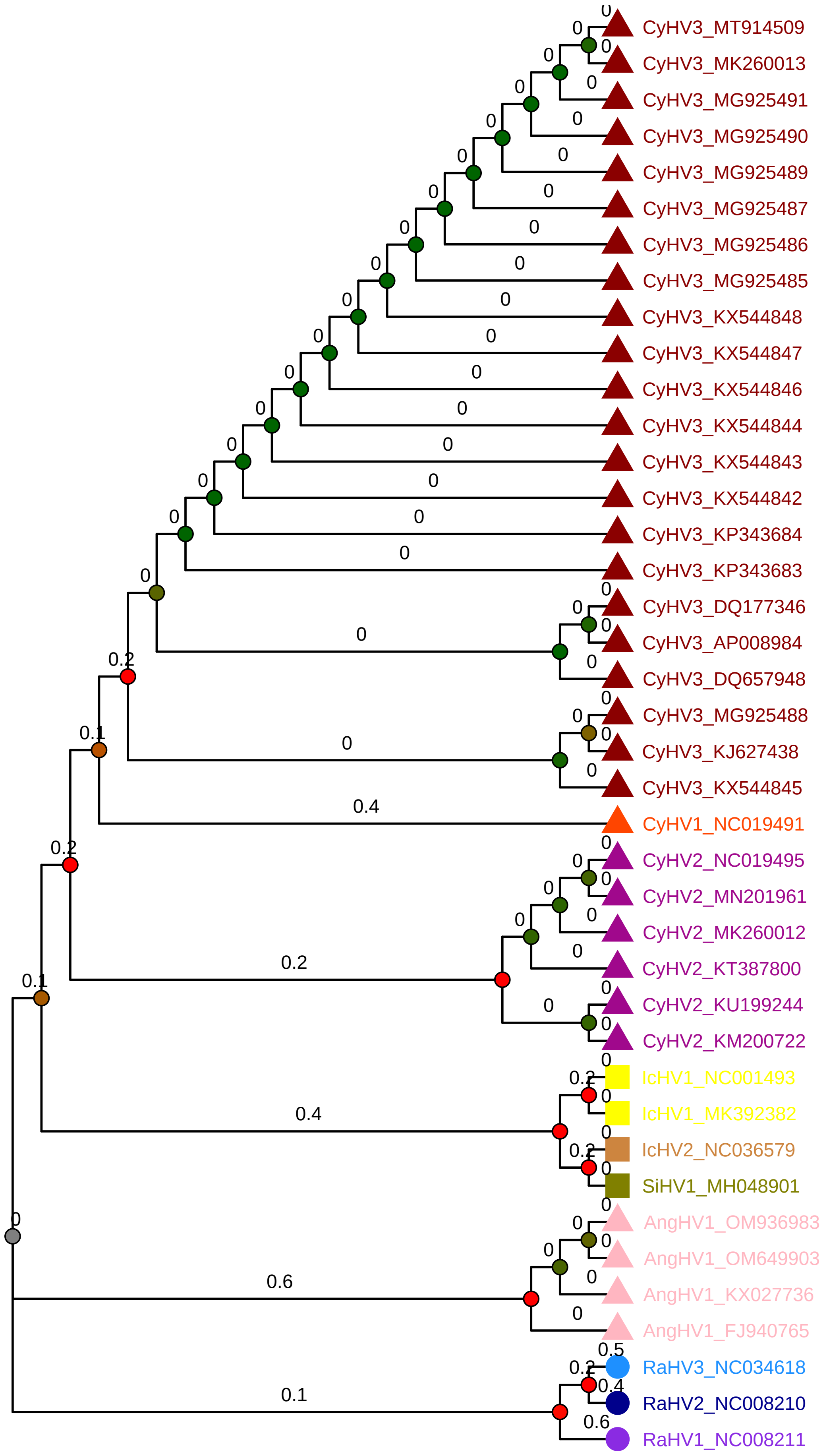

#28 NA: ML method using MEGA

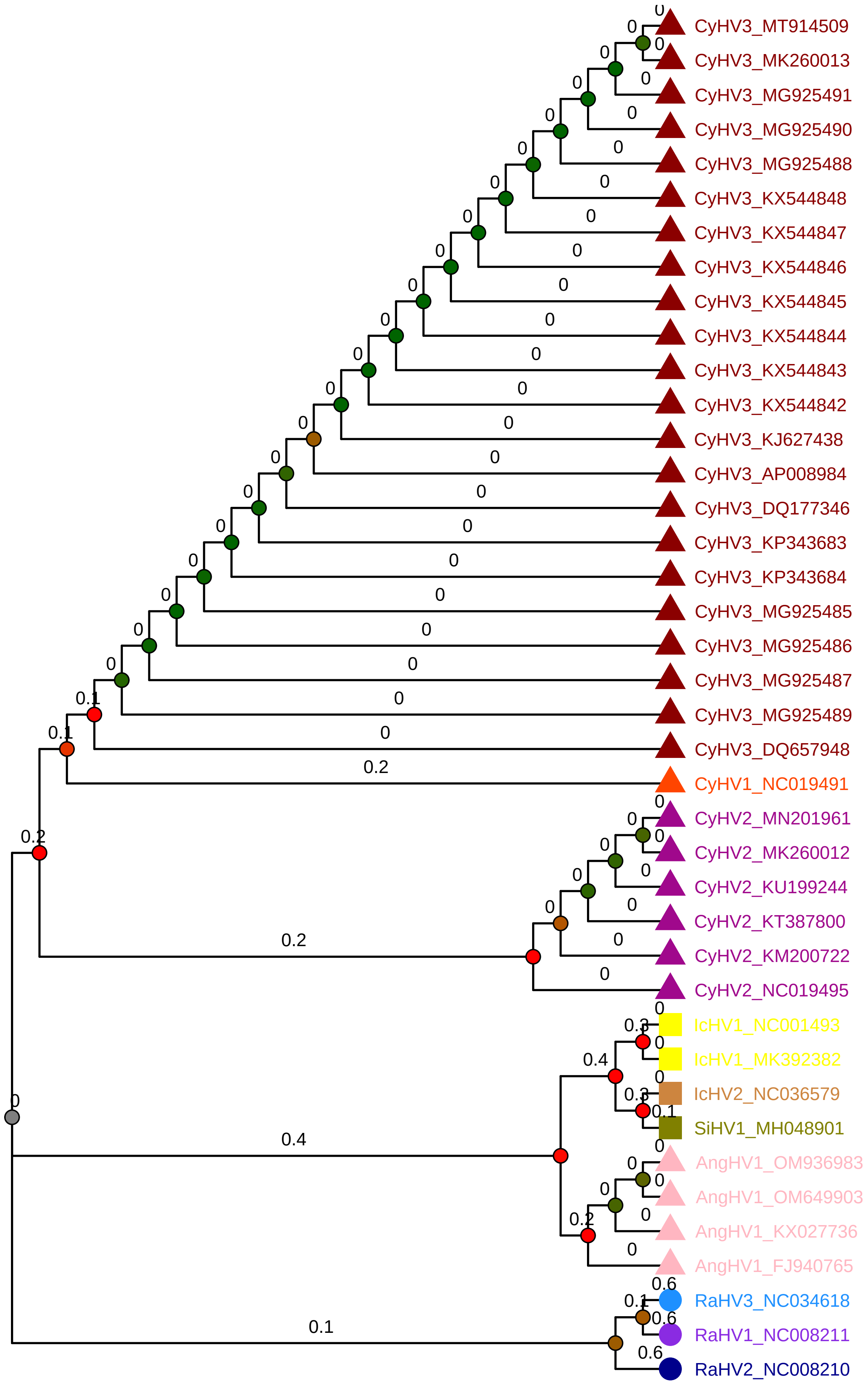

#29 NA: ML method using MEGA

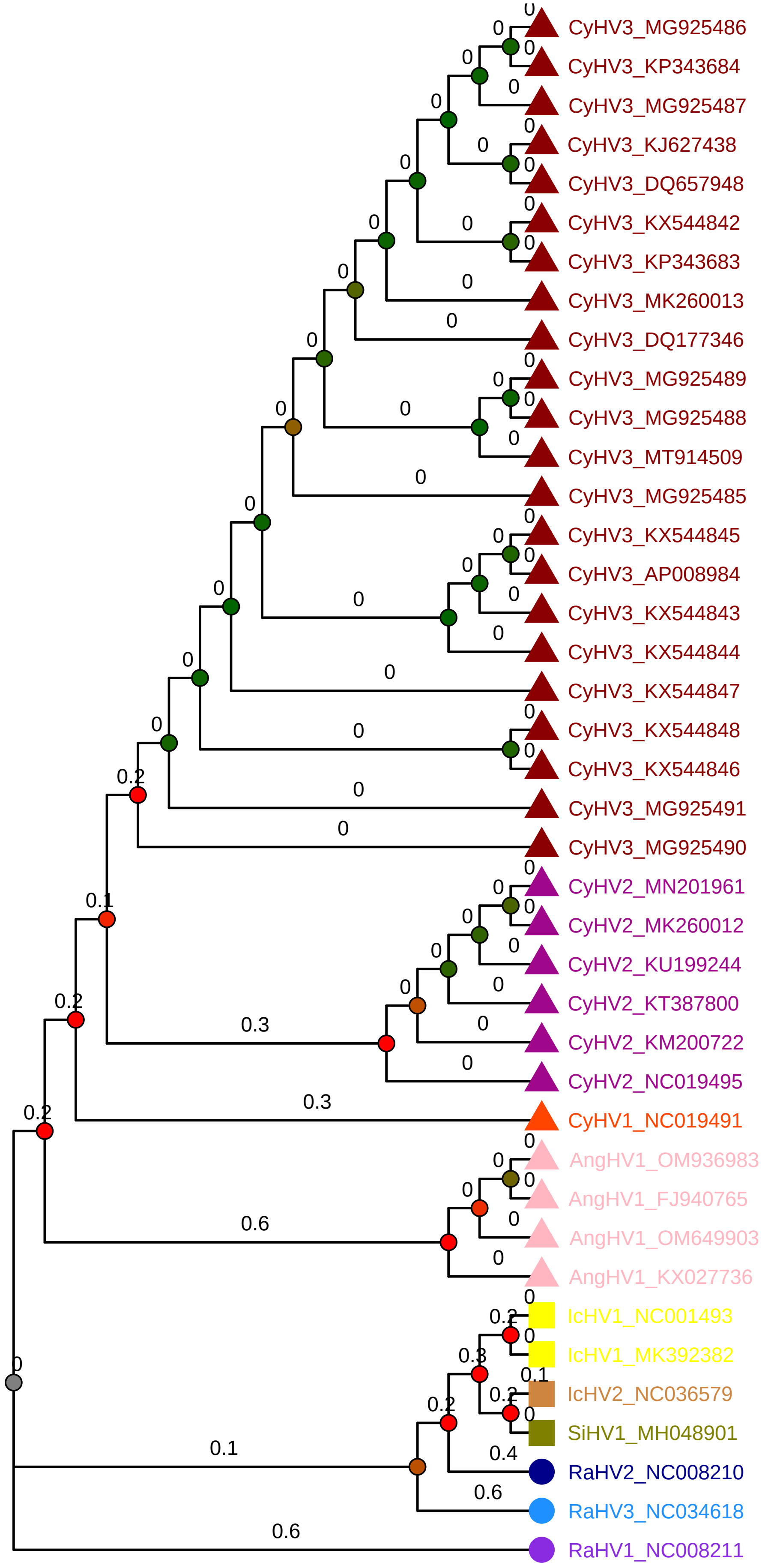

#30 NA: ML method using MEGA

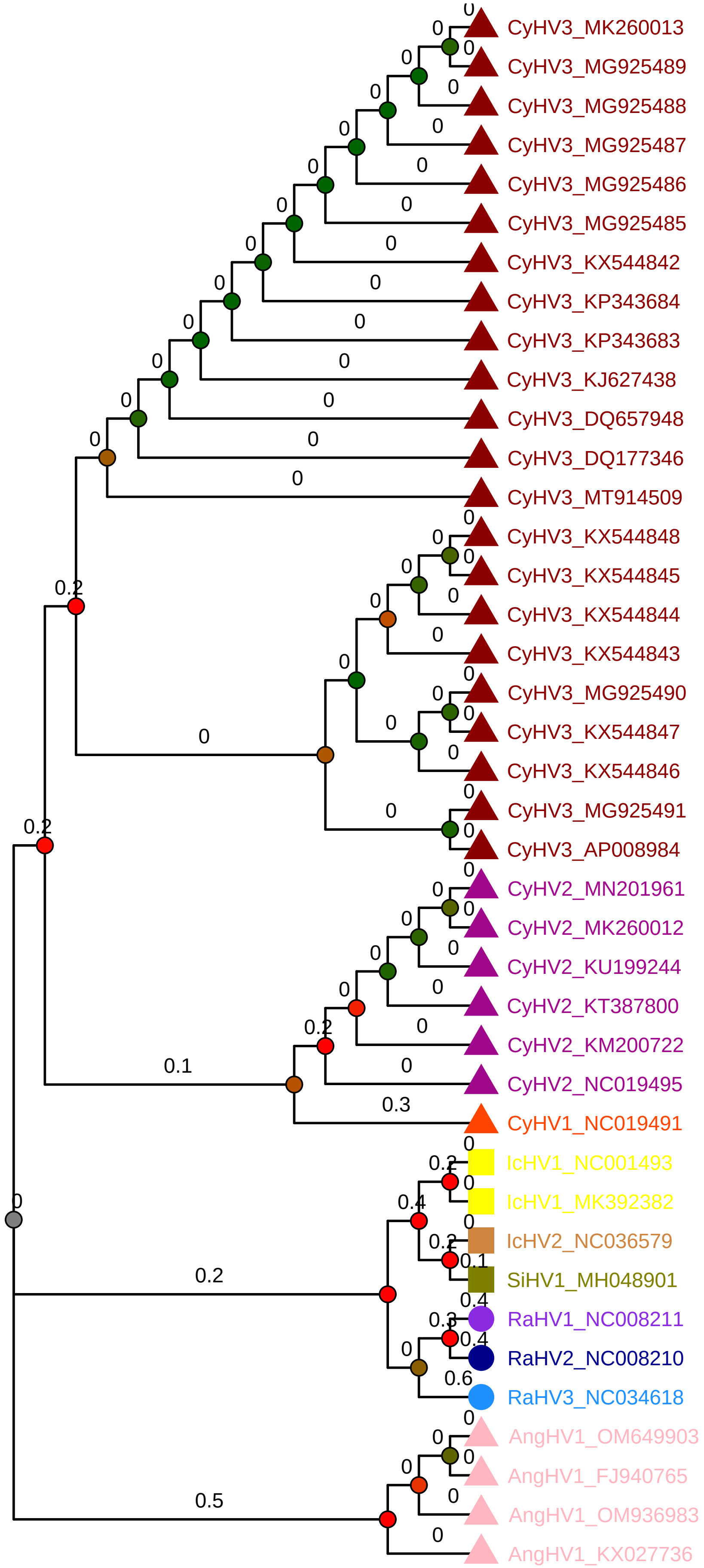

#31 NA: ML method using MEGA

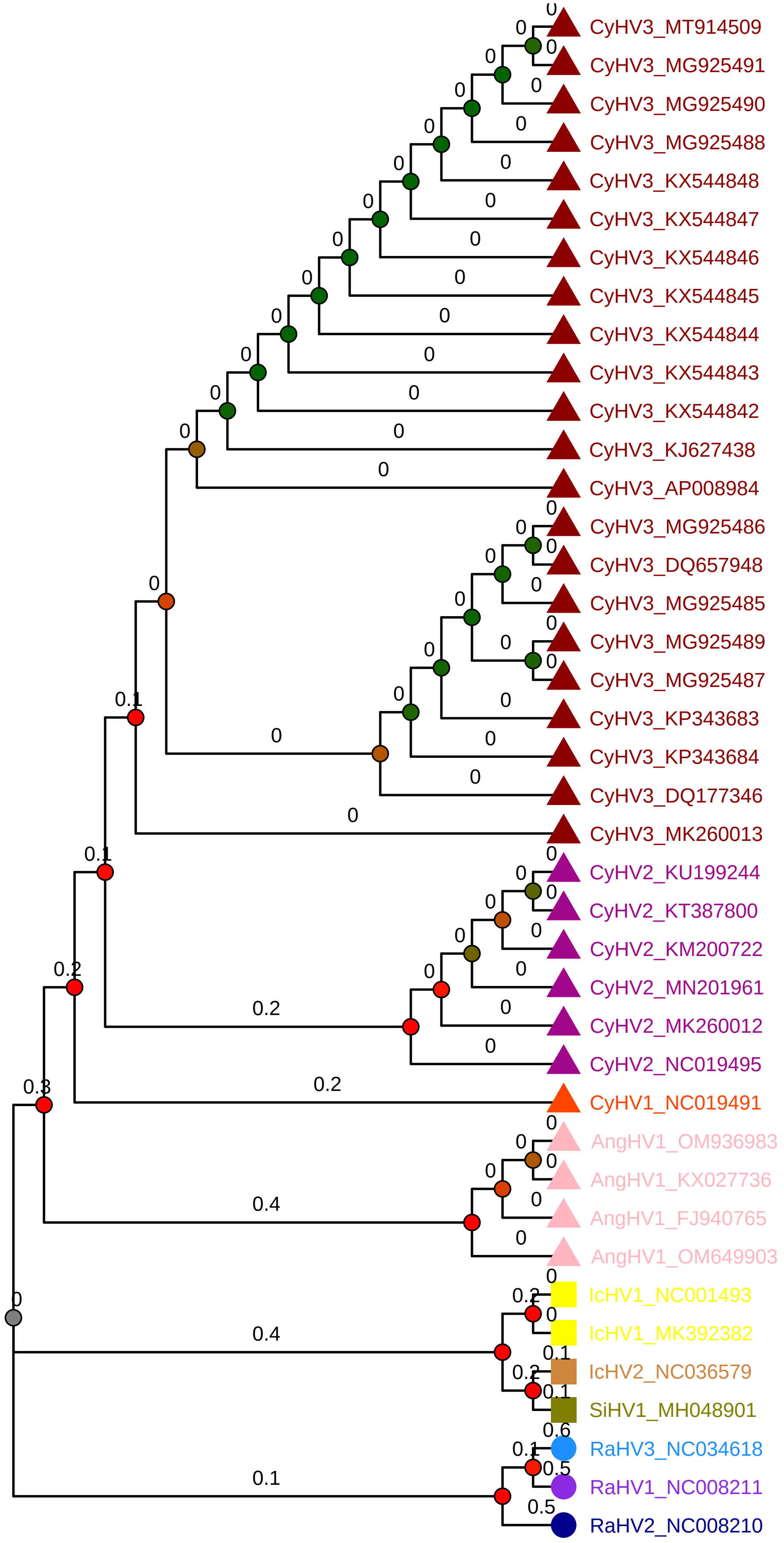

#32 NA: ML method using MEGA

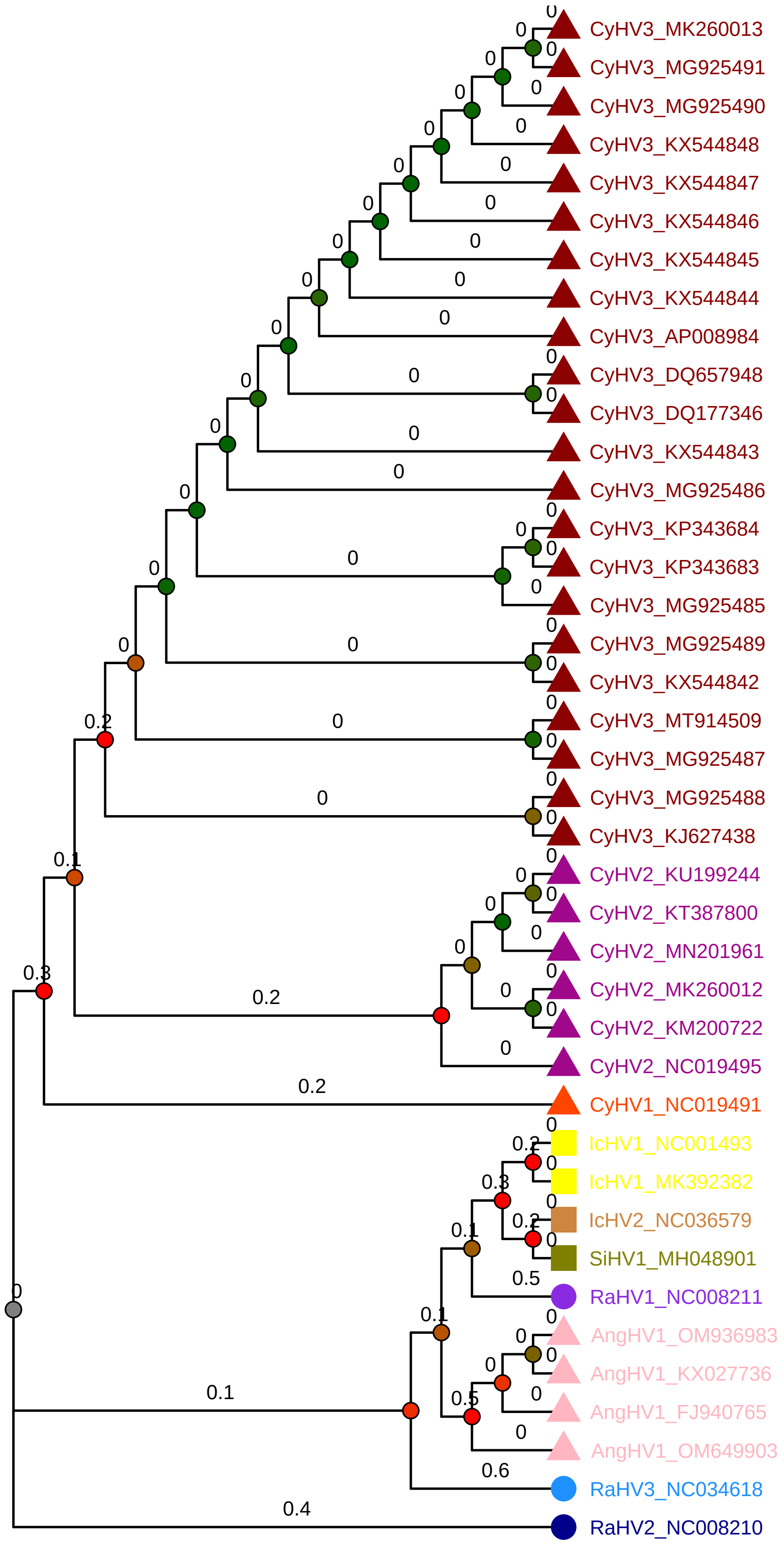

#6 AA: NJ method using MEGA

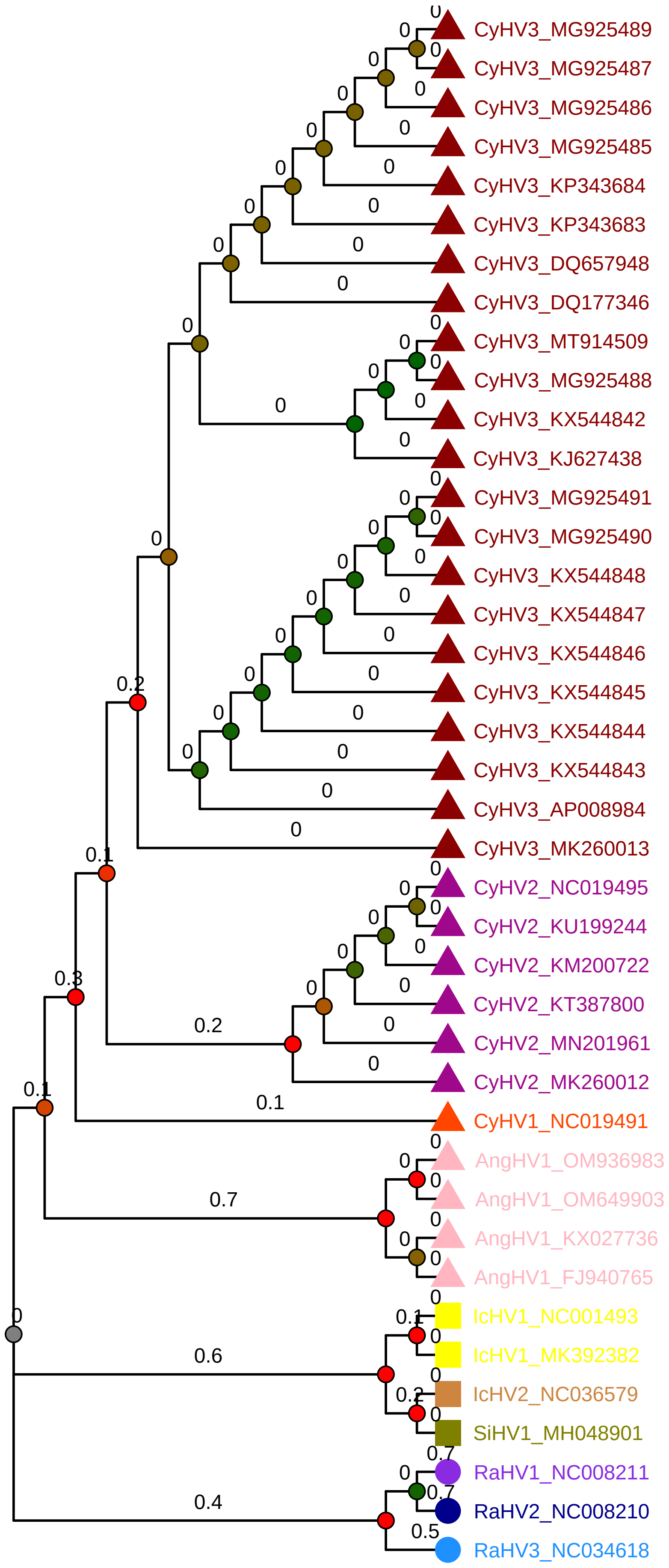

#9 AA: NJ method using MEGA

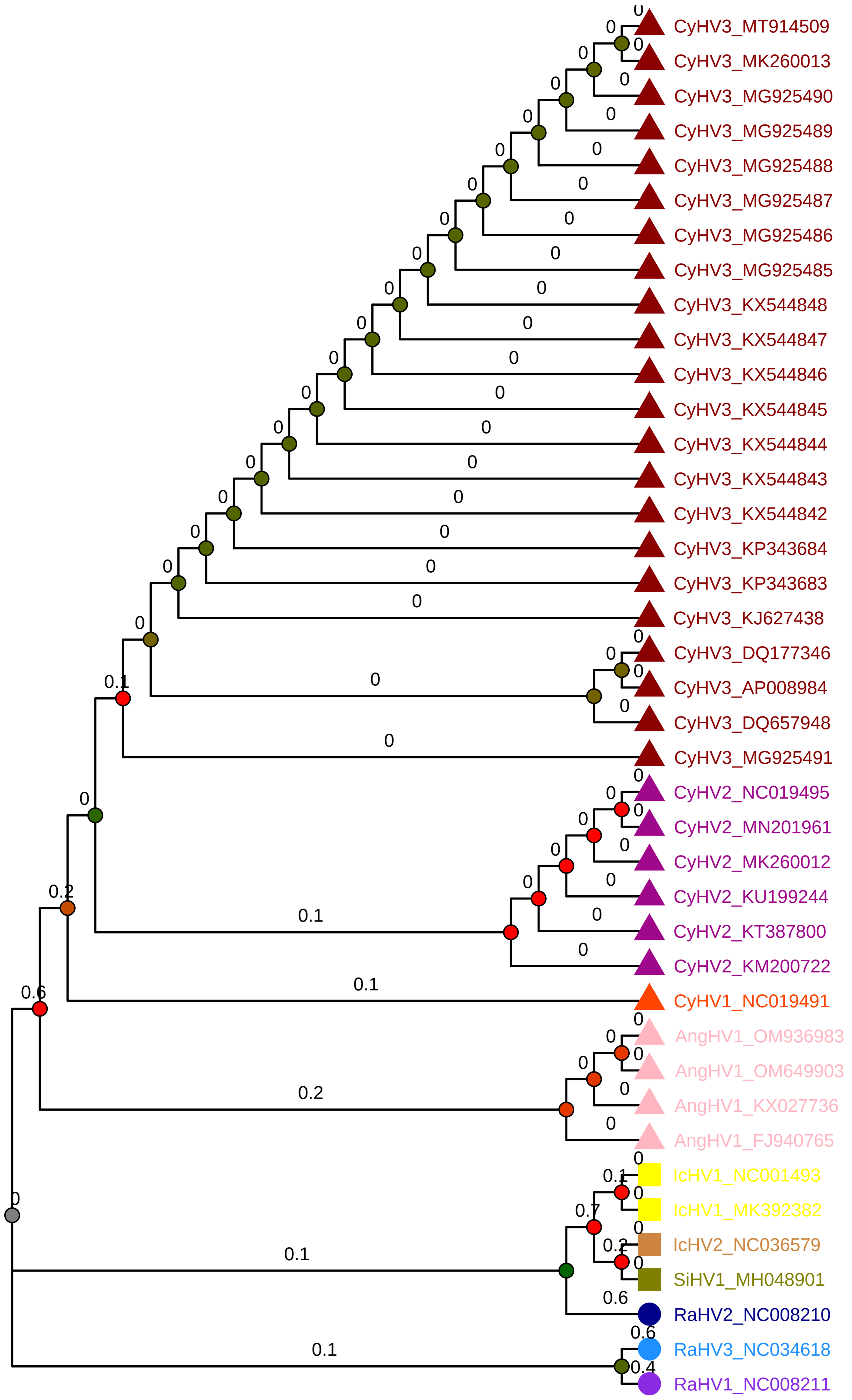

#12 AA: NJ method using MEGA

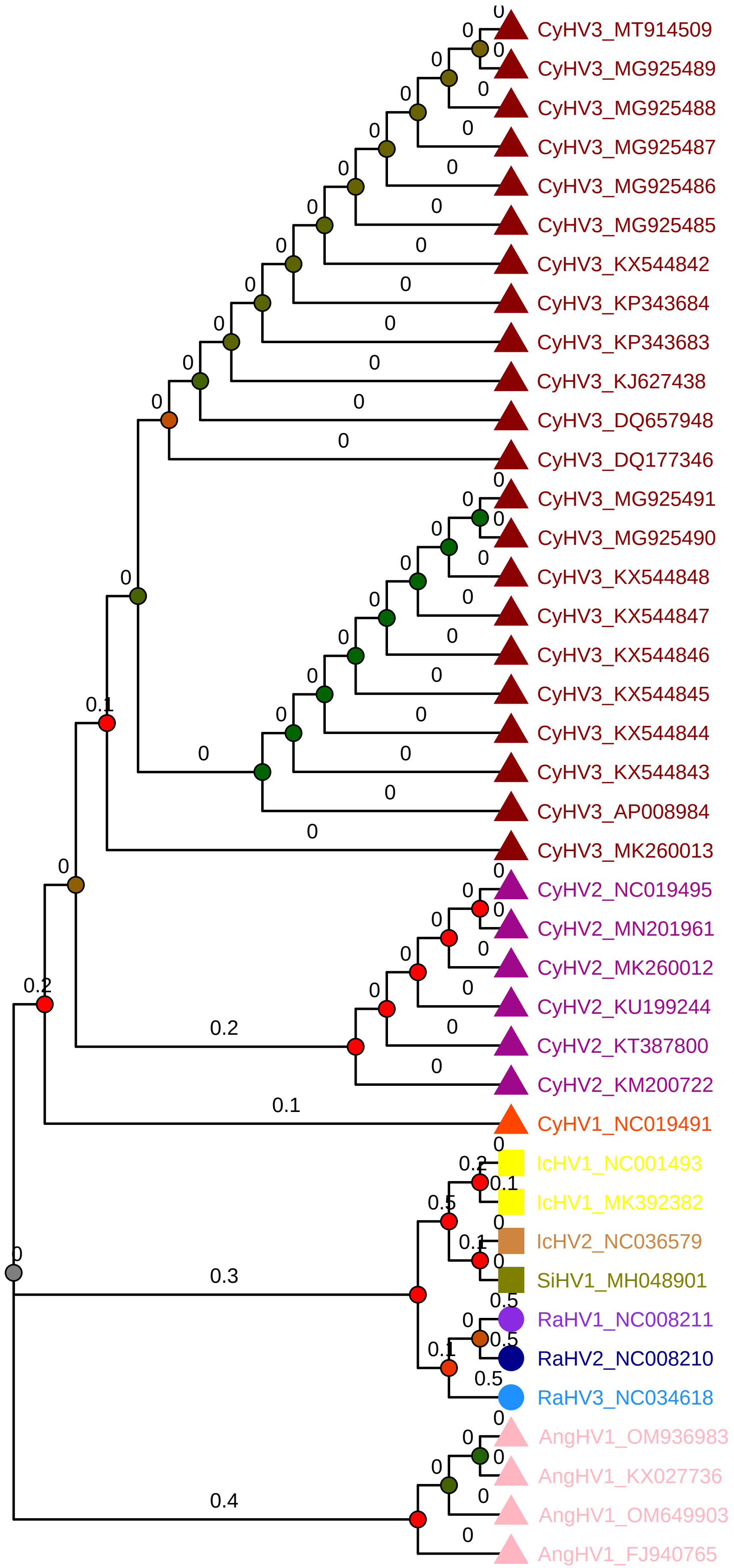

#13 AA: NJ method using MEGA

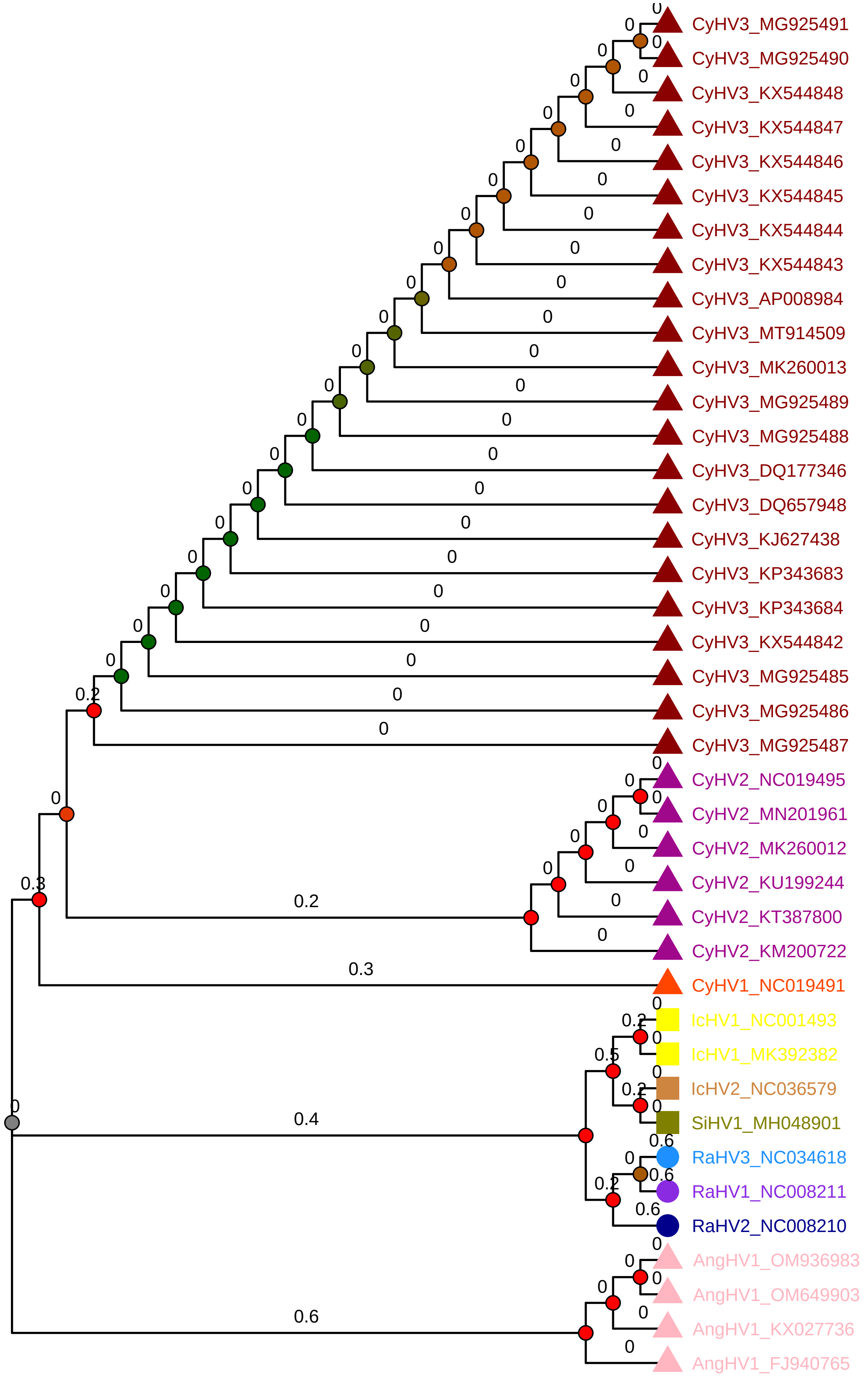

#26 AA: NJ method using MEGA

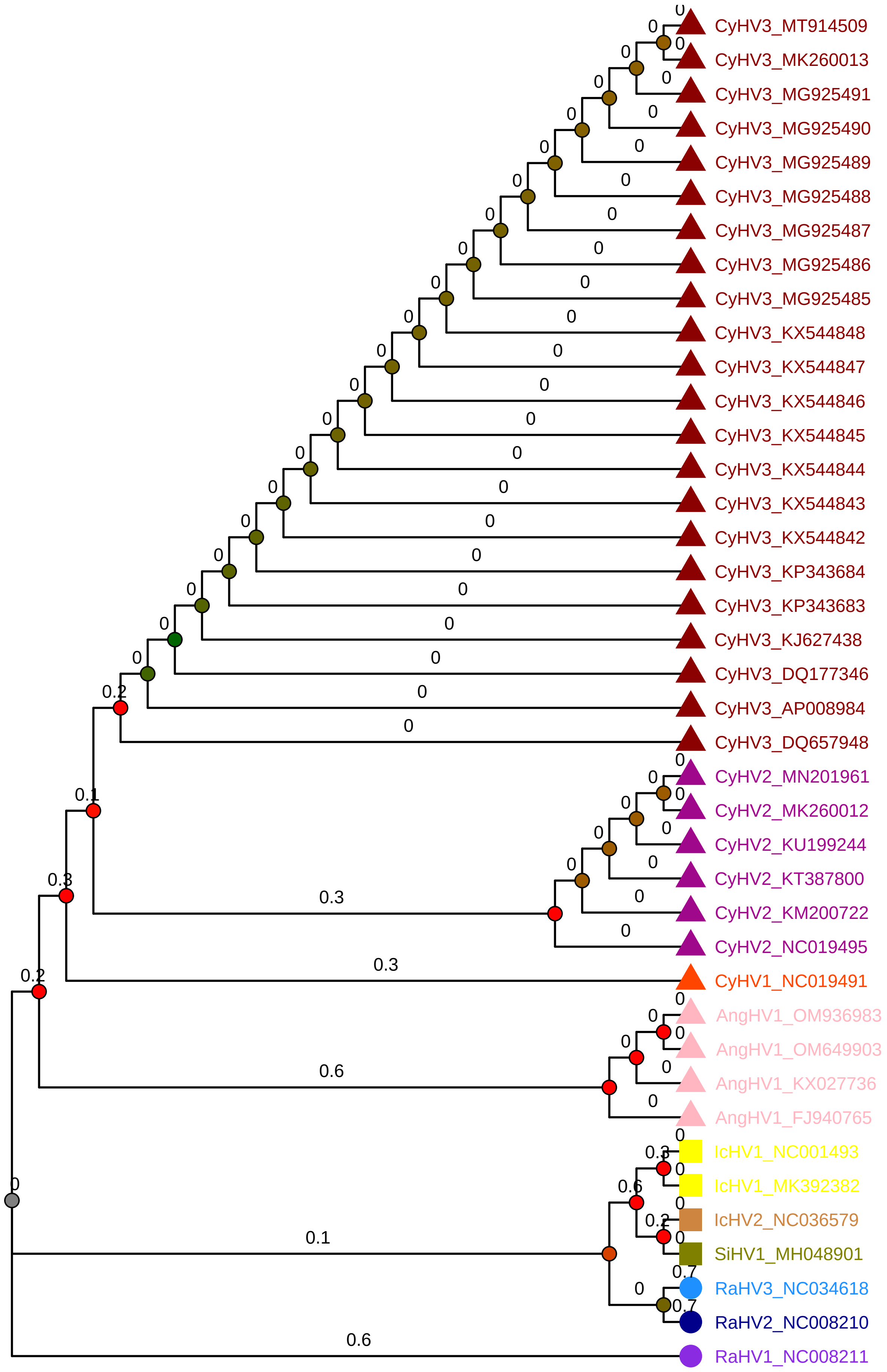

#27 AA: NJ method using MEGA

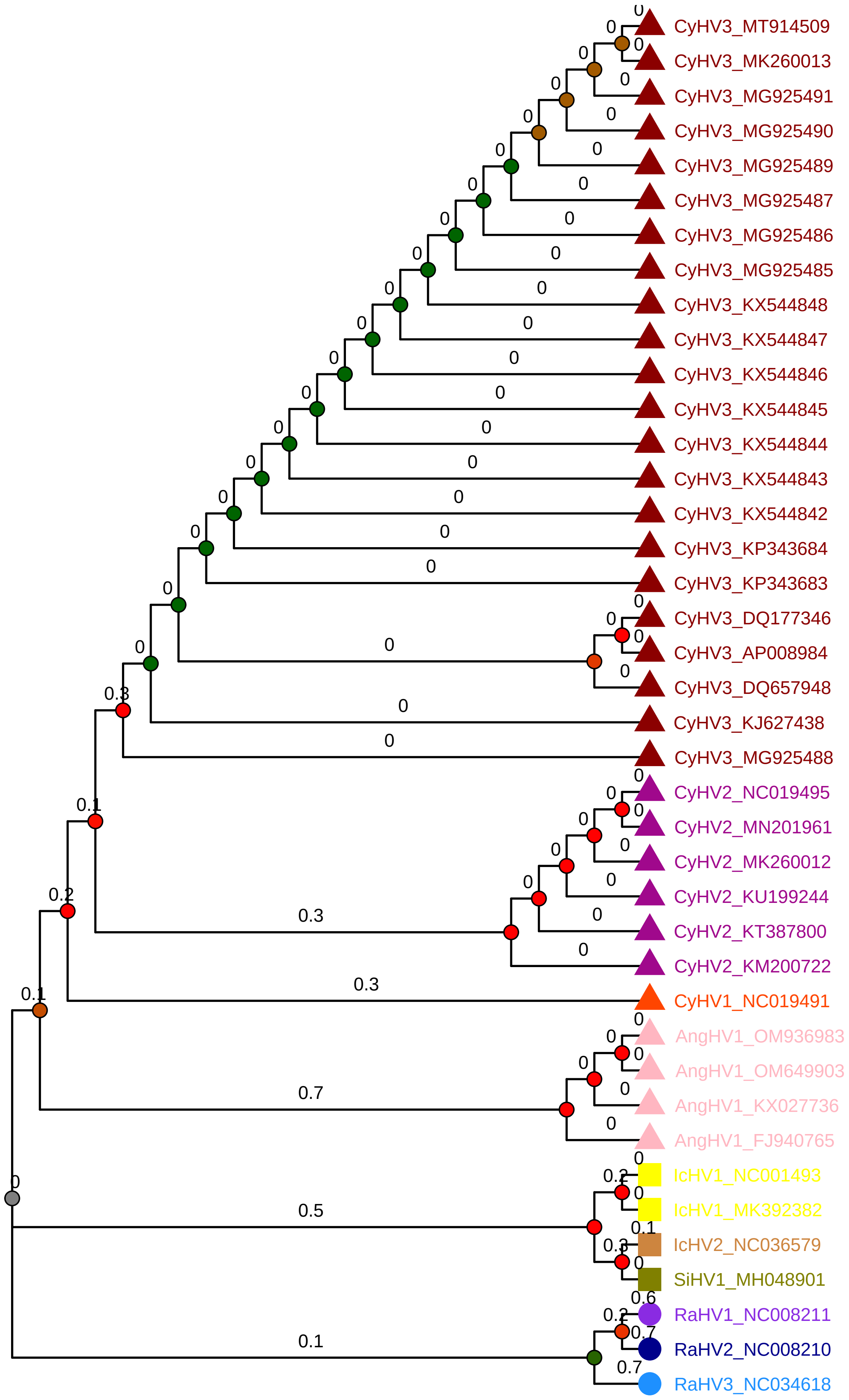

#28 AA: NJ method using MEGA

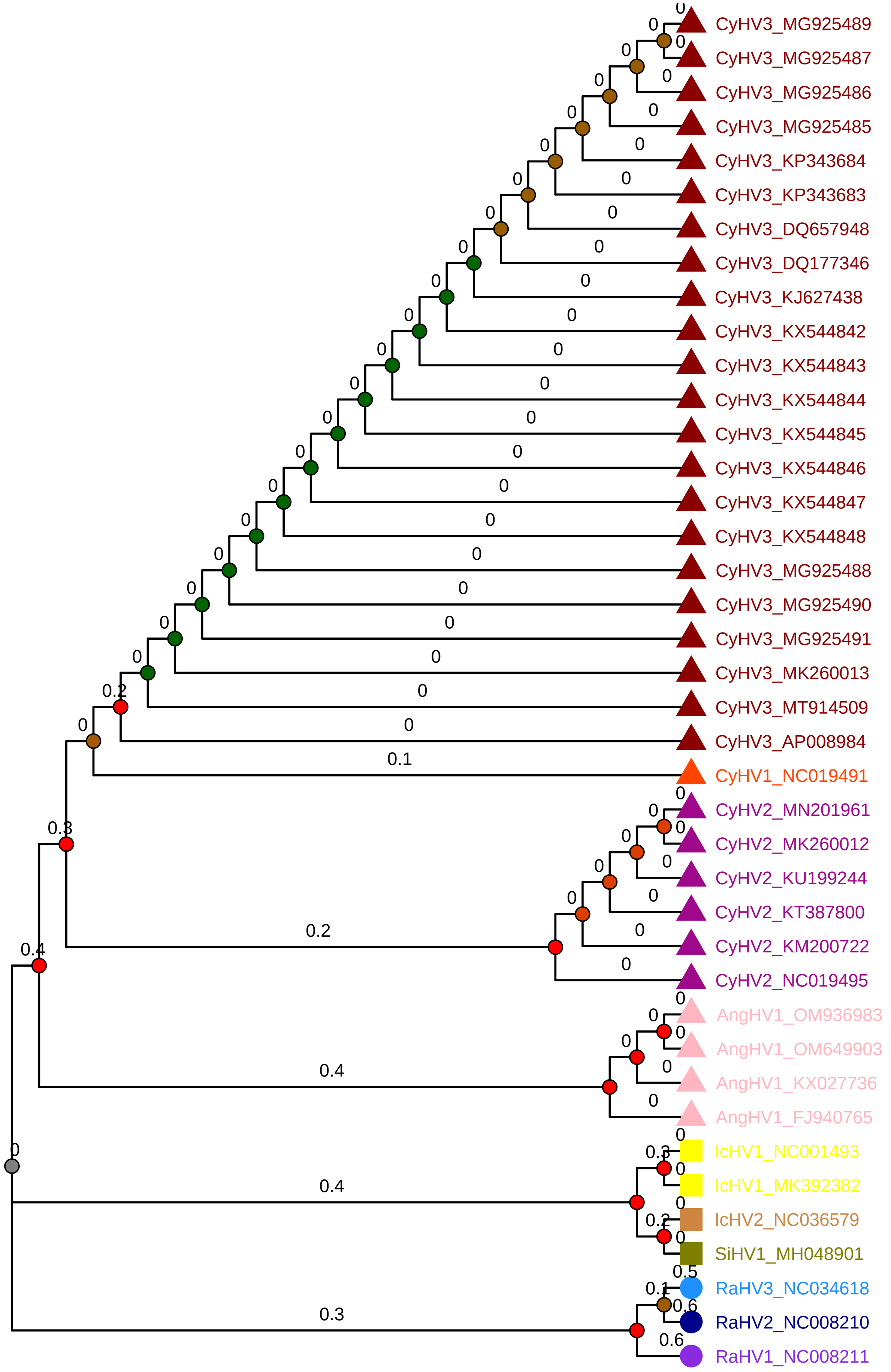

#29 AA: NJ method using MEGA

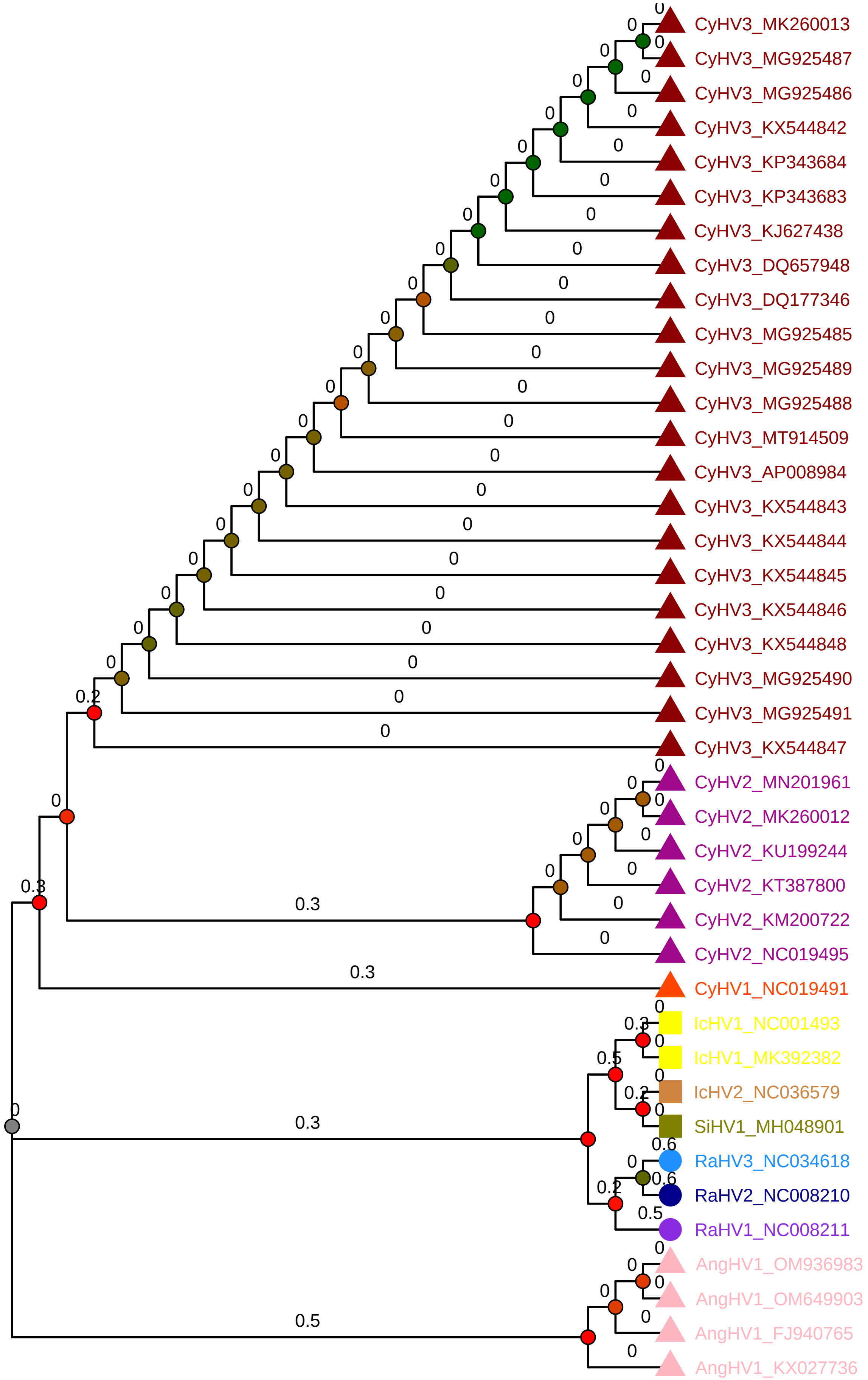

#30 AA: NJ method using MEGA

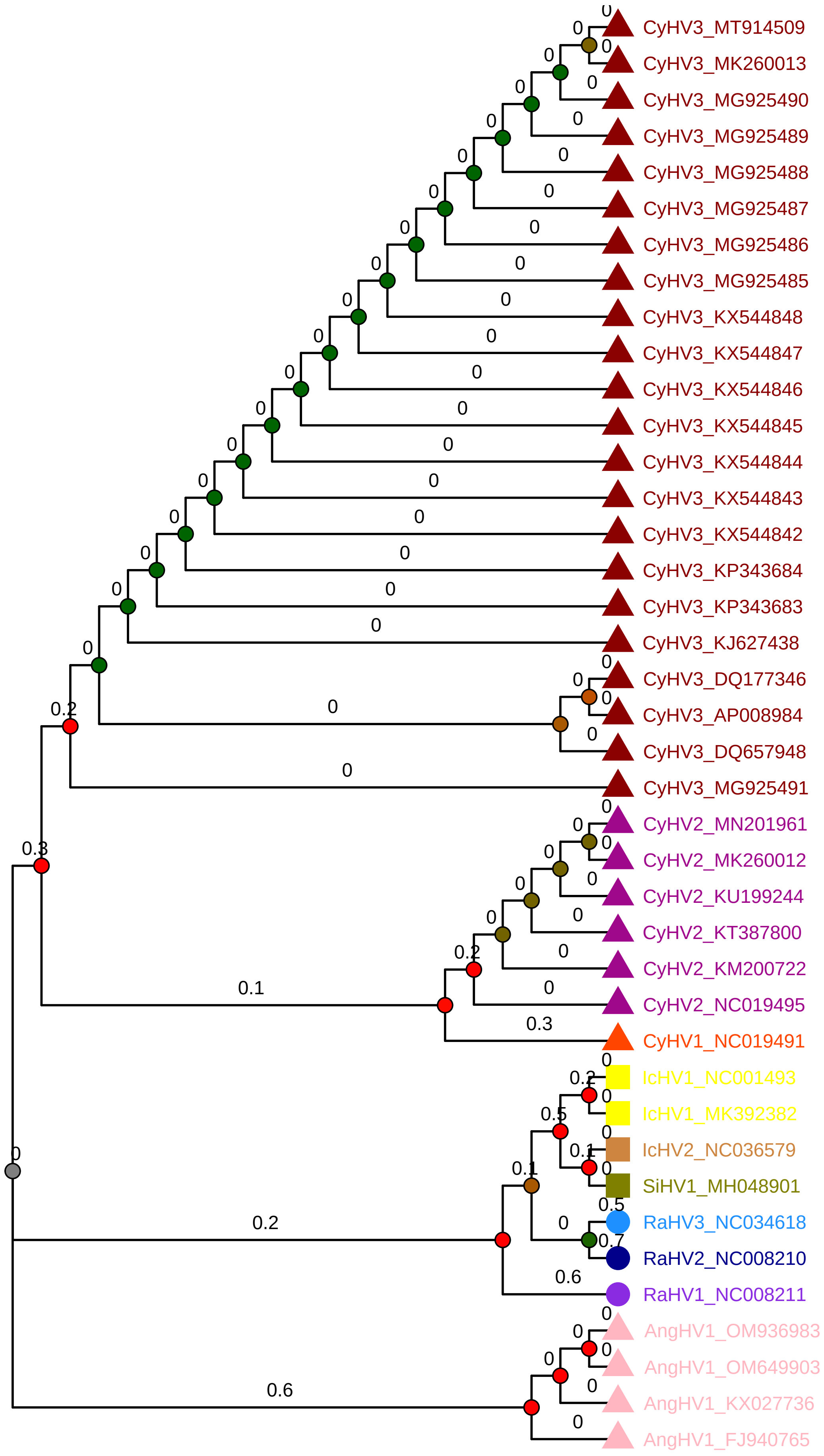

#31 AA: NJ method using MEGA

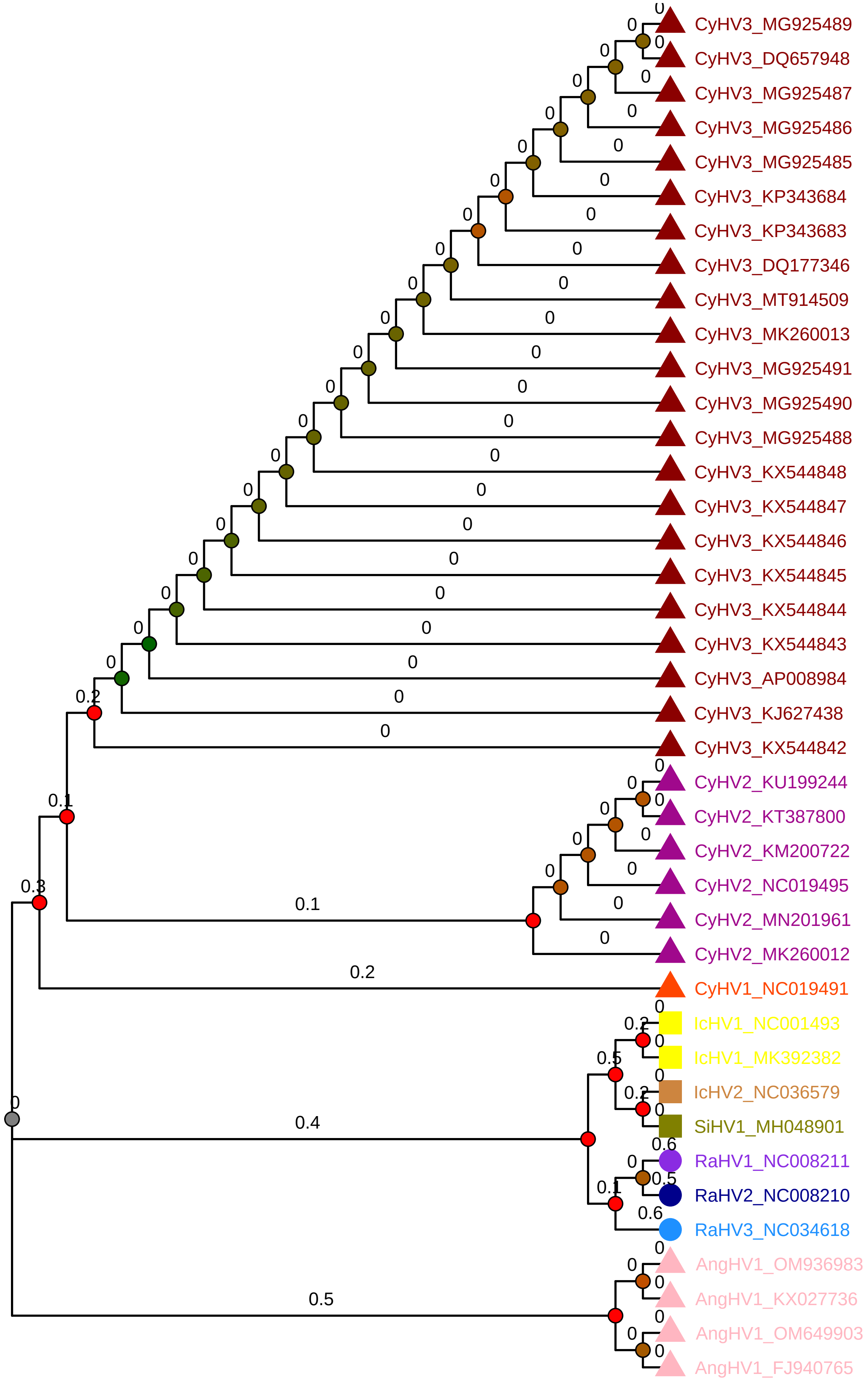

#32 AA: NJ method using MEGA

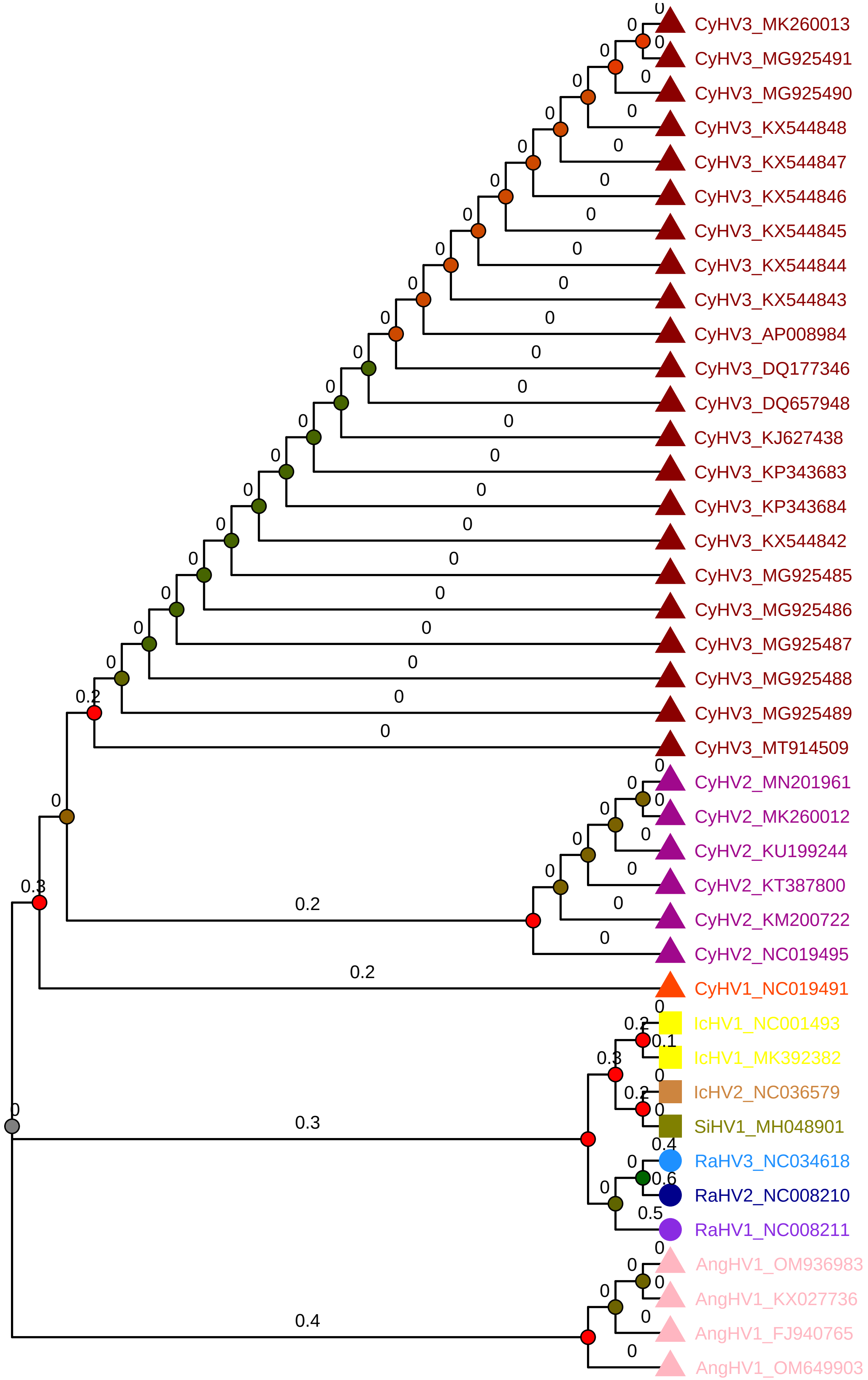

#6 NA: NJ method using MEGA

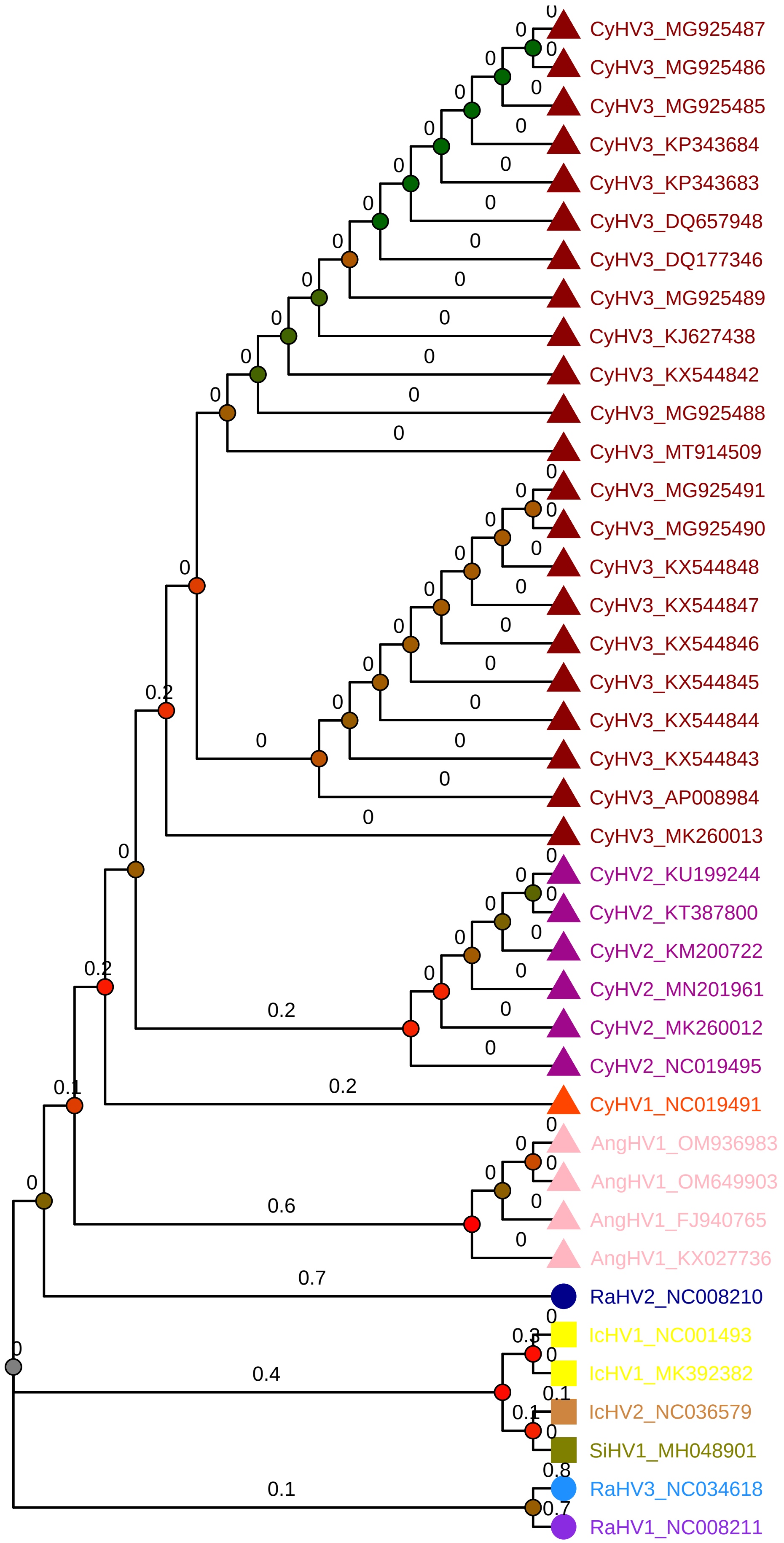

#9 NA: NJ method using MEGA

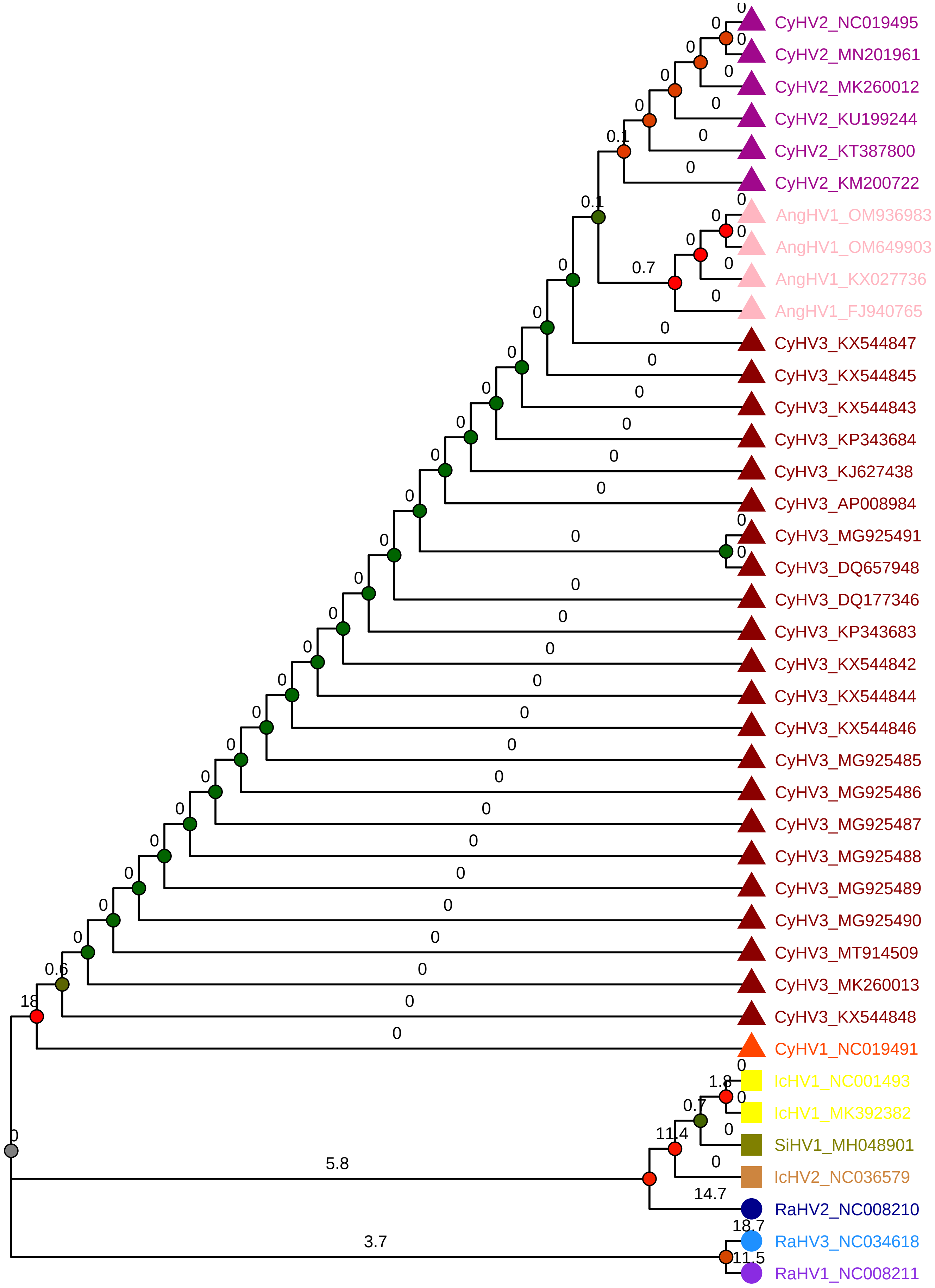

#12 NA: NJ method using MEGA

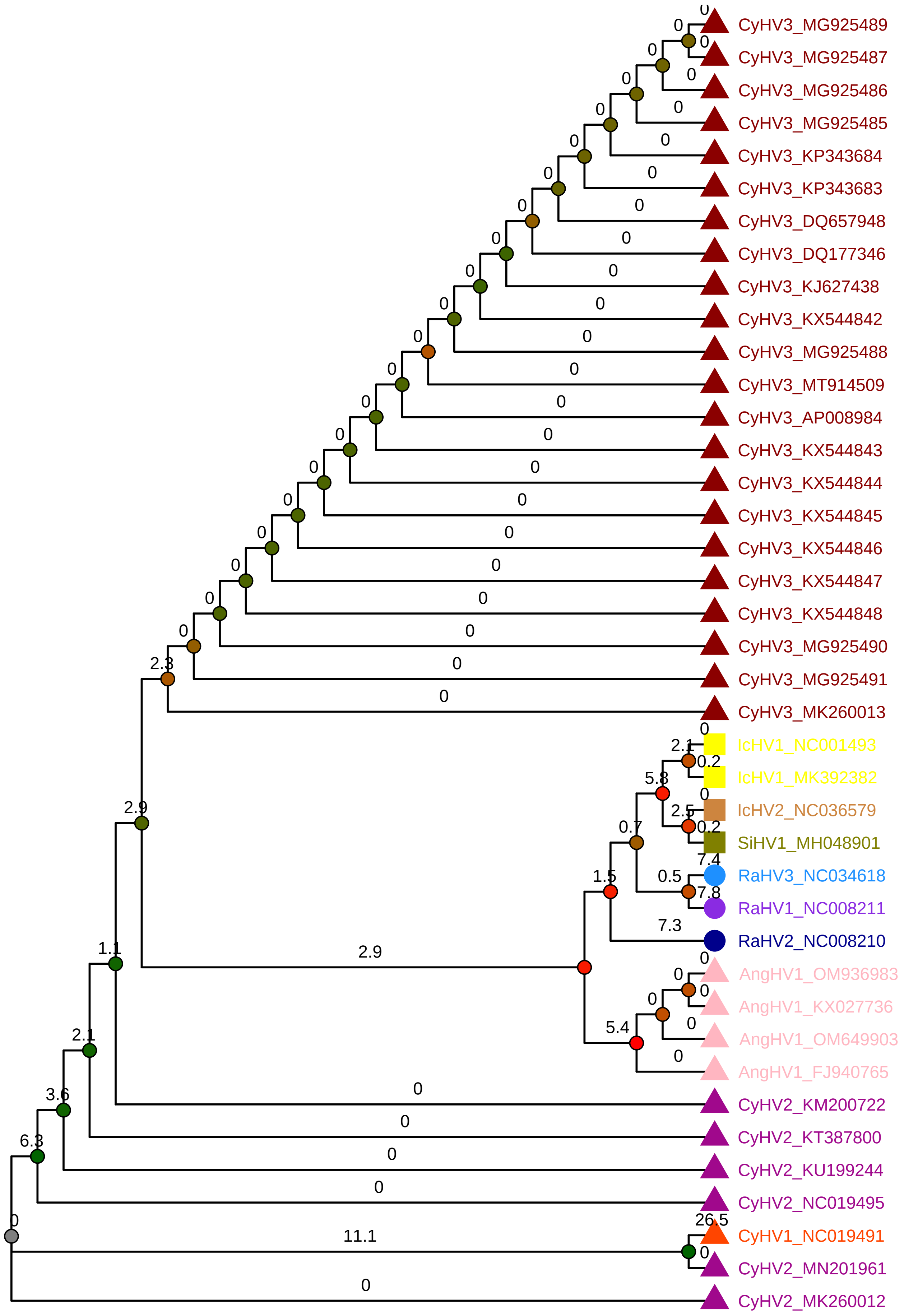

#13 NA: NJ method using MEGA

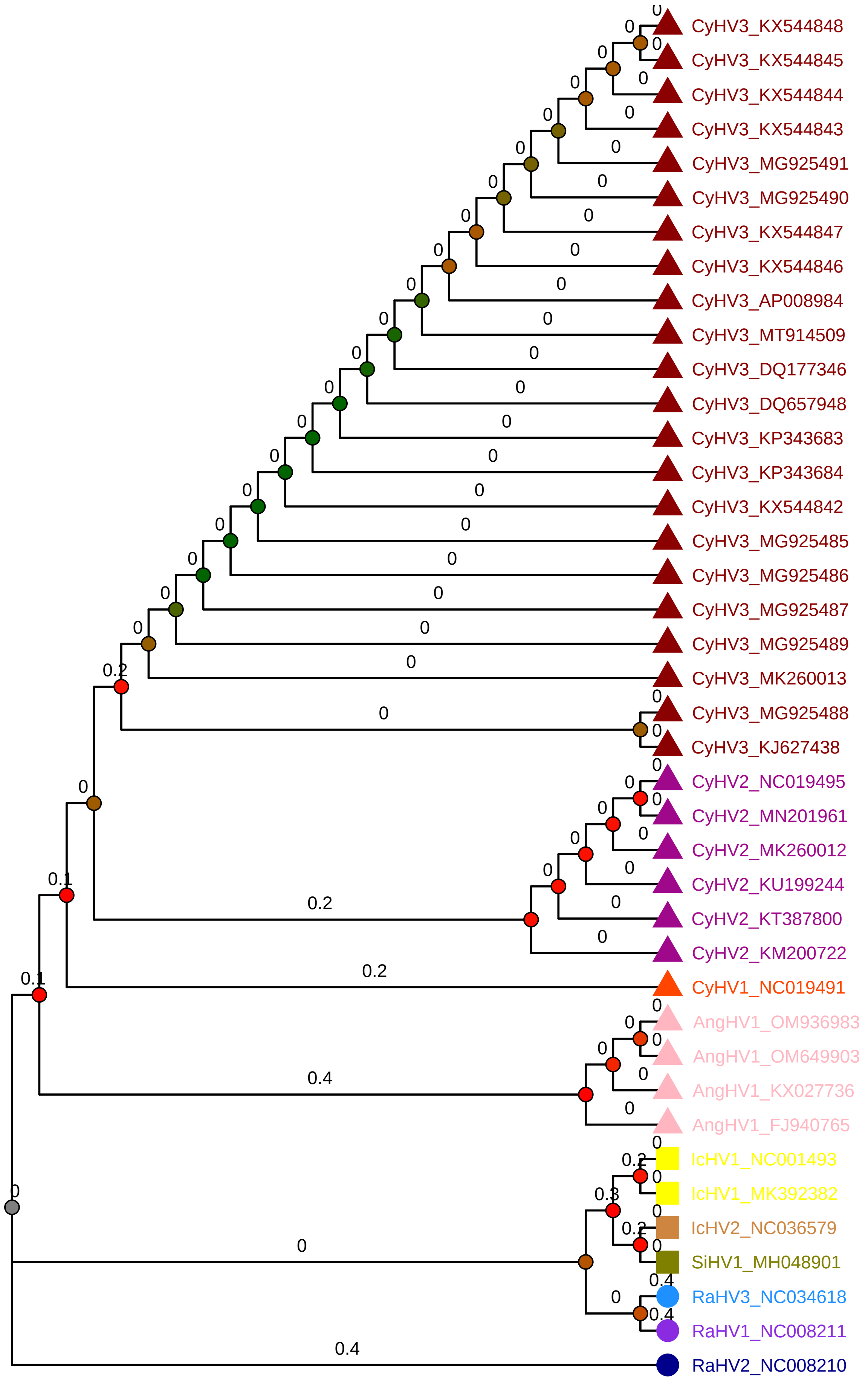

#26 NA: NJ method using MEGA

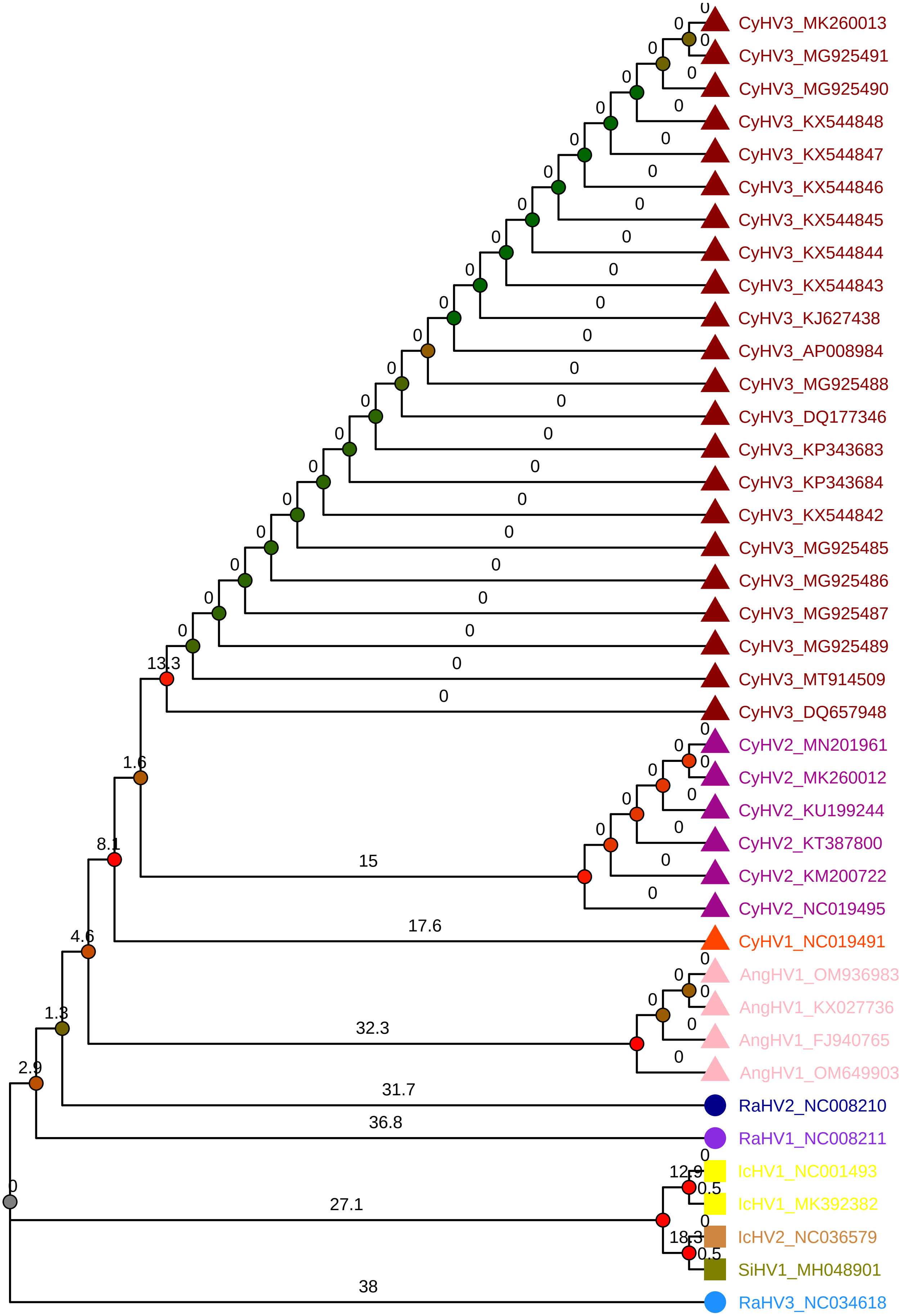

#27 NA: NJ method using MEGA

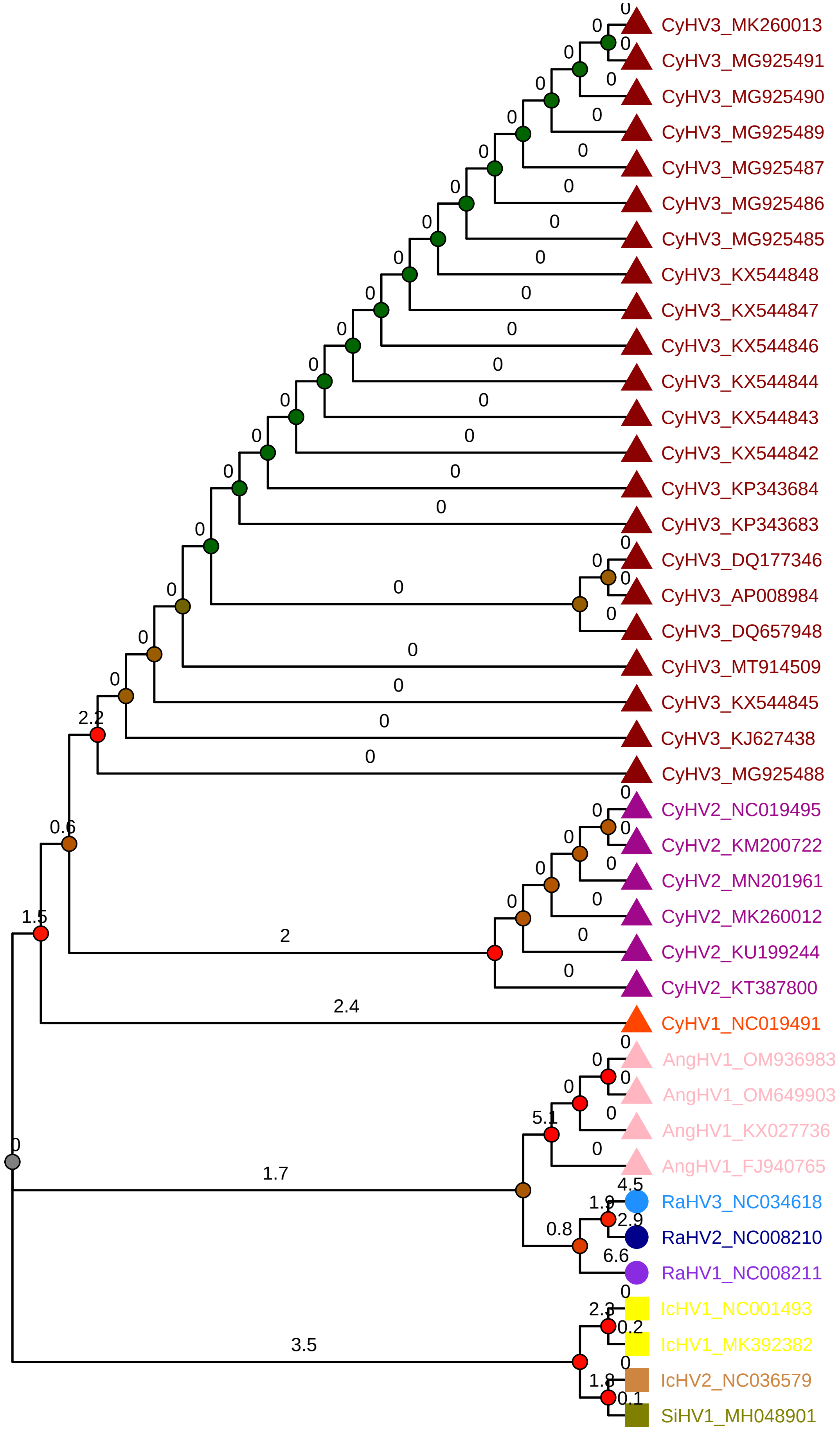

#28 NA: NJ method using MEGA

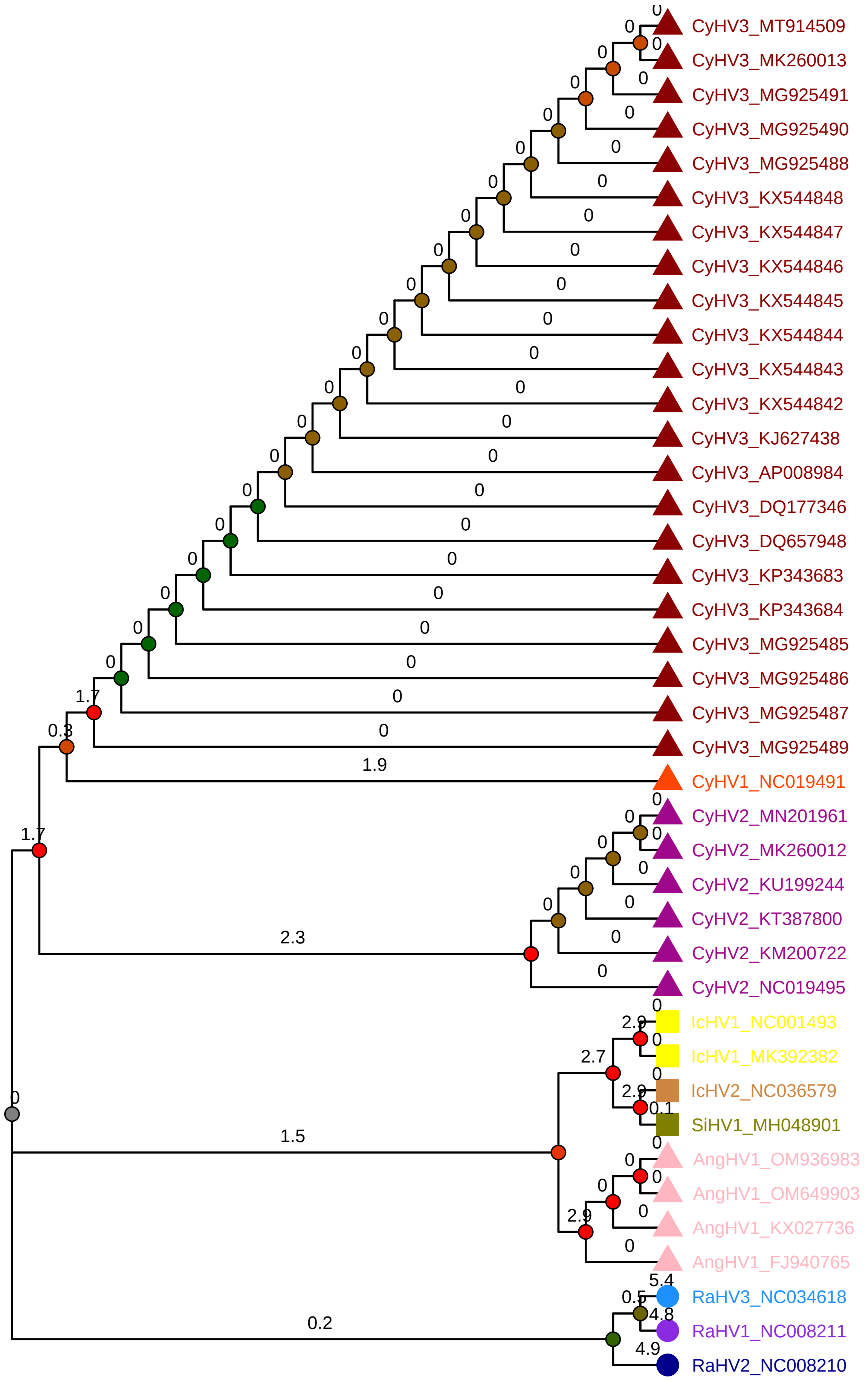

#29 NA: NJ method using MEGA

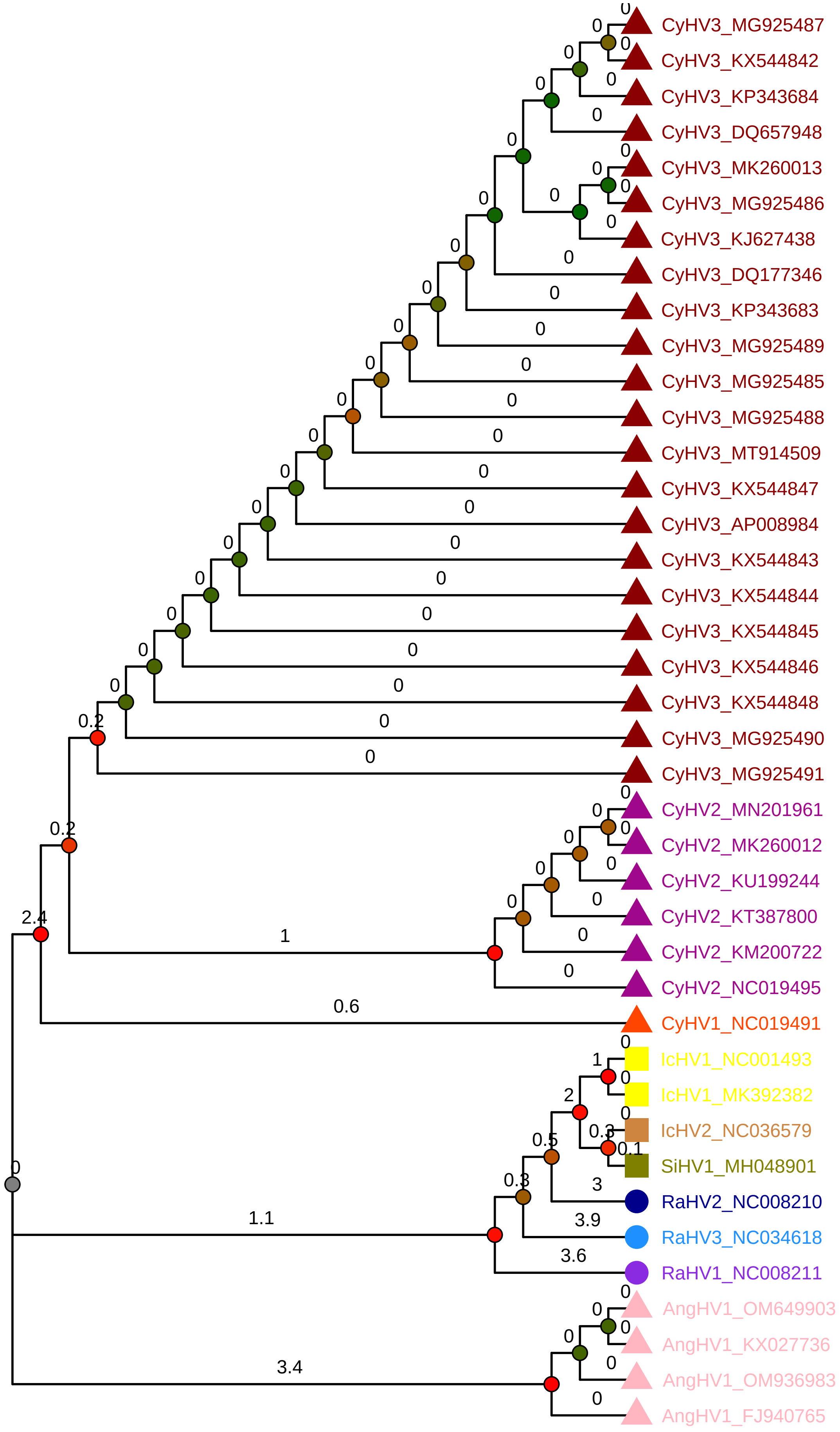

#30 NA: NJ method using MEGA

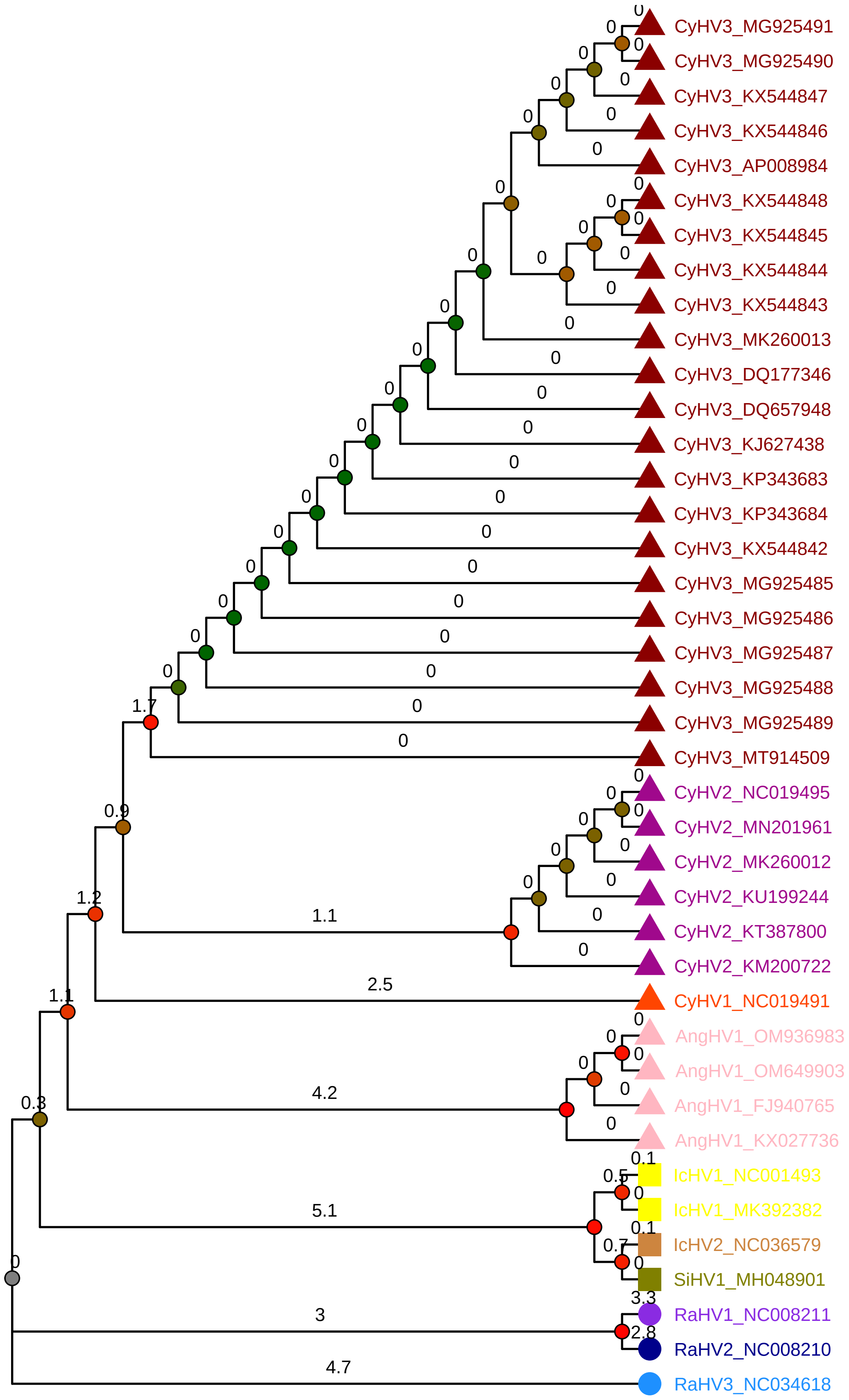

#31 NA: NJ method using MEGA

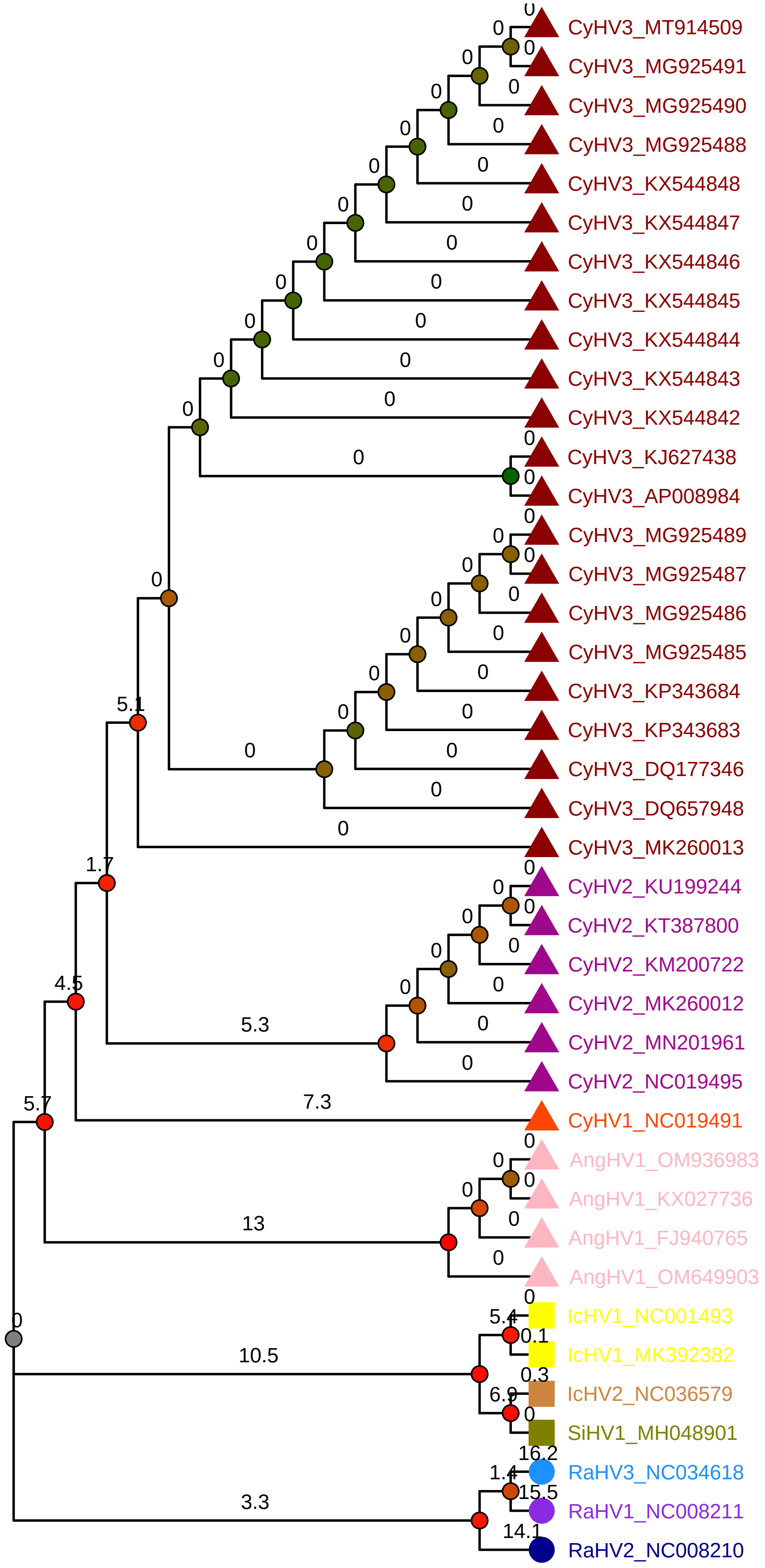

#32 NA: NJ method using MEGA

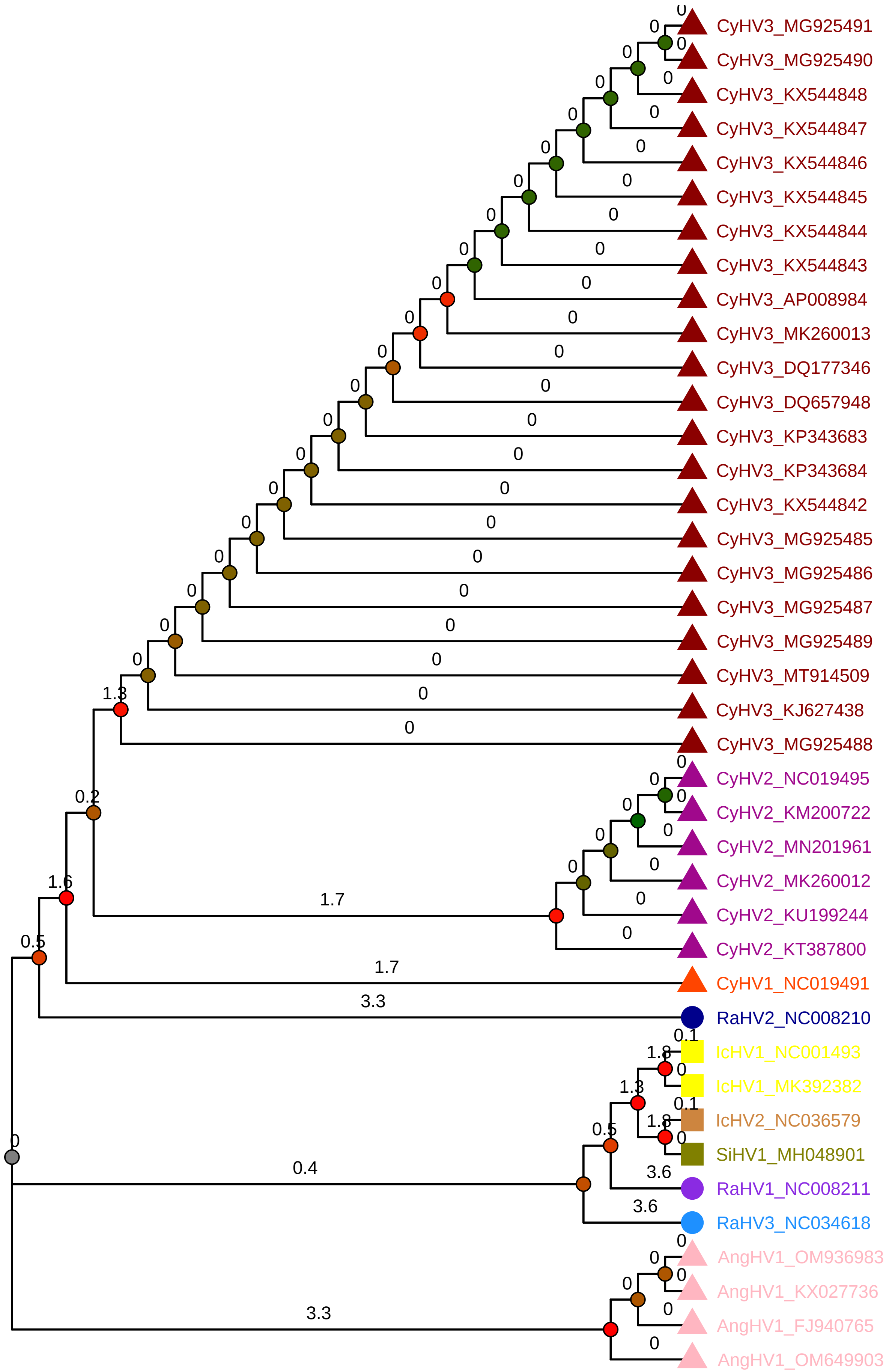

Supplement: Supplementary file 6 [file mmc6.pdf]
